# A model-based approach to estimating the prevalence of disease combinations in South Africa

**DOI:** 10.1136/bmjgh-2023-013376

**Published:** 2024-02-22

**Authors:** Leigh F Johnson, Reshma Kassanjee, Naomi Folb, Sarah Bennett, Andrew Boulle, Naomi S Levitt, Robyn Curran, Kirsty Bobrow, Rifqah A Roomaney, Max O Bachmann, Lara R Fairall

**Affiliations:** 1 Centre for Infectious Disease Epidemiology and Research (CIDER), University of Cape Town, Cape Town, South Africa; 2 Medscheme, Cape Town, South Africa; 3 Department of Health, Western Cape Provincial Government, Cape Town, South Africa; 4 Department of Medicine, University of Cape Town, Cape Town, South Africa; 5 Knowledge Translation Unit, University of Cape Town, Cape Town, Western Cape, South Africa; 6 Burden of Disease Research Unit, South African Medical Research Council, Cape Town, Western Cape, South Africa; 7 Norwich Medical School, University of East Anglia, Faculty of Medicine and Health Sciences, Norwich, UK; 8 King's Global Health Institute, King's College London, London, UK

**Keywords:** multimorbidity, multiple long-term conditions, comorbidity, non-communicable diseases, South Africa

## Abstract

**Background:**

The development of strategies to better detect and manage patients with multiple long-term conditions requires estimates of the most prevalent condition combinations. However, standard meta-analysis tools are not well suited to synthesising heterogeneous multimorbidity data.

**Methods:**

We developed a statistical model to synthesise data on associations between diseases and nationally representative prevalence estimates and applied the model to South Africa. Published and unpublished data were reviewed, and meta-regression analysis was conducted to assess pairwise associations between 10 conditions: arthritis, asthma, chronic obstructive pulmonary disease (COPD), depression, diabetes, HIV, hypertension, ischaemic heart disease (IHD), stroke and tuberculosis. The national prevalence of each condition in individuals aged 15 and older was then independently estimated, and these estimates were integrated with the ORs from the meta-regressions in a statistical model, to estimate the national prevalence of each condition combination.

**Results:**

The strongest disease associations in South Africa are between COPD and asthma (OR 14.6, 95% CI 10.3 to 19.9), COPD and IHD (OR 9.2, 95% CI 8.3 to 10.2) and IHD and stroke (OR 7.2, 95% CI 5.9 to 8.4). The most prevalent condition combinations in individuals aged 15+ are hypertension and arthritis (7.6%, 95% CI 5.8% to 9.5%), hypertension and diabetes (7.5%, 95% CI 6.4% to 8.6%) and hypertension and HIV (4.8%, 95% CI 3.3% to 6.6%). The average numbers of comorbidities are greatest in the case of COPD (2.3, 95% CI 2.1 to 2.6), stroke (2.1, 95% CI 1.8 to 2.4) and IHD (1.9, 95% CI 1.6 to 2.2).

**Conclusion:**

South Africa has high levels of HIV, hypertension, diabetes and arthritis, by international standards, and these are reflected in the most prevalent condition combinations. However, less prevalent conditions such as COPD, stroke and IHD contribute disproportionately to the multimorbidity burden, with high rates of comorbidity. This modelling approach can be used in other settings to characterise the most important disease combinations and levels of comorbidity.

**WHAT IS ALREADY KNOWN ON THIS TOPIC**

- In most countries, the most commonly occurring sources of multimorbidity include hypertension, diabetes and arthritis.
- South Africa has an exceptionally high prevalence of non-communicable diseases (NCDs), compared with other countries in Africa and globally.
- In high-income settings, HIV tends to be positively associated with NCDs.

**WHAT THIS STUDY ADDS**

- We present a novel approach to synthesising data on the prevalence of different disease combinations, which can be used to produce estimates with greater generalisability, precision and range than would be possible when relying on a single data source.
- The most frequent disease combinations in South Africa are similar to those in high-income settings.
- However, in South Africa, HIV is either not associated or negatively associated with NCDs.
- The NCDs that are associated with the highest frequency of comorbidity are chronic obstructive pulmonary disease (COPD), stroke and ischaemic heart disease (IHD).

**HOW THIS STUDY MIGHT AFFECT RESEARCH, PRACTICE OR POLICY**

- This novel approach can be applied in other settings.
- Standard OR models used in meta-analysis may be inappropriate in the context of multimorbidity research.
- Although there has been much focus on HIV and tuberculosis screening in South Africa, there is a greater need to screen for NCDs and mental health.
- The need to screen for comorbidities is greatest in patients with COPD, stroke and IHD.

## Introduction

Multimorbidity, commonly defined as having multiple long-term conditions, is increasingly recognised as a global challenge. A recent systematic review estimated that 37% of the world’s adult population is experiencing multimorbidity, with prevalence estimates ranging between 28% in Africa and 46% in South America.^1^ In order to prioritise and reorient health systems, policies and clinical practice guidelines to address this, it is important to have reliable local estimates of the prevalence of different configurations of conditions, both in the population as a whole, and in patients with specific index conditions. This would assist policy-makers in identifying the most common condition combinations on which to focus interventions, and assist healthcare workers in identifying the conditions they most need to screen for and manage, in patients with a given chronic condition.

However, national prevalence levels of different condition combinations are difficult to estimate reliably and consistently. Many studies of multimorbidity are based on medical records^2–4^ and miss most of the burden of undiagnosed and untreated disease. Many studies are conducted in specific locations,^5–7^ which are not nationally representative, or are conducted only in older adults.^7–10^ National household surveys typically rely on self-reported measures of past diagnosis or symptoms^8 9 11^ and are often underpowered to estimate the prevalence of less frequent condition combinations. Although it would be ideal to synthesise data from multiple sources, in order to improve both precision and generalisability, standard meta-analytical methods are not well suited to this task, given extensive heterogeneity in disease definitions and populations studied,^12^ and small numbers of studies for certain condition combinations. Simple modelling techniques are required to synthesise these data more effectively.

In low-income and middle-income countries (LMICs), multimorbidity is relatively under-researched.^1 12–14^ Although Africa has a low reported prevalence of multimorbidity, this may be due in part to underdiagnosis of chronic conditions.^1^ Further, Africa has a rapidly ageing population^15^ and faces an associated increase in the burden of non-communicable diseases (NCDs). This is a challenge in the context of weak and verticalised healthcare systems that are ill equipped to deliver chronic care, given a historic focus on communicable diseases and maternal and child health. The burgeoning NCD challenges may be aggravated by HIV, which is thought to increase the risk of several NCDs.^16–18^

South Africa is an important context in which to assess multimorbidity, as it is an African country with a growing NCD burden and it has one of the highest multimorbidity prevalence levels across LMICs.^11^ It has the largest population of people living with HIV globally, with an adult HIV prevalence of 18.2%^19^ compared with 0.6% globally.^20^ It has an adult diabetes prevalence of 10.2%,^21^ compared with an average of 4.2% in sub-Saharan Africa.^22^ Compared with other sub-Saharan African countries, it also has high levels of hypertension^23 24^ and arthritis,^22 25^ both of which are linked to high rates of obesity.^26^ Tuberculosis (TB) prevalence is roughly double the African average,^27 28^ and although conventionally considered an acute condition, is associated with substantial long-term morbidity (including structural lung disease and chronic obstructive pulmonary disease (COPD)).^29–31^ South Africa is also an important setting in which to assess novel methods for data synthesis, as it has a large body of multimorbidity data. Previous reviews of South African NCD data have focused on specific conditions (diabetes,^32^ arthritis,^25^ stroke and coronary heart disease^33^) and NCD surveillance,^34^ but multimorbidity data have only been synthesised narratively.^35^

The objective of this study is to propose a novel statistical framework for integrating epidemiological evidence from different sources, to produce nationally representative estimates of the prevalence of different condition combinations. We apply the approach to South Africa and discuss how the results may be used to inform public health policy. This study was undertaken as part of a wider effort to reorientate primary healthcare systems,^36 37^ guidelines^38^ and health worker training^39^ to better manage the growing numbers of people living with multiple conditions, ensuring that we address the leading combinations of multimorbidity in our context.

## Methods

We followed a three-step process to estimate the prevalence of different condition combinations in South Africa: (1) estimating the strength of association between different conditions, based on meta-analyses of previous South African studies, (2) estimating the prevalence of individual conditions, based on nationally representative data sources and (3) combining the results of steps (1) and (2) using a statistical model that estimated the national prevalence of each condition combination. The analysis was limited to conditions that fulfilled three criteria: they appeared in a recently recommended ‘core list’ of conditions to include in studies of multimorbidity^12^; there were nationally representative estimates of their prevalence; and there were at least four South African studies/datasets reporting on their association with other conditions. The 10 conditions that satisfied all criteria were arthritis, asthma, COPD, depression, diabetes, HIV, hypertension, ischaemic heart disease (IHD), stroke and TB (online supplemental file 1 table S1.1 provides more detail on the application of the three criteria).

10.1136/bmjgh-2023-013376.supp1 Supplementary data

### Step 1: estimating the strength of association between conditions

We conducted a review of published South African epidemiological studies investigating multimorbidity in adults. We searched PubMed and Web of Science for articles that included the terms ‘South Africa’ and ‘multimorbidity’. Studies were also identified by examining reference lists of recent systematic reviews in South Africa of multimorbidity,^35^ diabetes,^32^ arthritis,^25^ TB,^29^ COPD,^40^ and stroke and coronary heart disease.^33^ Studies were included if they reported the prevalence of at least two of the ten listed conditions, as well as the proportions of people with any combination of two conditions, in adults or predominantly adult groups. Where multiple studies reported on the same dataset, we avoided double-counting by including only one set of estimates for each condition combination.

We augmented these published data sources with datasets for which it was possible to extract individual-level data on the prevalence of chronic conditions, provided these included at least four of the conditions of interest and did not overlap with the published data. Four datasets were identified:

1. The Medscheme database: Medscheme is one of the largest private medical scheme administrators in South Africa. Data have previously been analysed to assess, for example, the incidence of diabetes and mental disorders in people living with HIV.^41 42^ Analysis was limited to active members aged 15 and older in January 2022.
2. The Western Cape Provincial Health Data Centre (PHDC) database: This is a database of all people who have accessed public health facilities in the Western Cape province. All medical records within the public sector are linked through a single unique patient identifier, including data from disease registers, pharmacy data, laboratory data and other sources.^43^ Prevalence was calculated in individuals aged 15 and older in January 2022, who had any public health service contact over the last 5 years (2017–2022).
3. The 2016 Demographic and Health Survey (DHS) data: The survey results are reported elsewhere,^44^ but prevalence estimates for specific condition combinations have not previously been reported.
4. The 2003 World Health Survey (WHS): As with the 2016 DHS, overall results have been published,^11 45^ but no prevalence estimates for specific condition combinations have been published.

The Medscheme and PHDC datasets were analysed cross-sectionally, with the prevalence of each condition being defined in terms of the cumulative incidence of the condition.

For each included study or dataset, an unadjusted OR was calculated to represent the strength of association between each pair of reported conditions, based on 2×2 contingency tables. A random effects meta-analysis was performed to pool the ORs for each pair of conditions. In cases where three or more ORs were estimated for a given pair of conditions, a meta-regression was performed to assess the dependence of the OR on the product of the prevalence of the first condition and the prevalence of the second condition (the product represents the ‘expected prevalence’ of the combination if the conditions occurred independently). This meta-regression was performed because it was anticipated that differences in condition prevalence across studies/datasets would be an important source of heterogeneity in ORs (see online supplemental file 2). In the case of the TB-depression association, no local evidence could be obtained so we relied instead on ORs from a systematic review of studies in LMICs.^46^ All meta-analyses and meta-regressions were performed using STATA V.17.0 (StataCorp). Secondary analyses were conducted to assess the effect of limiting analysis to studies/data for adults aged 50 and older; due to the smaller number of datasets and greater similarity of ORs in this analysis, pooled ORs were estimated using meta-analysis rather than meta-regression. Further analyses were conducted using Medscheme data, to assess whether ORs changed substantially when controlling for age, and excluding studies of patients attending health facilities, in line with previous reviews.^1 47 48^

10.1136/bmjgh-2023-013376.supp2 Supplementary data

### Step 2: estimating the prevalence of individual conditions, at a national level

Nationally representative prevalence estimates for the population aged 15 and older were obtained from a number of sources. For HIV and TB, we used estimates from locally developed mathematical models that had been calibrated to multiple nationally representative datasets (surveys, vital registration data and routine health systems data).^19 49^ For hypertension and diabetes, we used clinically measured estimates from the 2012 South African National Health and Nutrition Examination Survey (SANHANES).^21^ Diabetes was defined by a haemoglobin A1c (HbA1c)≥6.5% or a self-report of current diabetes medication, and hypertension was defined by a systolic blood pressure ≥140 mm Hg, diastolic blood pressure ≥90 mm Hg or self-report of current blood pressure medication.^21^ Stroke and IHD were defined by self-report (of past stroke or past heart attack/chest pains, respectively), again as measured in the 2012 SANHANES survey.^21^ Arthritis was defined by self-report of a previous diagnosis, as measured in the 2003 WHS,^11^ after reweighting using the 2021 South African population age distribution. Asthma was also defined by self-report of a previous diagnosis, as measured in the 2016 DHS.^44^ COPD was defined by a cough with phlegm of at least 3 months duration, as measured in the 2016 DHS.^44^ Depression was defined in accordance with the Diagnostic and Statistical Manual for Mental Disorders fourth edition criteria, as measured in the 2002–2004 South African Stress and Health study with the WHO Composite International Diagnostic Interview, that is, based on self-reported symptoms of depression over the last 12 months.^50^ For the purpose of age-specific secondary analyses, estimates were obtained separately for the 15–49 and the 50 or older populations.

### Step 3: estimating the prevalence of pairwise combinations at a national level

If the national prevalence of conditions *i* and *j* are $P_{i}$ and $P_{j}$, respectively, and the proportion of the national population with both conditions *i* and *j* is $P_{ij}$, then the OR for the association between conditions *i* and *j* is

$$\theta_{ij}=\frac{P_{ij}\left(1-P_{i}-P_{j}+P_{ij}\right)}{\left(P_{i}-P_{ij}\right)\left(P_{j}-P_{ij}\right)}$$

Because the prevalence levels $P_{i}$ and $P_{j}$ are known (from step 2) and the OR is also known (from step 1), the prevalence of the condition combination can be calculated by expressing the above equation as a quadratic in $P_{ij}$, and solving for $P_{ij}$^51^ :

$$P_{ij}=\frac{\sqrt{{\left(1+\left(\theta_{ij}-1\right)\left(P_{i}+P_{j}\right)\right)}^{2}-4\left(\theta_{ij}-1\right)\theta_{ij}P_{i}P_{j}}-1-\left(\theta_{ij}-1\right)\left(P_{i}+P_{j}\right)}{2\left(1-\theta_{ij}\right)}$$

The prevalence of condition *j*, in individuals with condition *i*, is then $P_{ij}/P_{i}$.

Where meta-regression was performed in step 1, the expected OR was calculated as $\theta_{ij}=exp\left(mP_{i}P_{j}+c\right)$, where *m* and *c* are the slope and constant outputs, respectively, from the meta-regression model. Parametric bootstrapping was performed to calculate 95% CIs. For each bootstrap iteration, values of $P_{i}$ and $P_{j}$ were randomly sampled from the uncertainty ranges (assuming normally distributed values), and where the OR was estimated by simple meta-analysis, we similarly sampled from the estimated uncertainty range for $log\left(\theta_{ij}\right)$. Where the OR was calculated by meta-regression, we instead sampled values of *m* and *c* from the estimated uncertainty ranges, assuming a multivariate normal distribution and accounting for the covariance between the two parameters.

The expected number of comorbidities in individuals with condition *i* was calculated as

The variance of $M_{i}$ was approximated as the sum of the variances of the $P_{ij}/P_{i}$ terms, as estimated in the bootstrapping step (assuming independence). A log-linear regression model was fitted to assess the association between the expected number of comorbidities (natural log scale) and the prevalence of each condition. All data, STATA code and formulas used in bootstrapping are included in an Excel workbook (online supplemental file 3).

10.1136/bmjgh-2023-013376.supp3 Supplementary data

## Results

In addition to the four data sources from which we extracted individual-level data, we identified 17 South African datasets (reported in 20 studies) from which it was possible to calculate the prevalence of multiple conditions as well as the prevalence of at least one condition combination, giving a total of 21 datasets (table 1, with further details on the search results in online supplemental file 1, section 1). Of the 21 datasets, 7 were from nationally representative household surveys, and 5 were based on patients attending public health facilities. All estimated the prevalence of chronic conditions in 1998 or later. Diabetes and hypertension were the most commonly estimated conditions (each assessed in 16 of the 21 datasets), while arthritis was the least frequently estimated condition (in four datasets). Definitions of conditions varied across studies (online supplemental file 1, table S1.1).

**Table 1:** Studies included in meta-analysis

Study	Population/survey	Years	Chronic conditions included									
			Arthritis	Asthma	COPD	Depression	Diabetes	HIV/AIDS	Hypertension	IHD/angina	Stroke	Tuberculosis
Chang *et al*, Pengpid and Peltzer^7 73^	Adults aged 40+in Agincourt	2014–15			✓	✓	✓	✓	✓	✓	✓	
Corbett *et al*^74^	Mineworkers	2000–01						✓				✓
Ehrlich *et al*^75 76^	People aged 15+ (DHS national survey)	1998		✓	✓							✓
Folb *et al*^55^	Adults attending public clinics, Eden district	2011			✓	✓	✓		✓			
Garin *et al*, Negin *et al*^23 77^	Adults aged 50+ (SAGE national survey)	2007	✓	✓	✓	✓	✓	✓	✓	✓	✓	
Grimsrud *et al*^50^	Adults aged 18+ (SASH national survey)	2002–04				✓			✓			
Jithoo^78^	People aged 40+, Cape Town	2005		✓	✓							✓
Lalkhen and Mash^79^	People attending public clinics in four provinces	2010	✓	✓	✓		✓		✓		✓	
Middelkoop *et al*^80^	People aged 15+, Cape Town	2005, 2008						✓				✓
Oni *et al*^56^	Adults aged 18+, attending public clinics in Cape Town	2012–13					✓	✓	✓			✓
Pengpid and Peltzer^81^	People aged 15+ (NIDS national survey)	2014–15		✓		✓	✓		✓			
Petersen *et al*^82^	Adults aged 18+ attending public clinics, North West province	2014–15				✓	✓	✓	✓			
Sewpaul *et al*^21^	People aged 15+ (SANHANES national survey)	2012					✓		✓	✓	✓	
Sharman and Bachmann^83^	People aged 15+, uMkhanyakude district	2015					✓	✓	✓			✓
van Heerden *et al*^84^	Adults aged 18+, uMgungundlovu district	2015				✓	✓	✓	✓			
Weimann *et al*^52^	People aged 15+ (NIDS national surveys)	2008, 2012					✓	✓	✓			✓
Wong *et al*^53^	People aged 15+, uMkhanyakude district	2018–19					✓	✓	✓			✓
Unpublished	Members of Medscheme medical schemes, ages 15+	2022	✓	✓	✓	✓	✓	✓	✓	✓	✓	
Unpublished	People aged 15+ attending public health facilities, Western Cape province	2022					✓	✓	✓			✓
Unpublished	People aged 15+ (DHS national survey)	2016		✓	✓		✓	✓	✓	✓	✓	✓
Unpublished	People aged 18+ (WHS national survey)	2003	✓	✓		✓	✓			✓		

COPD, chronic obstructive pulmonary disease; DHS, Demographic and Health Survey; IHD, ischaemic heart disease; NIDS, National Income Dynamics Study; SAGE, Study on Global Ageing and Adult Health; SANHANES, South African National Health and Nutrition Examination Survey; SASH, South African Stress and Health; WHS, World Health Survey.

For 38 of the 45 possible condition combinations, there were sufficient data to perform meta-regressions. In 17 of these, there was a significant negative association between the observed OR and the ‘expected prevalence’ under conditions of independence, in line with prior expectation. Full results of the meta-analyses and meta-regressions are summarised in online supplemental file 1, tables S2-45 and figures S1-38.

Estimates of the national prevalence of individual conditions are summarised in table 2. Hypertension is the most highly prevalent condition (33.9%), followed by HIV (18.2%), arthritis (11.3%) and diabetes (10.2%). For all conditions other than HIV and depression, prevalence is higher at ages 50 and older than in the 15–49 age group, with the prevalence of hypertension reaching 70.6% in South African adults aged 50 and older.

**Table 2:** National prevalence estimates for individual conditions

	Ages 15+	Ages 15–49	Ages 50+	Source
Arthritis	11.3% (8.9–13.7)	5.6% (3.7–7.5)	28.4% (20.7–36.2)	WHS 2003^11^*
Asthma	3.5% (3.1–4.0)	2.7% (2.2–3.1)	5.9% (4.9–6.9)	DHS 2016^44^*
COPD	1.8% (1.5–2.2)	1.2% (0.8–1.5)	3.5% (2.7–4.3)	DHS 2016^44^†
Depression	4.9% (3.9–5.9%)	4.9% (3.8–6.1)	4.8% (2.6–6.9)	SASH 2002–4^50^†
Diabetes	10.2% (8.9–11.7)	4.6% (3.6–5.8)	24.7% (21.6–28.1)	SANHANES 2012^21^‡
HIV/AIDS	18.2% (17.5–18.6)	19.2% (18.5–19.7)	15.2% (14.3–15.9)	Thembisa 2021^19^§
Hypertension	33.9% (31.8–36.1)	19.7% (17.7–21.9)	70.6% (66.8–74.1)	SANHANES 2012^21^‡
IHD/angina	5.6% (4.5–6.9)	4.0% (3.0–5.4)	9.7% (7.4–12.5)	SANHANES 2012^21^*
Stroke	2.6% (2.0–3.5)	1.0% (0.7–1.6)	6.7% (4.7–9.4)	SANHANES 2012^21^*
Tuberculosis	0.99% (0.95–1.04)	0.92% (0.88–0.97)	1.21% (1.17–1.25)	Thembisa 2021^49^§¶

*Self-reported past diagnosis by a healthcare worker.

†Self-reported symptoms suggestive of the condition.

‡Laboratory/clinical diagnosis.

§Mathematical model calibrated to laboratory and survey data.

¶Representing the current prevalence of active tuberculosis, including currently treated and untreated individuals.

COPD, chronic obstructive pulmonary disease; DHS, Demographic and Health Survey; IHD, ischaemic heart disease; SANHANES, South African National Health and Nutrition Examination Survey; SASH, South African Stress and Health Survey; WHS, World Health Survey.

**Figure 1:**
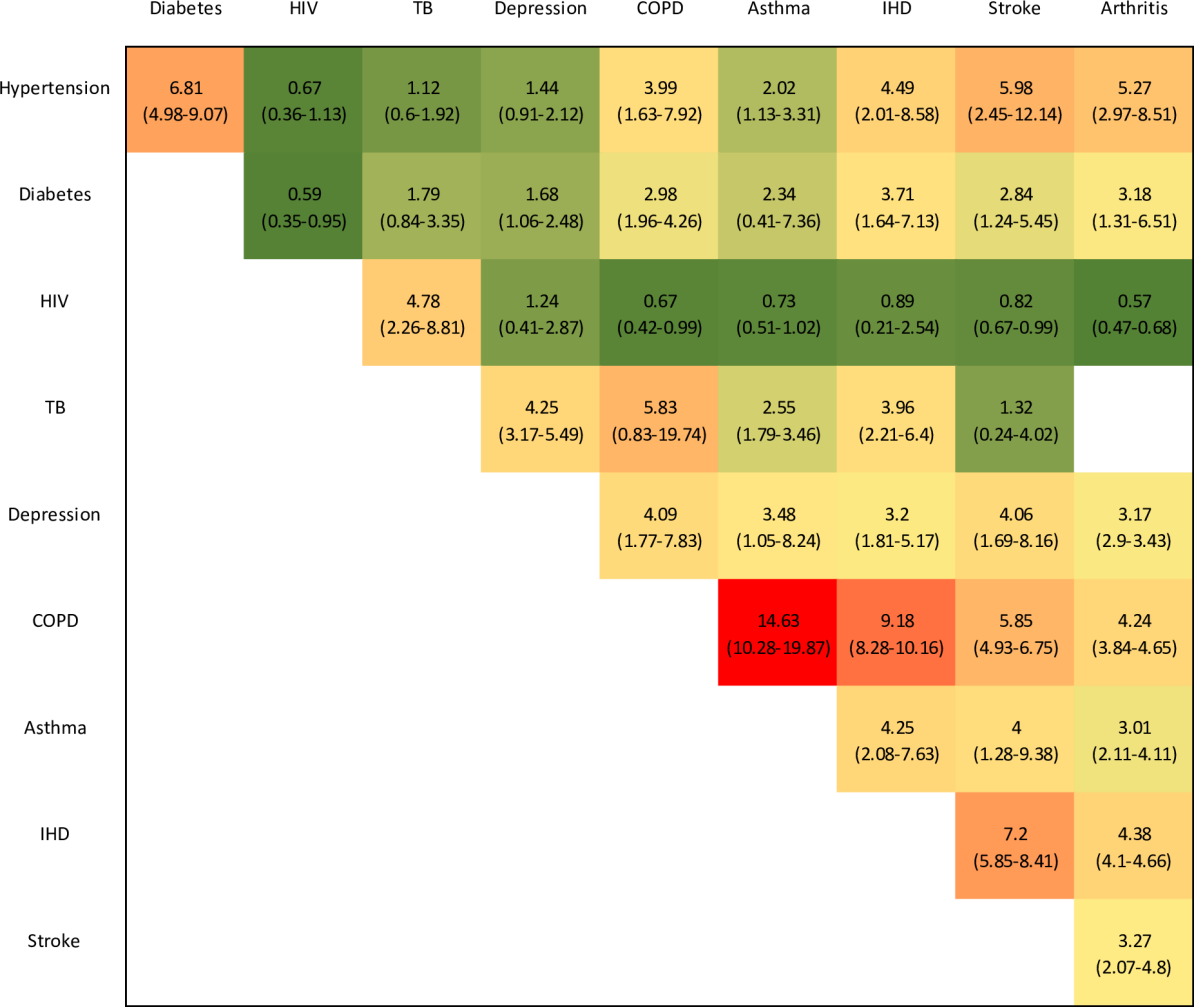
ORs representing strength of association between conditions. ORs range from less than 1 (dark green) to 15 (red). 95% CIs are in brackets. COPD, chronic obstructive pulmonary disease; IHD, ischaemic heart disease; TB, tuberculosis.

Figure 1 summarises the ORs estimated for each condition combination (where the ORs are estimated by meta-regression, the ORs and CIs are estimated using the bootstrapping procedure described previously, that is, standardising to the national prevalence levels in table 2). The strongest positive associations are estimated in the case of COPD and asthma (OR 14.6, 95% CI 10.3 to 19.9), COPD and IHD (OR 9.2, 95% CI 8.3 to 10.2) and IHD and stroke (OR 7.2, 95% CI 5.9 to 8.4). HIV is negatively associated with most other conditions, the negative associations being most significant for HIV and arthritis (OR 0.57, 95% CI 0.47 to 0.68) and HIV and diabetes (OR 0.59, 95% CI 0.35 to 0.95). In the secondary analysis in which ORs were calculated only for ages 50 and older, these were lower than those in figure 1 for almost all condition combinations (online supplemental file 1, figure S39). Controlling for age brought ORs closer to 1 for almost all condition combinations, although most ORs remained significantly different from 1 even after age adjustment (online supplemental file 1, figure S42). Excluding the five studies of patients attending health facilities led to slight increases in ORs for some disease combinations (notably those involving diabetes and hypertension) but slight decreases for others (particularly arthritis) (online supplemental file 1, figure S44).

**Figure 2:**
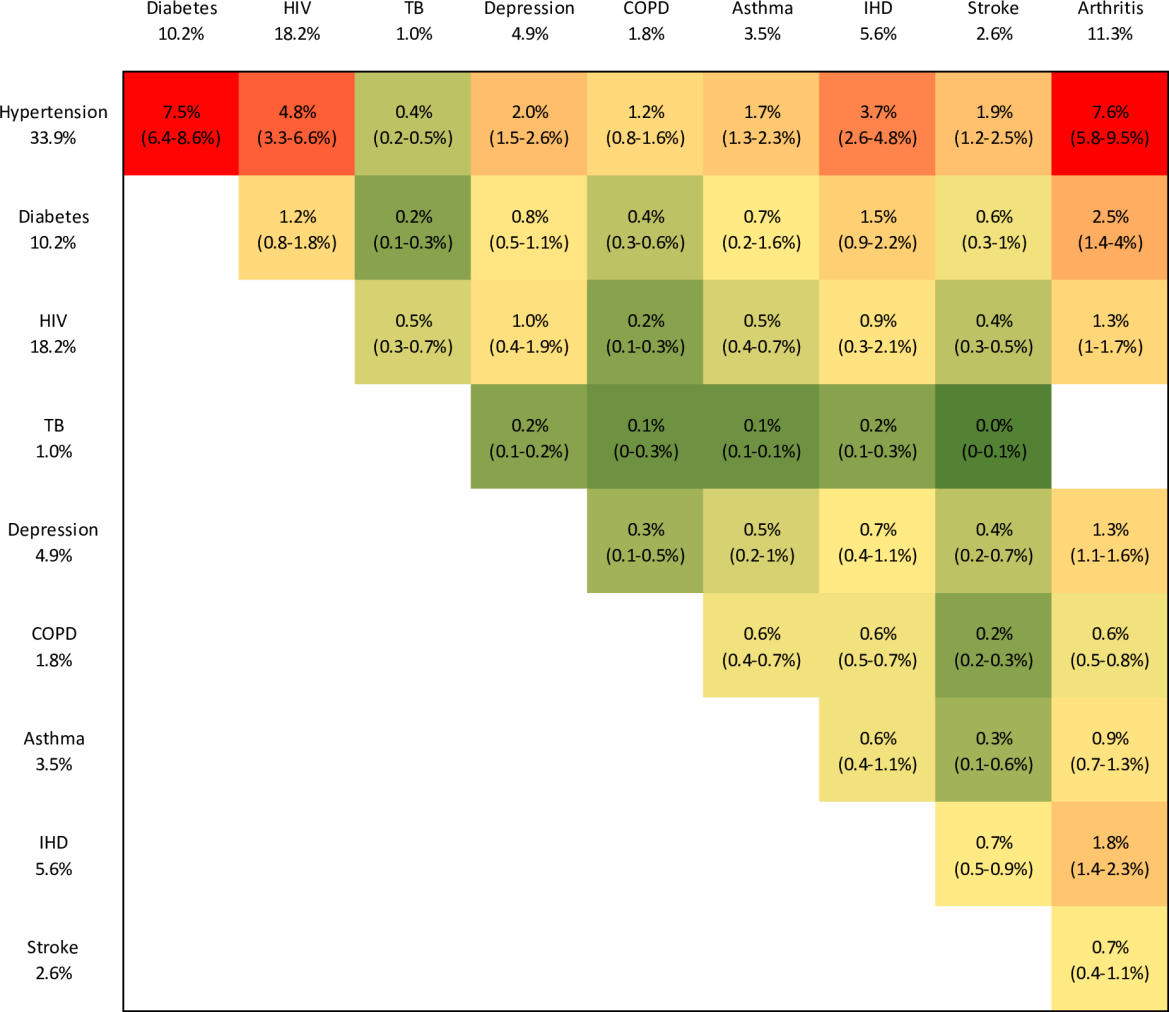
Prevalence of common condition combinations in South Africans aged 15 and older. Prevalence levels range from close to 0% (green) to 8% (red). 95% CIs are in brackets. The prevalence levels for individual conditions (in the row and column headings) are the same as in table 2. COPD, chronic obstructive pulmonary disease; IHD, ischaemic heart disease; TB, tuberculosis.

Figure 2 summarises the prevalence of each pairwise combination. The most highly prevalent condition combinations in individuals aged 15+ are hypertension and arthritis (7.6%, 95% CI 5.8% to 9.5%), hypertension and diabetes (7.5%, 95% CI 6.4% to 8.6%), hypertension and HIV (4.8%, 95% CI 3.3% to 6.6%), hypertension and IHD (3.7%, 95% CI 2.6% to 4.8%) and diabetes and arthritis (2.5%, 95% CI 1.4% to 4.0%). In secondary analyses restricted to the 50+ age groups, estimates of prevalence are consistently higher in the 50+ age group, except in the case of the HIV–depression and TB–depression disease combinations (online supplemental file 1, figure S40). The most highly prevalent disease combinations in older adults are again hypertension and arthritis (22.5%, 95% CI 15.2% to 29.7%), and hypertension and diabetes (21.4%, 95% CI 18.5% to 24.2%). Similar results were obtained when excluding studies in patients attending health facilities (online supplemental file 1, figure S45), although the prevalence of the hypertension–arthritis combination decreased and its uncertainty increased substantially (6.5%, 95% CI 2.1% to 10.5%).

**Figure 3:**
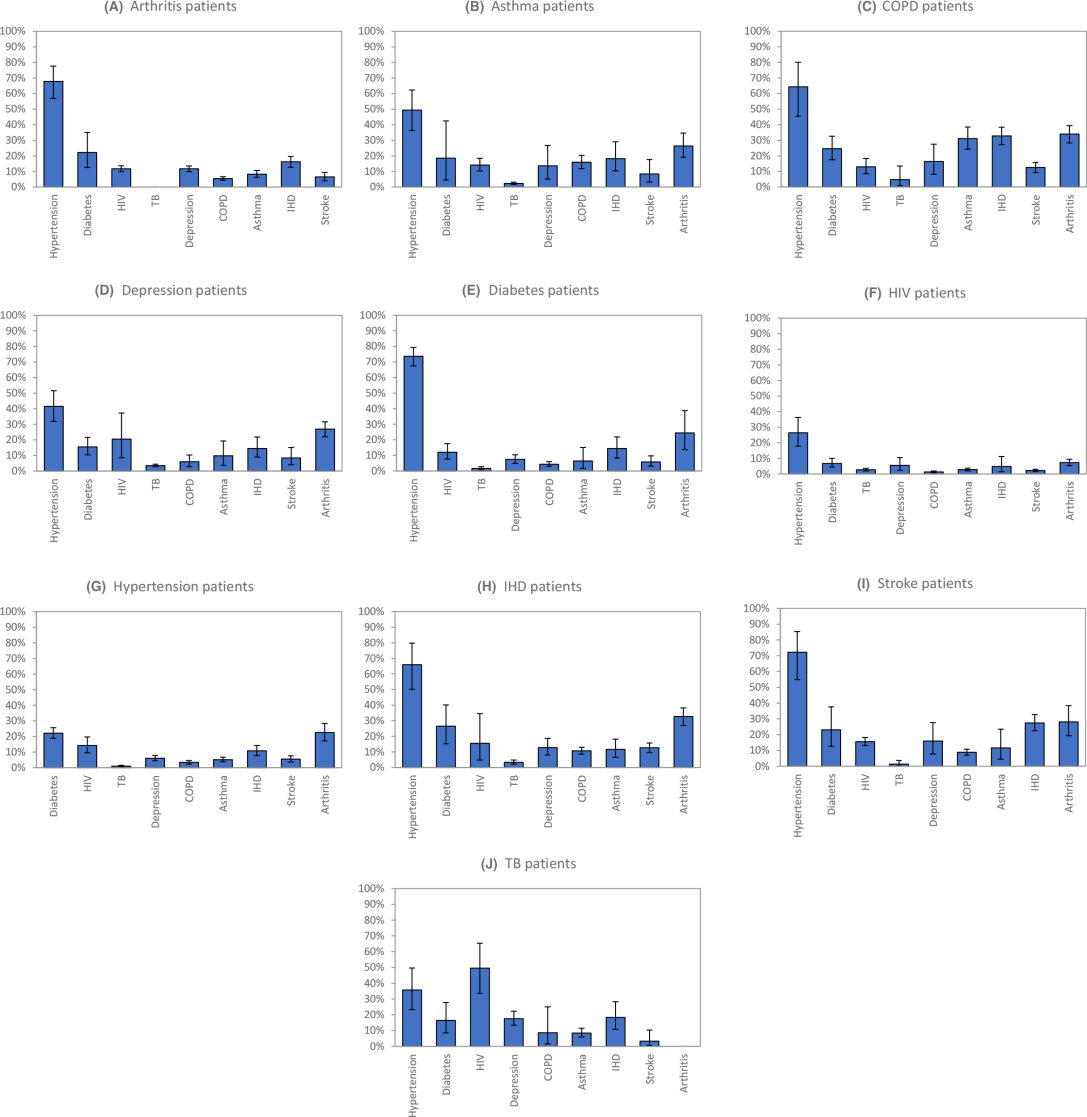
Expected prevalence of different conditions in patients with each index condition. COPD, chronic obstructive pulmonary disease; IHD, ischaemic heart disease; TB, tuberculosis.

Figure 3 represents the expected prevalence of different comorbidities, for patients with each index condition. Hypertension occurs in the majority of patients with NCDs (the prevalence being as high as 74% in diabetes patients) but is less frequent in HIV and TB patients. Diabetes is particularly common in patients with IHD (26%), COPD (25%) and stroke (23%). The expected prevalence of depression is highest in people with TB (17%), COPD (16%) and stroke (16%). HIV prevalence is high in people with TB (50%) but is relatively less common in other patient groups. Arthritis is particularly common in people with COPD (34%) and IHD (33%). IHD is particularly prevalent in people with COPD (33%) and stroke (28%). Asthma is relatively uncommon, except in patients with COPD (31%), and correspondingly, the prevalence of COPD is only substantial in patients with asthma (16%). Stroke is also relatively infrequent, its prevalence being highest in patients with IHD and COPD (13%). TB is the least frequent comorbidity, its prevalence being highest in patients with COPD (5%), depression (4%) and IHD (3%).

**Figure 4:**
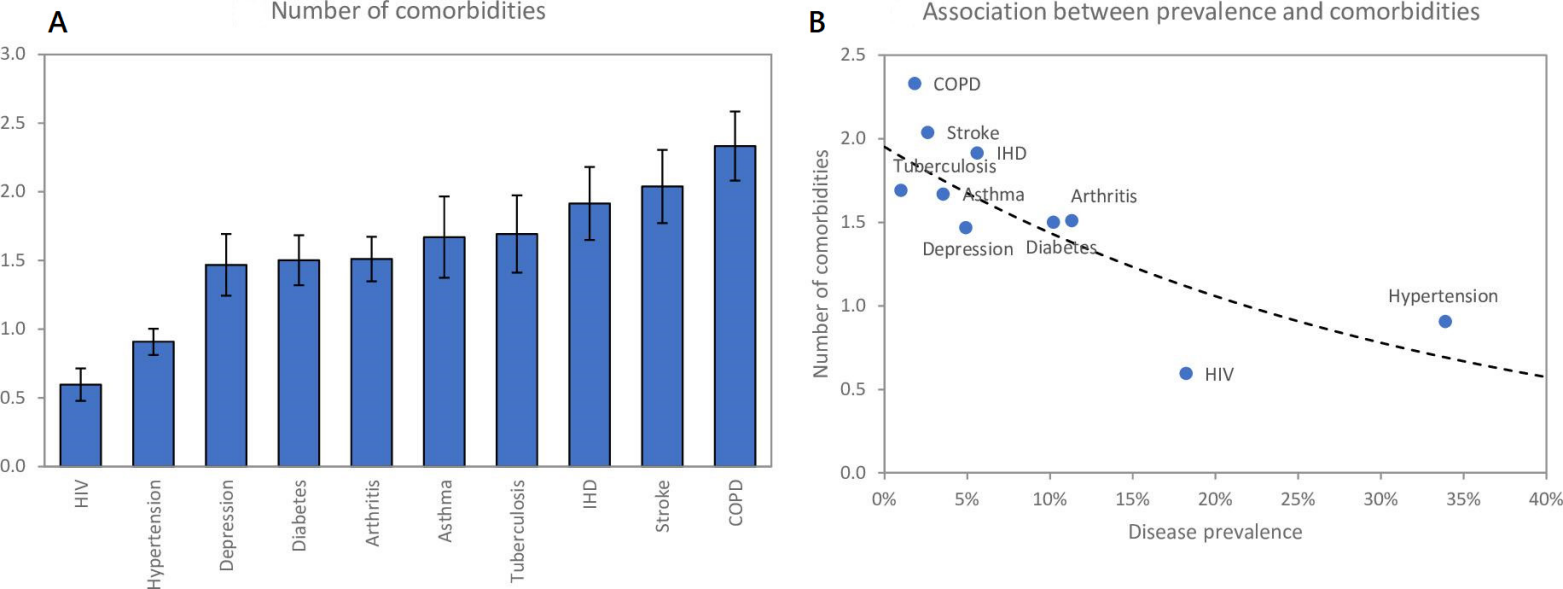
Average number of comorbidities in patients with each index condition. COPD, chronic obstructive pulmonary disease; IHD, ischaemic heart disease.

The average number of comorbidities, for patients with each index condition, is represented in figure 4A. People with COPD have the highest average number of comorbidities (2.3, 95% CI 2.1 to 2.6), followed by stroke (2.1, 95% CI 1.8 to 2.4), IHD (1.9, 95% CI 1.6 to 2.2), TB and asthma (both 1.7, 95% CI 1.4 to 2.0). Relatively low numbers of comorbidities are expected in people living with HIV (0.6, 95% CI 0.5 to 0.7) and hypertension (0.9, 95% CI 0.8 to 1.0). The average number of comorbidities is negatively associated with the prevalence of the index condition (r=−0.78), reducing by a factor of 0.74 (95% CI 0.62 to 0.88) for each 10% increase in prevalence (figure 4B).

## Discussion

By international standards, South Africa has very high levels of HIV, hypertension, diabetes and arthritis, and this is reflected in our estimates of the most frequent disease combinations: hypertension and arthritis, hypertension and diabetes, and hypertension and HIV. However, the strongest associations are between less prevalent conditions (COPD, asthma, IHD and stroke), and there are correspondingly high estimates of numbers of comorbidities in patients with these conditions. These latter conditions, therefore, contribute disproportionately to the multimorbidity burden in South Africa. Our results also show that there is an extremely high prevalence of most condition combinations in older adults (ages 50 and older). We also show that although HIV and TB are strongly associated, HIV is at present largely uncorrelated with the cluster of cardiometabolic and other chronic conditions. The high prevalence of these conditions is widely recognised due to the huge load they place on South African health services, but the high rates of comorbidity are less well recognised, and highlight the need for health services to consider, test for and manage multimorbidity, especially in older adults already receiving long-term care.

Previous studies on multimorbidity in South Africa have focused on characterising the overall prevalence of multimorbidity and the relative frequency of different numbers of conditions, as well as age, sex and socioeconomic differences in the prevalence of multimorbidity.^7 52–54^ There have been relatively few attempts to characterise the specific condition combinations that contribute most to the multimorbidity burden. Although a number of studies have reported the hypertension–diabetes disease combination to be most prevalent in the South African setting,^21 52 55 56^ the hypertension–arthritis combination, which we found to be most prevalent, has not previously been identified as common except in international settings.^8 47^ These findings have important implications for health service design. They suggest a high prevalence of single-condition burdens related to HIV and hypertension, high numbers of comorbidities in people with certain index conditions such as COPD and IHD, and substantial populations of people living with two conditions, some of which are historically recognised (eg, diabetes and hypertension, and TB and HIV^35^) and others less so (eg, hypertension and arthritis). These burdens require more nuanced service design for people living with chronic conditions: convenience-based fast-tracked services for those with single conditions that are well controlled^57^; longer consultations^58^ and greater multidisciplinary involvement^59^ for index conditions that are inherently multimorbid; and review of clinical considerations for combinations where adverse drug effects limit therapeutic options (eg, non-steroidal anti-inflammatory drugs, commonly used to treat arthritis, increase risk of cardiovascular disease^60^).

Our approach is novel, as it integrates evidence from multiple epidemiological data sources, including national surveys, smaller studies in specific districts, routine data from health services (both public and private) and outputs from mathematical models. In addition to achieving greater statistical precision (ie, narrower confidence intervals), this enables us to produce more generalisable estimates for a greater range of conditions, which is important given that no single data source covers all of the conditions in which we are interested. Unlike previous studies that have synthesised multimorbidity data,^1 47 48^ our goal is not to produce an estimate of the prevalence of multimorbidity or to systematically review multimorbidity data, but rather to produce standardised estimates of the prevalence of different condition combinations and levels of comorbidity in patients with different index conditions. Our approach builds on an OR model, which is appropriate for understanding ‘associative multimorbidity’ (ie, understanding which condition combinations are most strongly associated rather than merely most frequent).^14^ Although this method for estimating the joint prevalence of two conditions has previously been proposed,^51^ we are not aware of it having been applied in multimorbidity research.

An important insight is that it is insufficient to combine measures of association between two diseases using simple meta-analysis: in almost all cases, meta-regression modelling suggests that the strength of association is negatively related to the prevalence of the respective conditions. This negative relationship emerges due to differences between studies in the relative size of the ‘healthy’ population without any conditions: the larger the proportion of ‘healthy’ individuals in the sample, the more likely it is that different conditions will appear to cluster together (online supplemental file 2). In the extreme case where there are no ‘healthy’ individuals (eg, in studies of people attending health facilities), measures of association may even become negative. The meta-regression approach thus enables us to combine data from different study types, and to standardise the measure of association for a given expected prevalence of each condition.

Although our study includes a more comprehensive list of conditions than most previous studies of multimorbidity in South Africa, there are a number of conditions that it was not possible to include. Hyperlipidaemia, heart failure, dysrhythmias, chronic kidney disease, hypothyroidism, gastro-oesophageal reflux disease, epilepsy and bipolar disorder were all found to be relatively common in the private sector database but were excluded due to a lack of nationally representative prevalence estimates. Anaemia, although highly prevalent in South Africa,^7 44^ was excluded because it was considered an episodic, curable condition.^61^ Obesity was excluded as it was considered a risk factor rather than a disease. The high prevalence of obesity in South Africa, especially in women,^26^ could be important in explaining why South Africa has relatively high levels of hypertension and diabetes, as well as arthritis.^62^

Our analysis does not consider combinations of three or more diseases. This is a limitation of the OR model. In South Africa, the fraction of multimorbid patients who have three or more conditions has been estimated as being between 15% and 54%^7 54 63^ (although the higher estimates are from studies that include very common conditions that were not included in our analysis, notably anaemia and hyperlipidaemia). There would thus be value in assessing the relative frequency of these combinations of three or more diseases. However, there are challenges in defining appropriate measures of association and graphically representing three-way frequencies/associations, and an analysis of these three-way disease combinations is beyond the scope of this paper.

Another limitation of this modelling approach is that we do not explicitly adjust for differences across studies in methods used to determine the prevalence of different conditions. However, most studies use similar definitions for each condition (online supplemental file 1, table S1.1), and even where there is substantial variation, this heterogeneity would be reflected in the variance around the ORs predicted by the meta-regression model. In defining the national prevalence of certain conditions (table 2), we have relied on self-reported past diagnosis or self-reported symptoms of the condition, which may be inaccurate. However, South African studies of the prevalence of arthritis and asthma find relatively consistent estimates when comparing these two self-reported measures.^44 64^ In the case of IHD, the report of past diagnosis (the measure we have used) yields a slightly higher prevalence than self-report based on symptoms.^65^ It is therefore unlikely that these self-reported measures would substantially understate the true prevalence of the respective conditions.

Our analysis focuses only on crude measures of association between different conditions (ie, unadjusted for likely common risk factors that might explain these associations), as this is a necessary requirement of the statistical model for estimating joint distributions.^51^ It is therefore important not to interpret the ORs as indicating a causal relationship. The NCDs that we have considered are more prevalent at older ages than at younger ages, and age is therefore an obvious explanation for many of the positive associations between NCDs in figure 1. In contrast, HIV is more prevalent in younger adults, and this might explain the negative association seen between HIV and some NCDs, despite international evidence that HIV may increase the risk of cardiovascular disease.^16^ However, in our analysis of the Medscheme data, most disease associations remained significant even when controlling for age (online supplemental file 1, figure S42), indicating that age only partially explains the observed associations. Other risk factors, such as obesity and smoking, are also common to many NCDs and may also be important in explaining the observed associations. Most conditions are more prevalent in women than in men, which might partly explain why conditions appear associated in analyses that are not stratified by sex.

Respiratory conditions are particularly strongly associated with other conditions. TB is strongly positively associated with several NCDs, in line with previous reviews of associations with depression,^46^ diabetes,^66^ cardiovascular disease,^67^ asthma and COPD.^29^ Asthma and COPD are the most strongly associated conditions. In the context of private sector data, this could be partly due to patients registering for both COPD and asthma care in order to access a broader range of drugs (even if they only have one of the conditions). In the context of population-level survey data, the association could be due to survey respondents interpreting asthma and COPD as the same condition, or clinicians misdiagnosing the two conditions. Our estimates for asthma and COPD therefore need to be treated with some caution.

Although South Africa has achieved high levels of treatment coverage for HIV (69% in 2021^19^), treatment coverage for most other chronic conditions is relatively low (eg, 8% for mental illness,^68^ 23% for hypertension^69^ and 38% for diabetes^70^). The HIV-TB cluster has been a major focus of guideline development, policies and health systems strengthening, while other conditions have been less of a priority. For example, 95%-95%-95% targets have been set in the case of HIV (for diagnosis, treatment uptake and control, respectively),^71^ but for diabetes and hypertension the corresponding targets are 90%-60%-50%, and for most other conditions no targets have been set.^72^ Multimorbidity involving NCDs and mental illness has not received the attention it urgently needs.^72^

## Conclusion

This study estimates a high prevalence of various condition combinations in South Africa. The need to screen for comorbidities is greatest in patients with less common conditions (such as COPD, stroke and IHD), who have relatively more comorbidities. However, for the older population, a more holistic regular screening programme might be appropriate, given the high prevalence of multimorbidity in this group. Our estimates inform interventions that focus on enhanced and integrated screening and management of conditions in primary healthcare.

## Data Availability

All data relevant to the study are included in the article or uploaded as online supplemental information. Supplementary data are available online (SupplementaryFile1.pdf includes more detailed results, SupplementaryFile2.pdf explains why ORs may depend on the prevalence of the conditions of interest, and SupplementaryFile3.xlsx contains the study data and key calculations).
