# Supplementary material for: A model-based approach to estimating the prevalence of disease combinations in South Africa
Source: BMJ Glob Health. 2024 Feb 22;9(2):e013376. doi: 10.1136/bmjgh-2023-013376 (PMC10884267; doi:10.1136/bmjgh-2023-013376)
Supplement: Supplementary data [file bmjgh-2023-013376supp001.pdf]

Supplementary materials

Table of Contents

1. Search results ... 3
2. Disease definitions ... 3
3. Results of meta-analyses and meta-regressions ... 6
3.1 Arthritis and asthma ... 7
3.2 Arthritis and COPD ... 8
3.3 Arthritis and depression ... 9
3.4 Arthritis and diabetes ... 10
3.5 Arthritis and HIV ... 11
3.6 Arthritis and hypertension ... 12
3.7 Arthritis and ischaemic heart disease ... 13
3.8 Arthritis and stroke ... 14
3.9 Arthritis and tuberculosis ... 14
3.10 Asthma and COPD ... 15
3.11 Asthma and depression ... 16
3.12 Asthma and diabetes ... 17
3.13 Asthma and HIV ... 18
3.14 Asthma and hypertension ... 19
3.15 Asthma and ischaemic heart disease ... 20
3.16 Asthma and stroke ... 21
3.17 Asthma and tuberculosis ... 22
3.18 COPD and depression ... 23
3.19 COPD and diabetes ... 24
3.20 COPD and HIV ... 25
3.21 COPD and hypertension ... 26
3.22 COPD and ischaemic heart disease ... 27
3.23 COPD and stroke ... 28
3.24 COPD and tuberculosis ... 29
3.25 Depression and diabetes ... 30
3.26 Depression and HIV ... 31
3.27 Depression and hypertension ... 32
3.28 Depression and ischaemic heart disease ... 33

Johnson LF, *et al. BMJ Glob Health* 2024; 9:e013376. doi: 10.1136/bmjgh-2023-013376

3.29 Depression and stroke 34
3.30 Depression and tuberculosis 35
3.31 Diabetes and HIV 36
3.32 Diabetes and hypertension 37
3.33 Diabetes and ischaemic heart disease 38
3.34 Diabetes and stroke 39
3.35 Diabetes and tuberculosis 40
3.36 HIV and hypertension 41
3.37 HIV and ischaemic heart disease 42
3.38 HIV and stroke 43
3.39 HIV and tuberculosis 44
3.40 Hypertension and ischaemic heart disease 45
3.41 Hypertension and stroke 46
3.42 Hypertension and tuberculosis 47
3.43 Ischaemic heart disease and stroke 48
3.44 Ischaemic heart disease and tuberculosis 49
3.45 Stroke and tuberculosis 49
4. Additional analyses 50
4.1 Results for adults aged 50 and older 50
4.2 Results for Medscheme 53
4.3 Results when excluding individuals attending health facilities 57
References 61

1. Search strategy and search results

The search terms multimorbidity and 'South Africa' were used to identify relevant studies. The search was conducted on 24 September 2022, using the PubMed and Web of Science databases. A total of 122 records were identified through PubMed and 121 through Web of Science; after removing duplicates, a merged set of 155 unique records was obtained. A total of 113 records were excluded after reviewing the titles and abstracts, leaving 42 records that appeared potentially relevant after the initial screen. Of these, 30 records were removed after full text screening (in almost all cases because the study did not report the prevalence of individual condition combinations, or because the data were reported in multiple publications). Twelve studies were included in the review, covering 11 datasets (two papers reporting on the same dataset were included because they reported on different condition combinations [1, 2]). A further eight studies [3-10] were identified by scanning reference lists of included studies and other reviews, representing a further six datasets (one study [9] related to a dataset that was already included [11], but reported on different disease combinations, and two studies related to the same dataset but reported on different disease combinations [4, 5]). Thus a total of 20 studies were included, relating to 17 distinct datasets.

2. Included diseases and disease definitions

Table S1.1 summarizes the criteria that were applied in selecting the individual conditions that were included in our analysis. Table S1.2 summarizes the disease definitions applied in the different included studies.

Table S1.1: Criteria for selecting included conditions

Condition	Included in recommended 'core list' [12]	National prevalence estimated	At least 4 SA studies on associations	Included in final list
Cancer	✓		✓	
Coronary heart disease	✓	✓	✓	✓
Stroke	✓	✓	✓	✓
Heart failure	✓			
Diabetes	✓	✓	✓	✓
Dementia	✓			
Depression	✓	✓	✓	✓
Schizophrenia	✓			
Anxiety	✓	✓		
Alcohol use disorders	✓	✓		
Drug use disorders	✓	✓		
Chronic liver disease	✓			
Chronic renal disease	✓		✓	
COPD	✓	✓	✓	✓
Asthma	✓	✓	✓	✓
Visual impairment	✓	✓		
Musculoskeletal impairment due to injury	✓			
Osteoarthritis	✓	✓	✓	✓
Chronic pain	✓	✓		

Gynaecological disorders	✓			
Tuberculosis	✓	✓	✓	✓
Malnutrition	✓	✓		
HIV/AIDS	✓	✓	✓	✓
Hypertension*		✓	✓	✓

* Hypertension is not included in the recommended 'core list' of conditions to be included in multimorbidity research [12]. This reflects the approach adopted by the Global Burden of Disease study, which is to define hypertension as a risk factor rather than a condition that by itself causes morbidity/mortality [13]. We have nevertheless included it because it is highly prevalent in South Africa, and clinical guidelines recommend treatment of people diagnosed with hypertension, regardless of whether they have developed more severe cardiovascular conditions. COPD = chronic obstructive pulmonary disease.

Table S1.2: Disease definitions in different datasets

Disease	Study/dataset	Definition
Arthritis	Garin et al [11], Negin et al [9]	Self-report of past diagnosis OR symptoms in last 12 months
	Lalkhen & Mash [14]	Review of medical record or current visit due to condition
	Medscheme	Any past registration for chronic medicine or chronic care
	WHS 2003	Self-report of past diagnosis
Asthma	Ehrlich et al [4]	Self-report of past diagnosis
	Garin et al [11], Negin et al [9]	Self-report of past diagnosis OR symptoms in last 12 months
	Jithoo [7]	Self-report of past diagnosis
	Lalkhen & Mash [14]	Review of medical record or current visit due to condition
	Medscheme	Any past registration for chronic medicine or chronic care
	DHS 2016	Self-report of past diagnosis
	WHS 2003	Self-report of past diagnosis
COPD	Chang et al [1]	Self-reported symptoms of chronic bronchitis (cough with phlegm for ≥3 months)
	Ehrlich et al [5]	Self-reported symptoms of chronic bronchitis (cough with phlegm for ≥3 months)
	Folb et al [15]	Self-reported symptoms of chronic respiratory disease or self-reported history of relevant medication (excluding TB)
	Garin et al [11], Negin et al [9]	Self-report of past diagnosis (COPD/bronchitis/emphysema) OR symptoms in last 12 months
	Jithoo [7]	GOLD stage 1 or higher (based on spirometry)
	Lalkhen & Mash [14]	Review of medical record or current visit due to condition
	Medscheme	Any past registration for chronic medicine or chronic care
	DHS 2016	Self-report of past diagnosis (COPD, chronic bronchitis or emphysema)
Depression	Chang et al [1]	≥3 symptoms on CESD 8-item scale (recent depression)
	Folb et al [15]	≥10 score on CESD 10-item scale (recent depression)
	Garin et al [11], Negin et al [9]	Self-report of past diagnosis OR symptoms of depression in last 12 months (DSM-IV criteria)
	Grimsrud et al [6]	Symptoms of depression in last 12 months (DSM-IV criteria)
	Petersen et al [16]	≥10 score on PHQ-9 scale (recent depression)
	van Heerden et al [17]	≥10 score on PHQ-9 scale (recent depression)
	Medscheme	Any registration for chronic medicine/care or ICD-10 codes in the last 12 months
	WHS 2003	Self-report of past diagnosis
Diabetes	Chang et al [1]	Fasting glucose ≥126 mg/dL, non-fasting glucose ≥200 mg/dL, or self-report of diabetes medication
	Folb et al [15]	Self-reported history of diabetes medication
	Garin et al [11], Negin et al [9]	Self-report of past diagnosis
	Lalkhen & Mash [14]	Review of medical record or current visit due to condition
	Oni et al [18]	Receiving diabetes medication in the last 9 months
	Petersen et al [16]	Currently receiving treatment for diabetes
	Sewpaul et al [19]	HbA1c ≥6.5% or self-report of current diabetes medication
	Sharman & Bachmann [20]	Self-report of past diagnosis/treatment

	van Heerden et al [17]	Non-fasting glucose concentration ≥11.0 mmol/l
	Weimann et al [21]	Self-report of past diagnosis
	Wong et al [22]	HbA1c ≥6.5% or self-report of recent diabetes treatment
	Medscheme	Any past registration for chronic medicine or chronic care
	WC PHDC	Dispensed treatment for diabetes, laboratory evidence, or ICD-10 code indicating diabetes
	DHS 2016	HbA1c, adjusted for bias in dried blood spot
	WHS 2003	Self-report of past diagnosis
HIV	Chang et al [1]	Positive HIV ELISA or self-report of past diagnosis
	Corbett et al [3]	Positive HIV test on urine
	Garin et al [11], Negin et al [9]	Positive HIV ELISA
	Middelkoop et al [8]	Positive HIV test on oral transudate
	Oni et al [18]	Receiving HIV treatment in the last 9 months
	Petersen et al [16]	Currently receiving treatment for HIV
	Sharman & Bachmann [20]	Positive HIV ELISA
	van Heerden et al [17]	Positive point-of-care HIV test
	Weimann et al [21]	Self-report of past diagnosis
	Wong et al [22]	Positive HIV ELISA
	Medscheme	Any past registration for chronic medicine or chronic care
	WC PHDC	Evidence of HIV diagnosis, HIV viral load tests, ART, or registration in HIV disease management system
	DHS 2016	HIV ELISA
Hyper-tension	Chang et al [1]	Systolic BP ≥140 mmHg or diastolic BP ≥90 mmHg, or self-report of current hypertensive medication
	Folb et al [15]	Self-reported history of hypertension medication
	Garin et al [11], Negin et al [9]	Systolic BP ≥140 mmHg or diastolic BP ≥90 mmHg, or self-report of past diagnosis
	Grimsrud et al [6]	Self-report of past diagnosis
	Lalkhen & Mash [14]	Review of medical record or current visit due to condition
	Oni et al [18]	Receiving hypertension medication in the last 9 months
	Petersen et al [16]	Currently receiving treatment for hypertension
	Sewpaul et al [19]	Systolic BP ≥140 mmHg or diastolic BP ≥90 mmHg, or self-report of current hypertensive medication
	Sharman & Bachmann [20]	Self-report of past diagnosis/treatment
	van Heerden et al [17]	Systolic BP ≥140 mmHg or diastolic BP ≥90 mmHg
	Weimann et al [21]	Systolic BP ≥140 mmHg or diastolic BP ≥90 mmHg, or self-report of past diagnosis
	Wong et al [22]	Systolic BP ≥140 mmHg or diastolic BP ≥90 mmHg, or recently treated
	Medscheme	Any past registration for chronic medicine or chronic care
	WC PHDC	Dispensed hypertension drugs, or ICD10 code
	DHS 2016	Systolic BP ≥140 mmHg or diastolic BP ≥90 mmHg, or self-report of current hypertensive medication
IHD/ angina	Chang et al [1]	Self-report of symptoms of angina (Rose questionnaire)
	Garin et al [11], Negin et al [9]	Self-report of past angina diagnosis OR symptoms of angina (Rose questionnaire)
	Sewpaul et al [19]	Self-report of past diagnosis (heart attack/angina)
	Medscheme	Any past registration for chronic medicine or chronic care
	DHS 2016	Self-report of past diagnosis (heart attack)
	WHS 2003	Self-report of past diagnosis
Stroke	Garin et al [11], Negin et al [9]	Self-report of past diagnosis OR past symptoms
	Lalkhen & Mash [14]	Review of medical record or current visit due to condition
	Pengpid & Peltzer [2]	Self-report of past diagnosis
	Sewpaul et al [19]	Self-report of past diagnosis
	Medscheme	Any past registration for chronic medicine or chronic care
	DHS 2016	Self-report of past diagnosis
Tubercu-losis	Corbett et al [3]	Sputum positive (microscopy/culture) or chest radiography
	Ehrlich et al [4, 5]	Self-report of past diagnosis
	Jithoo [7]	Self-report of past diagnosis
	Middelkoop et al [8]	Sputum positive (microscopy/culture) or currently treated

Oni et al [18]	Receiving TB treatment in the last 9 months
Sharman & Bachmann [20]	Self-report of diagnosis/treatment in last 12 months
Weimann et al [21]	Self-report of past diagnosis
Wong et al [22]	Sputum positive (Xpert Ultra/culture) or on treatment
WC PHDC	Laboratory evidence of TB diagnosis, registration on TB treatment register, or combination TB treatment
DHS 2016	Self-report of TB diagnosis in the last year

ART = antiretroviral treatment, BP = blood pressure, CES-D: Centre for Epidemiological Studies-Depression, COPD = chronic obstructive pulmonary disease, DHS = Demographic and Health Survey, DSM-IV = Diagnostic and Statistical Manual for Mental Disorders 4th edition, ELISA = enzyme-linked immunosorbent assay, IHD = ischaemic heart disease, PHQ-9 = Patient Health Questionnaire-9, WC PHDC = Western Cape Provincial Health Data Centre, WHS = World Health Survey.

3. Results of meta-analyses and meta-regressions

In the tables that follow, the ‘n’ column shows the numbers of individuals in whom the prevalence of both conditions could be determined (individuals with missing data on one or both conditions were excluded, and the ‘n’ values can therefore differ across tables for the same study). In the figures that follow, the size of the circles represents the amount of weight given to different odds ratio estimates, which in most cases is a reflection of the sample size. The ‘Expected prevalence’ on the x axis of each figure (and in the tables) is the proportion of the population that would be expected to have both conditions if the two conditions were not associated (i.e. if they occurred independently).

3.1 Arthritis and asthma

Table S2.1: Studies of association between arthritis and asthma

Study	n	Asthma prevalence	Arthritis prevalence	% with both	OR	SE of log(OR)	Expected % with both
Garin 2016	3836	7.7%	30.6%	3.6%	2.18	0.122	2.4%
Lalkhen 2015	18856	2.6%	2.8%	0.1%	2.18	0.200	0.1%
Medscheme	1274501	5.5%	1.9%	0.3%	3.46	0.018	0.1%
WHS	2277	6.7%	10.0%	1.7%	3.41	0.202	0.7%

OR = odds ratio (for the association between the two conditions), SE = standard error.

Table S2.2: Meta-regression results: predictors of log odds ratio

	Mean	Standard error
Expected prevalence term	-13.81	13.6
Constant	1.14	0.17

The variance of the random effects in the meta-regression model, on a log scale, is 0.045.

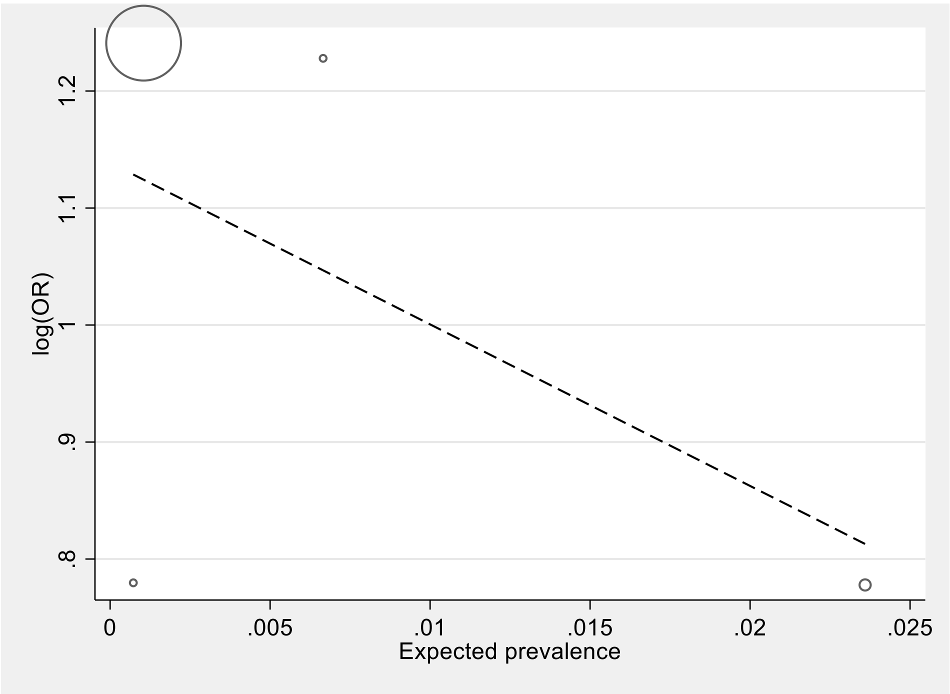

Figure S1: Relationship between expected prevalence (% with both conditions) and log odds ratio

3.2 Arthritis and COPD

Table S3.1: Studies of association between arthritis and COPD

Study	n	COPD prevalence	Arthritis prevalence	% with both	OR	SE of log(OR)	Expected % with both
Garin 2016	3836	4.3%	30.6%	2.14%	2.38	0.161	1.30%
Lalkhen 2015	18856	0.7%	2.8%	0.09%	4.90	0.263	0.02%
Medscheme	1274501	0.8%	1.9%	0.06%	4.68	0.037	0.01%

OR = odds ratio (for the association between the two conditions), SE = standard error.

Table S3.2: Meta-regression results: predictors of log odds ratio

	Mean	Standard error
Expected prevalence term	-52.67	12.81
Constant	1.55	0.04

The variance of the random effects in the meta-regression model, on a log scale, is 0 (i.e. there is no significant heterogeneity across studies when including the expected prevalence term).

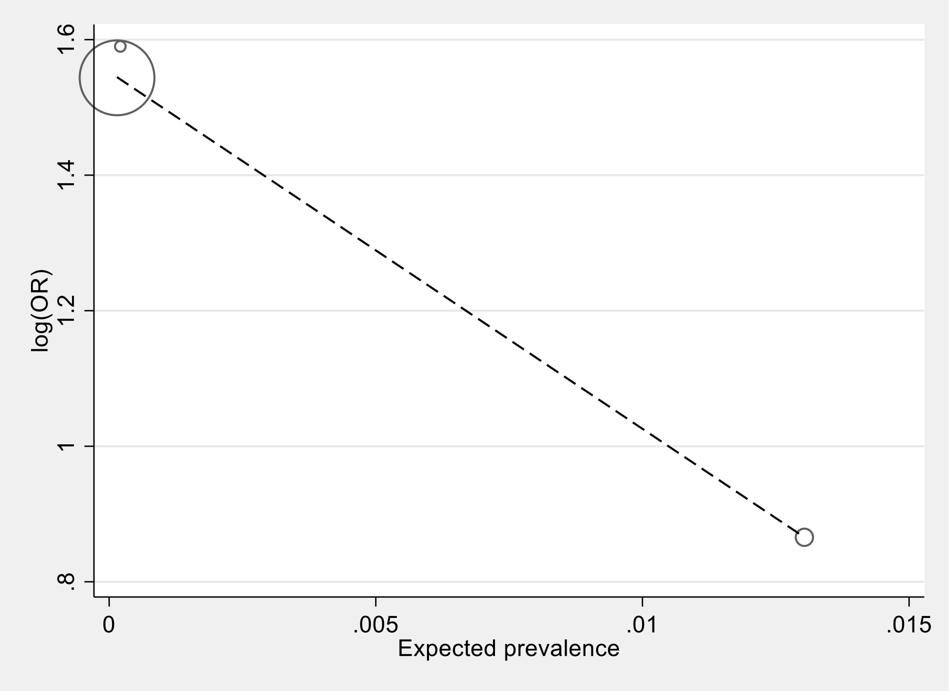

Figure S2: Relationship between expected prevalence (% with both conditions) and log odds ratio

3.3 Arthritis and depression

Table S4.1: Studies of association between arthritis and depression

Study	n	Depression prevalence	Arthritis prevalence	% with both	OR	SE of log(OR)	Expected % with both
Garin 2016	3836	4.8%	30.6%	2.4%	2.48	0.152	1.5%
Medscheme	1274501	6.5%	1.9%	0.4%	3.47	0.017	0.1%
WHS	2254	9.2%	9.9%	2.2%	3.32	0.182	0.9%

OR = odds ratio (for the association between the two conditions), SE = standard error.

Table S4.2: Meta-regression results: predictors of log odds ratio

	Mean	Standard error
Expected prevalence term	-21.33	10.27
Constant	1.27	0.02

The variance of the random effects in the meta-regression model, on a log scale, is 0 (i.e. there is no significant heterogeneity across studies when including the expected prevalence term).

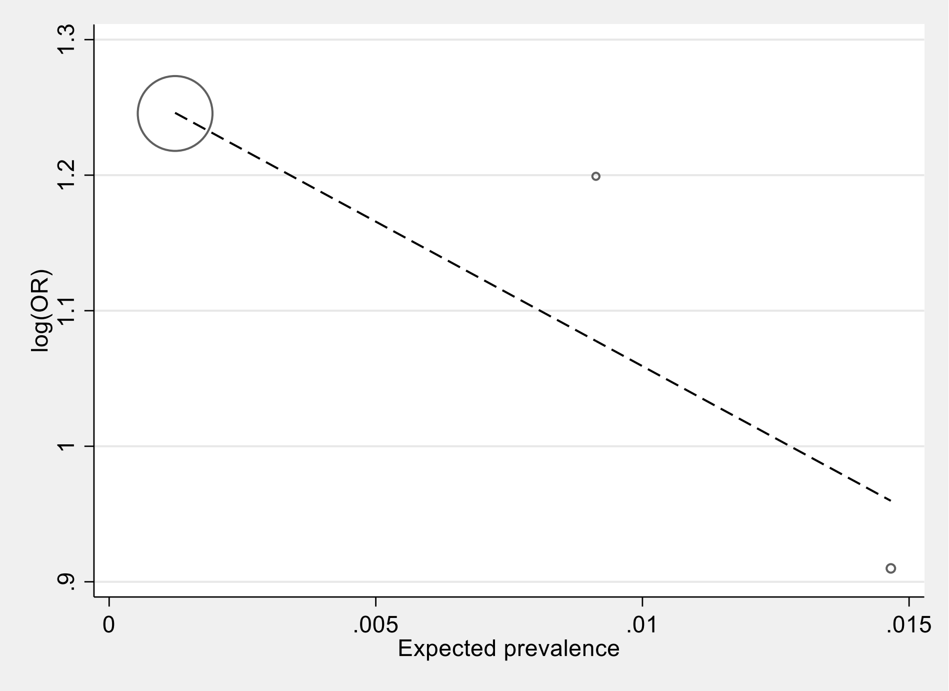

Figure S3: Relationship between expected prevalence (% with both conditions) and log odds ratio

3.4 Arthritis and diabetes

Table S5.1: Studies of association between arthritis and diabetes

Study	n	Diabetes prevalence	Arthritis prevalence	% with both	OR	SE of log(OR)	Expected % with both
Garin 2016	3836	9.2%	30.6%	4.6%	2.54	0.113	2.8%
Lalkhen 2015	18856	5.0%	2.8%	0.2%	1.61	0.166	0.1%
Medscheme	1274501	10.7%	1.9%	0.6%	4.15	0.014	0.2%
WHS	2259	8.6%	10.0%	2.4%	4.19	0.179	0.9%

OR = odds ratio (for the association between the two conditions), SE = standard error.

Table S5.2: Meta-regression results: predictors of log odds ratio

	Mean	Standard error
Expected prevalence term	-2.17	25.38
Constant	1.09	0.38

The variance of the random effects in the meta-regression model, on a log scale, is 0.286.

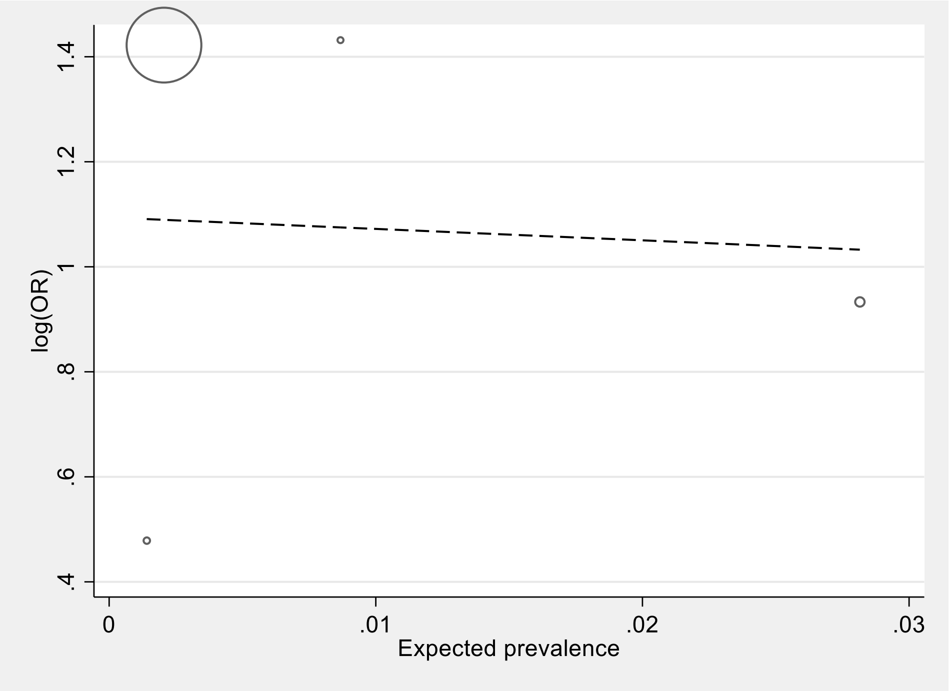

Figure S4: Relationship between expected prevalence (% with both conditions) and log odds ratio

3.5 Arthritis and HIV

Table S6.1: Studies of association between arthritis and HIV

Study	n	HIV prevalence	Arthritis prevalence	% with both	OR	SE of log(OR)	Expected % with both
Negin 2012	2902	6.3%	17.5%	0.55%	0.44	0.266	1.10%
Medscheme	1274501	7.0%	1.9%	0.08%	0.59	0.032	0.13%

OR = odds ratio (for the association between the two conditions), SE = standard error.

Table S6.2: Meta-analysis results: average log odds ratio

	Mean	Standard error
Constant	-0.57	0.09

The variance of the random effects in the meta-regression model, on a log scale, is 0.009.

Johnson LF, *et al. BMJ Glob Health* 2024; 9:e013376. doi: 10.1136/bmjgh-2023-013376

3.6 Arthritis and hypertension

Table S7.1: Studies of association between arthritis and hypertension

Study	n	Hypertension prevalence	Arthritis prevalence	% with both	OR	SE of log(OR)	Expected % with both
Garin 2016	3836	78.3%	30.6%	24.5%	1.17	0.087	24.0%
Lalkhen 2015	18856	17.1%	2.8%	1.4%	4.84	0.089	0.5%
Medscheme	1274501	23.8%	1.9%	1.4%	8.48	0.014	0.5%

OR = odds ratio (for the association between the two conditions), SE = standard error.

Table S7.2: Meta-regression results: predictors of log odds ratio

	Mean	Standard error
Expected prevalence term	-7.25	2.07
Constant	1.9	0.28

The variance of the random effects in the meta-regression model, on a log scale, is 0.132.

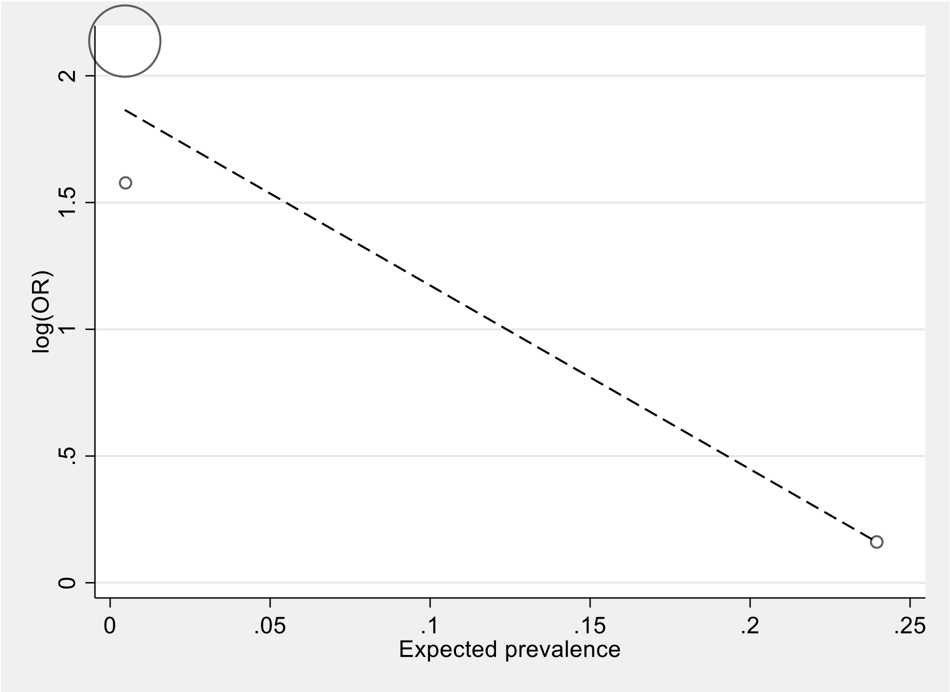

Figure S5: Relationship between expected prevalence (% with both conditions) and log odds ratio

3.7 Arthritis and ischaemic heart disease

Table S8.1: Studies of association between arthritis and ischaemic heart disease (IHD)

Study	n	IHD prevalence	Arthritis prevalence	% with both	OR	SE of log(OR)	Expected % with both
Garin 2016	3836	8.9%	30.6%	5.2%	3.69	0.116	2.7%
Medscheme	1274501	2.9%	1.9%	0.2%	4.59	0.021	0.1%
WHS	2259	5.0%	10.0%	1.4%	3.73	0.223	0.5%

OR = odds ratio (for the association between the two conditions), SE = standard error.

Table S8.2: Meta-regression results: predictors of log odds ratio

	Mean	Standard error
Expected prevalence term	-8.46	4.41
Constant	1.53	0.02

The variance of the random effects in the meta-regression model, on a log scale, is 0 (i.e. there is no significant heterogeneity across studies when including the expected prevalence term).

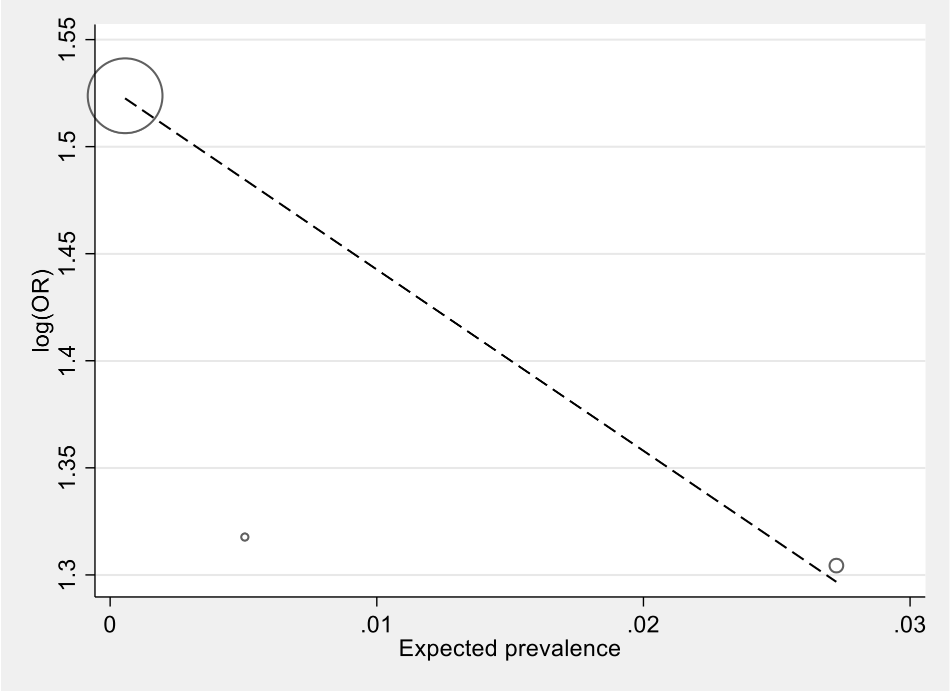

Figure S6: Relationship between expected prevalence (% with both conditions) and log odds ratio

13

3.8 Arthritis and stroke

Table S9.1: Studies of association between arthritis and stroke

Study	n	Stroke prevalence	Arthritis prevalence	% with both	OR	SE of log(OR)	Expected % with both
Garin 2016	3836	4.9%	30.6%	2.50%	2.48	0.150	1.50%
Medscheme	1274501	0.6%	1.9%	0.04%	3.96	0.045	0.01%

OR = odds ratio (for the association between the two conditions), SE = standard error.

Table S9.2: Meta-analysis results: average log odds ratio

	Mean	Standard error
Constant	1.16	0.21

The variance of the random effects in the meta-regression model, on a log scale, is 0.097.

3.9 Arthritis and tuberculosis

No local studies on the association between arthritis and tuberculosis were found.

3.10 Asthma and COPD

Table S10.1: Studies of association between asthma and COPD

Study	n	COPD prevalence	Asthma prevalence	% with both	OR	SE of log(OR)	Expected % with both
DHS 2016	10288	1.3%	3.6%	0.36%	11.49	0.202	0.05%
Garin 2016	3836	4.3%	7.7%	2.23%	18.00	0.172	0.33%
Medscheme	1274501	0.8%	5.5%	0.33%	13.62	0.021	0.04%
Jithoo 2006	847	23.1%	14.5%	5.90%	2.71	0.206	3.36%

OR = odds ratio (for the association between the two conditions), SE = standard error.

Table S10.2: Meta-regression results: predictors of log odds ratio

	Mean	Standard error
Expected prevalence term	-49.73	11.18
Constant	2.70	0.17

The variance of the random effects in the meta-regression model, on a log scale, is 0.052.

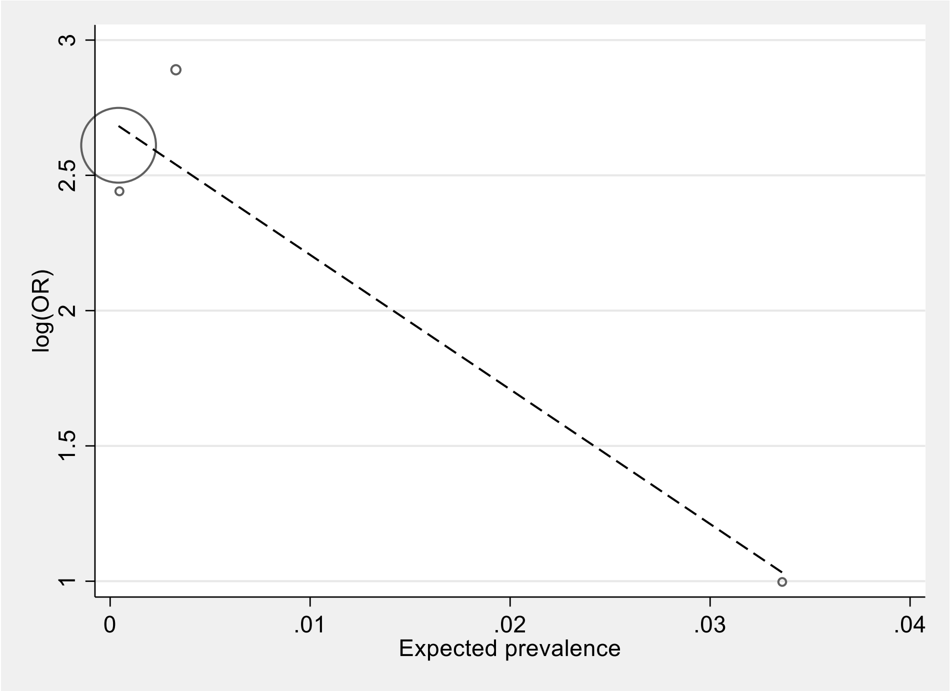

Figure S7: Relationship between expected prevalence (% with both conditions) and log odds ratio

3.11 Asthma and depression

Table S11.1: Studies of association between asthma and depression

Study	n	Depression prevalence	Asthma prevalence	% with both	OR	SE of log(OR)	Expected % with both
Garin 2016	3836	4.8%	7.7%	1.54%	6.82	0.172	0.37%
Medscheme	1274501	6.5%	5.5%	0.75%	2.49	0.012	0.35%
WHS	2252	9.1%	6.7%	1.64%	3.75	0.206	0.61%
Pengpid 2018	22633	13.0%	2.2%	0.39%	1.47	0.119	0.28%

OR = odds ratio (for the association between the two conditions), SE = standard error.

Table S11.2: Meta-regression results: predictors of log odds ratio

	Mean	Standard error
Expected prevalence term	191.7	295.8
Constant	0.36	1.24

The variance of the random effects in the meta-regression model, on a log scale, is 0.216.

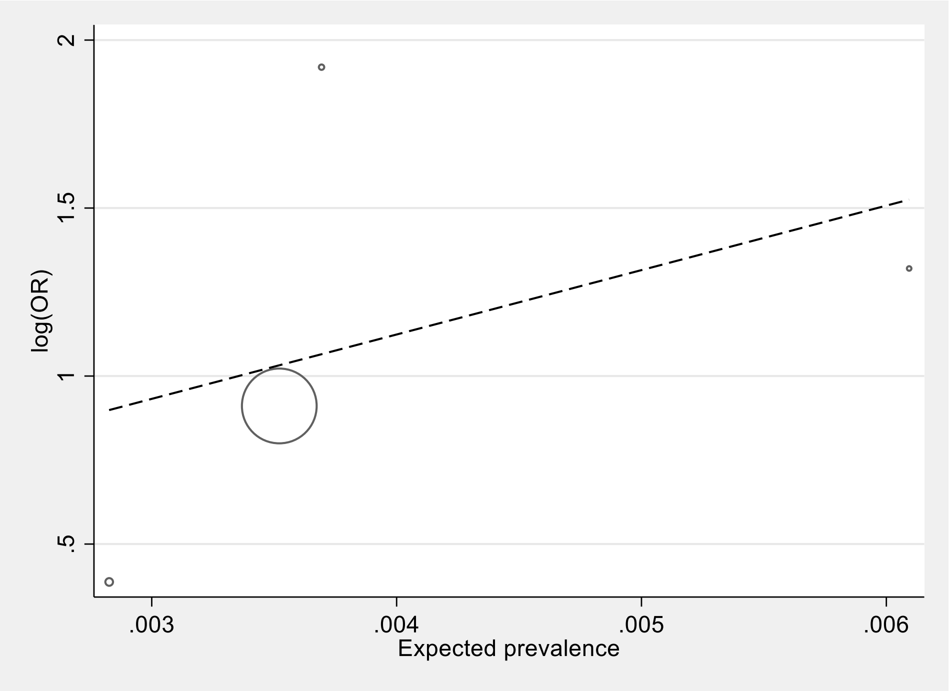

Figure S8: Relationship between expected prevalence (% with both conditions) and log odds ratio

3.12 Asthma and diabetes

Table S12.1: Studies of association between asthma and diabetes

Study	n	Diabetes prevalence	Asthma prevalence	% with both	OR	SE of log(OR)	Expected % with both
DHS 2016	6730	11.9%	3.9%	0.71%	1.71	0.164	0.46%
Garin 2016	3836	9.2%	7.7%	1.30%	2.16	0.167	0.71%
Medscheme	1274501	10.7%	5.5%	1.21%	2.54	0.010	0.59%
WHS	2261	8.6%	6.6%	1.06%	2.19	0.237	0.57%

OR = odds ratio (for the association between the two conditions), SE = standard error.

Table S12.2: Meta-regression results: predictors of log odds ratio

	Mean	Standard error
Expected prevalence term	96.41	130.3
Constant	0.23	0.77

The variance of the random effects in the meta-regression model, on a log scale, is 0.025.

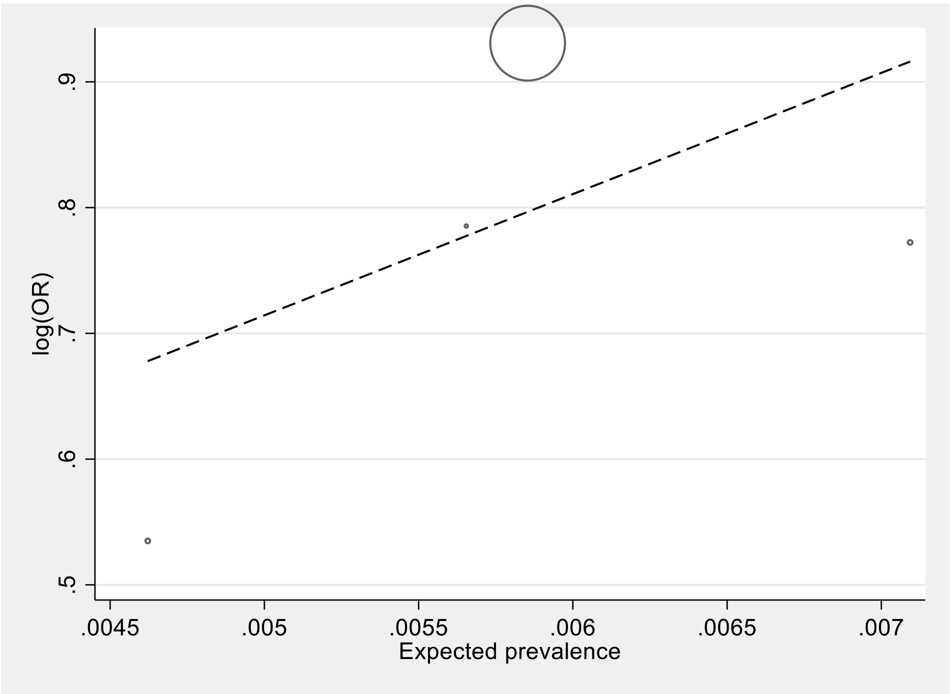

Figure S9: Relationship between expected prevalence (% with both conditions) and log odds ratio

3.13 Asthma and HIV

Table S13.1: Studies of association between asthma and HIV

Study	n	HIV prevalence	Asthma prevalence	% with both	OR	SE of log(OR)	Expected % with both
DHS 2016	6531	19.3%	3.9%	0.57%	0.70	0.181	0.75%
Negin 2012	3049	6.3%	5.6%	0.36%	1.02	0.321	0.36%
Medscheme	1274501	7.0%	5.5%	0.30%	0.78	0.017	0.38%

OR = odds ratio (for the association between the two conditions), SE = standard error.

Table S13.2: Meta-regression results: predictors of log odds ratio

	Mean	Standard error
Expected prevalence term	-29.12	48.81
Constant	-0.14	0.19

The variance of the random effects in the meta-regression model, on a log scale, is 0 (i.e. there is no significant heterogeneity across studies when including the expected prevalence term).

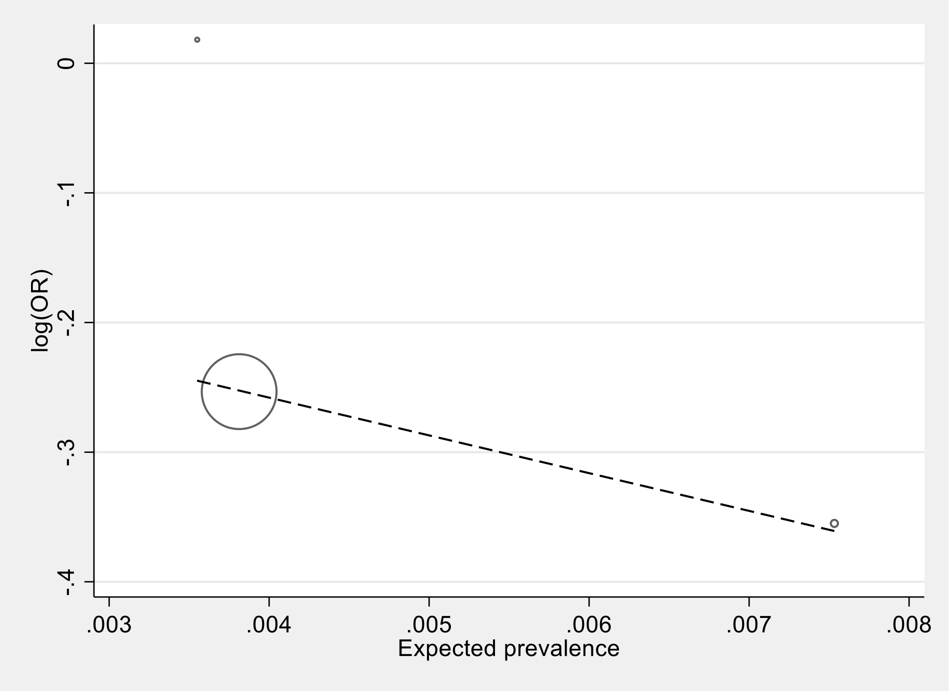

Figure S10: Relationship between expected prevalence (% with both conditions) and log odds ratio

3.14 Asthma and hypertension

Table S14.1: Studies of association between asthma and hypertension

Study	n	Hypertension prevalence	Asthma prevalence	% with both	OR	SE of log(OR)	Expected % with both
DHS 2016	8235	44.7%	3.7%	2.13%	1.70	0.118	1.66%
Garin 2016	3836	78.3%	7.7%	6.25%	1.21	0.154	6.04%
Lalkhen 2015	18856	17.1%	2.6%	0.62%	1.55	0.108	0.44%
Medscheme	1274501	23.8%	5.5%	2.49%	2.89	0.008	1.30%

OR = odds ratio (for the association between the two conditions), SE = standard error.

Table S14.2: Meta-regression results: predictors of log odds ratio

	Mean	Standard error
Expected prevalence term	-8.82	8.95
Constant	0.77	0.28

The variance of the random effects in the meta-regression model, on a log scale, is 0.131.

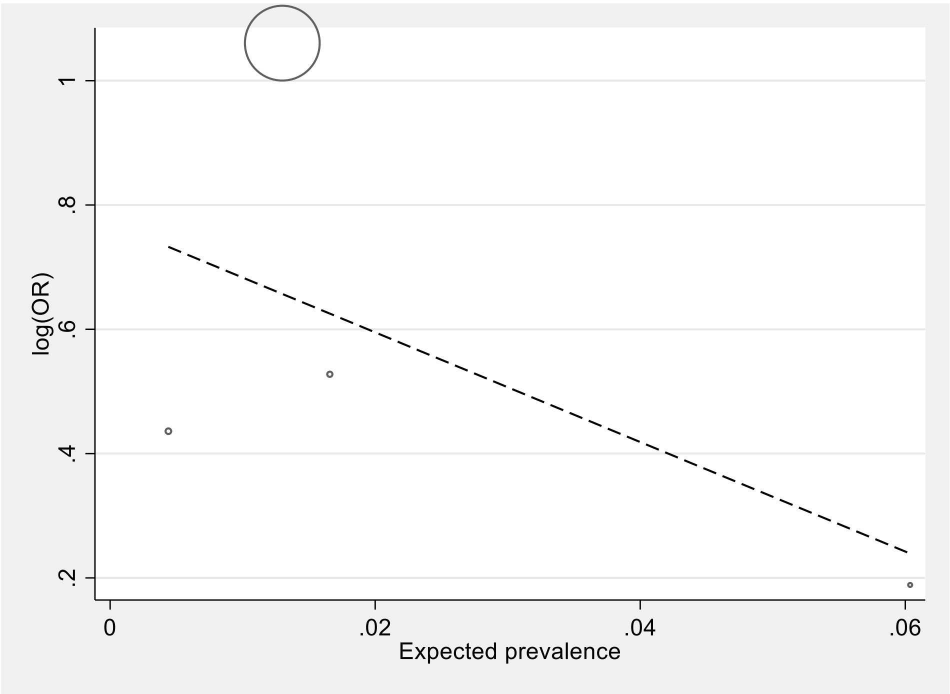

Figure S11: Relationship between expected prevalence (% with both conditions) and log odds ratio

3.15 Asthma and ischaemic heart disease

Table S15.1: Studies of association between asthma and ischaemic heart disease (IHD)

Study	n	Asthma prevalence	IHD prevalence	% with both	OR	SE of log(OR)	Expected % with both
DHS 2016	10294	3.6%	3.4%	0.55%	5.88	0.156	0.12%
Garin 2016	3836	7.7%	8.9%	1.94%	4.13	0.148	0.69%
Medscheme	1274501	5.5%	2.9%	0.41%	3.02	0.015	0.16%
WHS	2258	6.7%	5.1%	0.93%	3.43	0.258	0.34%

OR = odds ratio (for the association between the two conditions), SE = standard error.

Table S15.2: Meta-regression results: predictors of log odds ratio

	Mean	Standard error
Expected prevalence term	-6.02	83.28
Constant	1.40	0.33

The variance of the random effects in the meta-regression model, on a log scale, is 0.119.

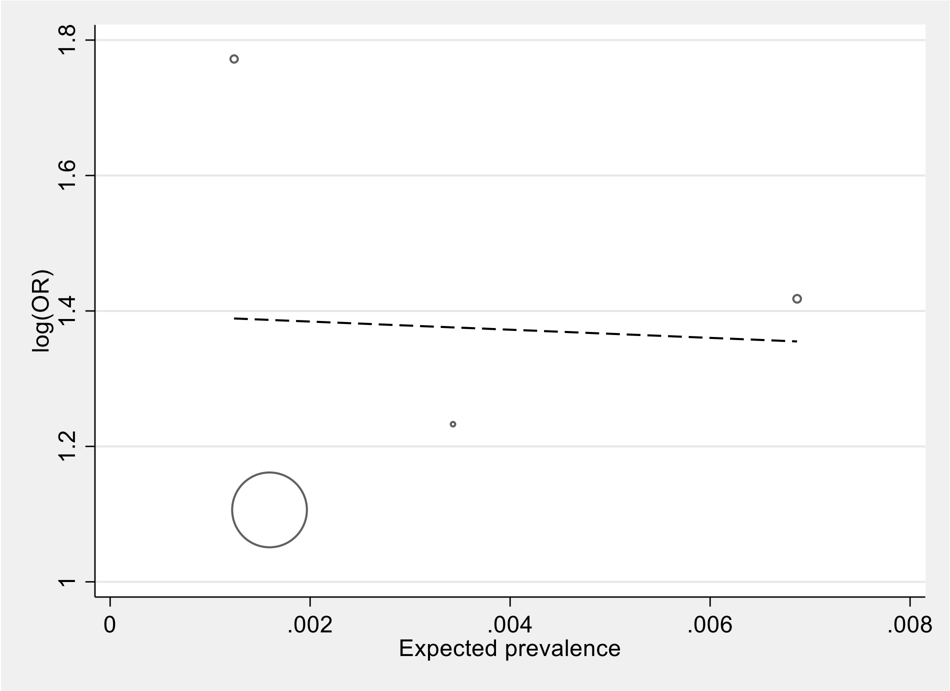

Figure S12: Relationship between expected prevalence (% with both conditions) and log odds ratio

3.16 Asthma and stroke

Table S16.1: Studies of association between asthma and stroke

Study	n	Asthma prevalence	Stroke prevalence	% with both	OR	SE of log(OR)	Expected % with both
DHS 2016	10301	3.6%	1.4%	0.24%	5.86	0.226	0.05%
Garin 2016	3836	7.7%	4.9%	0.84%	2.65	0.203	0.38%
Medscheme	1274501	5.5%	0.6%	0.07%	2.45	0.035	0.03%

OR = odds ratio (for the association between the two conditions), SE = standard error.

Table S16.2: Meta-regression results: predictors of log odds ratio

	Mean	Standard error
Expected prevalence term	-86.9	228.6
Constant	1.33	0.50

The variance of the random effects in the meta-regression model, on a log scale, is 0.367.

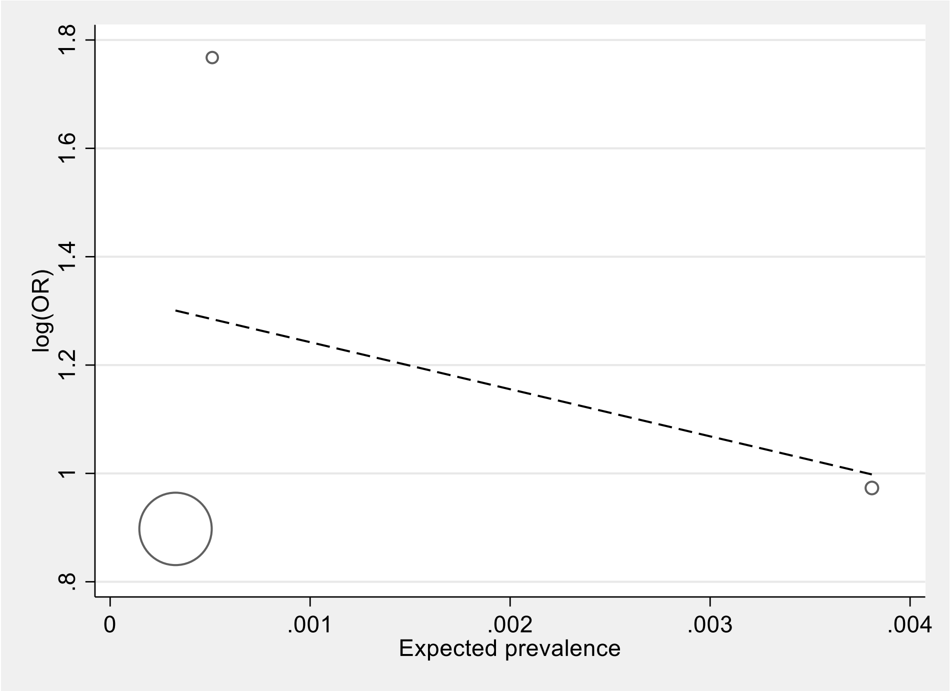

Figure S13: Relationship between expected prevalence (% with both conditions) and log odds ratio

3.17 Asthma and tuberculosis

Table S17.1: Studies of association between asthma and tuberculosis

Study	n	TB prevalence	Asthma prevalence	% with both	OR	SE of log(OR)	Expected % with both
DHS 2016	10308	1.3%	3.6%	0.11%	2.36	0.319	0.05%
Ehrlich 2005	13826	2.4%	3.8%	0.21%	2.57	0.200	0.09%

OR = odds ratio (for the association between the two conditions), SE = standard error.

Table S17.2: Meta-analysis results: average log odds ratio

	Mean	Standard error
Constant	0.92	0.17

The variance of the random effects in the meta-analysis model, on a log scale, is 0 (i.e. there is no significant evidence of heterogeneity between the two studies).

Johnson LF, *et al. BMJ Glob Health* 2024; 9:e013376. doi: 10.1136/bmjgh-2023-013376

3.18 COPD and depression

Table S18.1: Studies of association between COPD and depression

Study	n	Depression prevalence	COPD prevalence	% with both	OR	SE of log(OR)	Expected % with both
Folb 2015	4393	56.1%	26.3%	17.07%	1.63	0.071	14.78%
Pengpid 2020	5059	16.5%	1.5%	0.43%	2.16	0.257	0.24%
Garin 2016	3836	4.8%	4.3%	1.04%	7.90	0.201	0.20%
Medscheme	1274501	6.5%	0.8%	0.13%	3.10	0.027	0.05%

OR = odds ratio (for the association between the two conditions), SE = standard error.

Table S18.2: Meta-regression results: predictors of log odds ratio

	Mean	Standard error
Expected prevalence term	-5.72	5.07
Constant	1.34	0.38

The variance of the random effects in the meta-regression model, on a log scale, is 0.389.

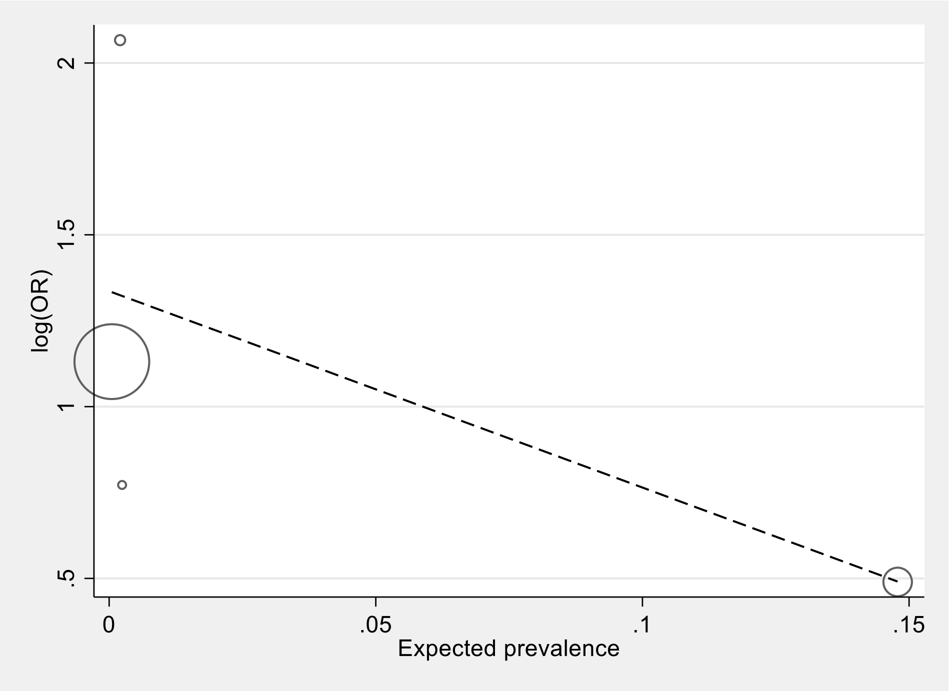

Figure S14: Relationship between expected prevalence (% with both conditions) and log odds ratio

3.19 COPD and diabetes

Table S19.1: Studies of association between COPD and diabetes

Study	n	Diabetes prevalence	COPD prevalence	% with both	OR	SE of log(OR)	Expected % with both
Folb 2015	4393	41.9%	26.3%	5.92%	0.30	0.079	11.04%
DHS 2016	6719	11.9%	1.3%	0.28%	2.07	0.262	0.16%
Garin 2016	3836	9.2%	4.3%	1.19%	4.27	0.184	0.39%
Lalkhen 2015	18856	5.0%	0.7%	0.07%	1.95	0.293	0.04%
Medscheme	1274501	10.7%	0.8%	0.22%	3.43	0.022	0.08%

OR = odds ratio (for the association between the two conditions), SE = standard error.

Table S19.2: Meta-regression results: predictors of log odds ratio

	Mean	Standard error
Expected prevalence term	-20.77	3.62
Constant	1.11	0.20

The variance of the random effects in the meta-regression model, on a log scale, is 0.104.

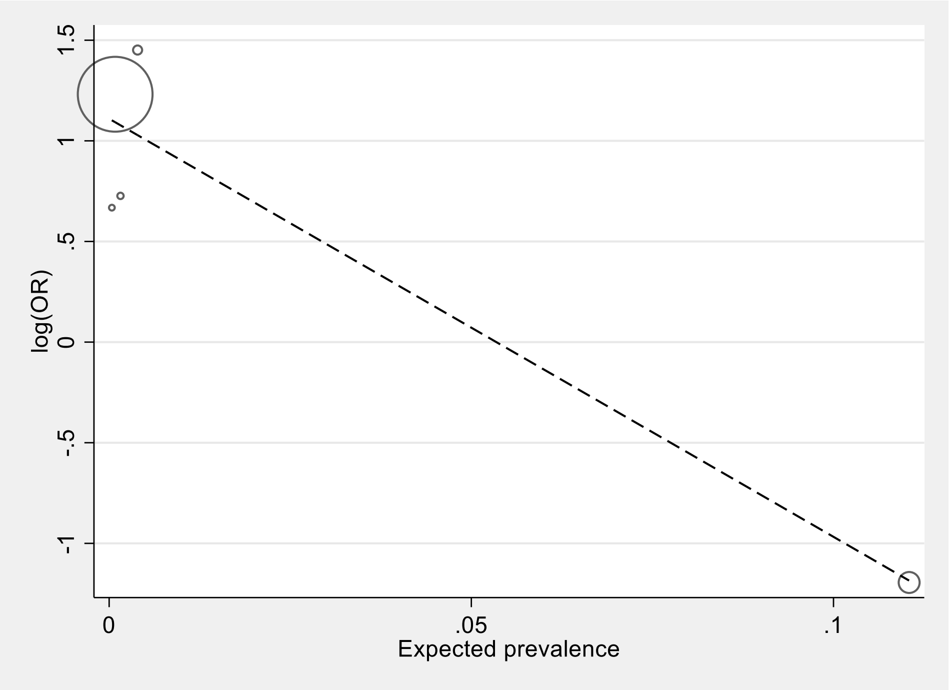

Figure S15: Relationship between expected prevalence (% with both conditions) and log odds ratio

3.20 COPD and HIV

Table S20.1: Studies of association between COPD and HIV

Study	n	HIV prevalence	COPD prevalence	% with both	OR	SE of log(OR)	Expected % with both
DHS 2016	6520	19.3%	1.3%	0.23%	0.88	0.286	0.25%
Medscheme	1274501	7.0%	0.8%	0.03%	0.58	0.051	0.05%

OR = odds ratio (for the association between the two conditions), SE = standard error.

Table S20.2: Meta-analysis results: average log odds ratio

	Mean	Standard error
Constant	-0.43	0.22

The variance of the random effects in the meta-analysis model, on a log scale, is 0.046.

Johnson LF, *et al. BMJ Glob Health* 2024; 9:e013376. doi: 10.1136/bmjgh-2023-013376

3.21 COPD and hypertension

Table S21.1: Studies of association between COPD and hypertension

Study	n	Hypertension prevalence	COPD prevalence	% with both	OR	SE of log(OR)	Expected % with both
Folb 2015	4393	73.5%	26.3%	16.8%	0.52	0.074	19.3%
DHS 2016	8223	44.7%	1.3%	0.9%	3.36	0.223	0.6%
Garin 2016	3836	78.3%	4.3%	3.3%	0.94	0.191	3.3%
Lalkhen 2015	18856	17.1%	0.7%	0.4%	4.53	0.170	0.1%
Medscheme	1274501	23.8%	0.8%	0.6%	8.38	0.023	0.2%

OR = odds ratio (for the association between the two conditions), SE = standard error.

Table S21.2: Meta-regression results: predictors of log odds ratio

	Mean	Standard error
Expected prevalence term	-11.31	4.61
Constant	1.37	0.41

The variance of the random effects in the meta-regression model, on a log scale, is 0.575.

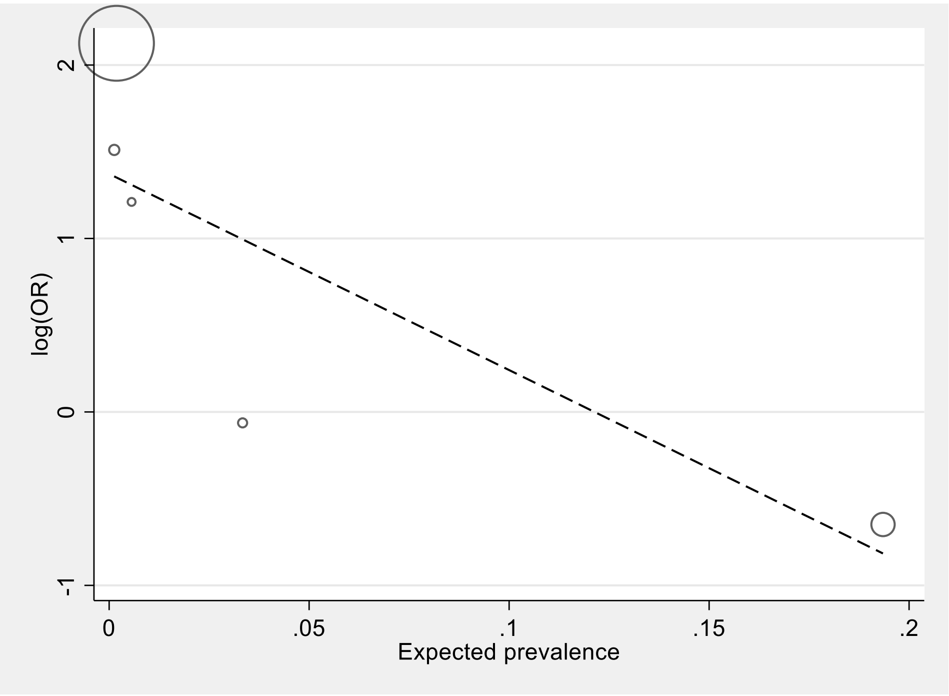

Figure S16: Relationship between expected prevalence (% with both conditions) and log odds ratio

3.22 COPD and ischaemic heart disease

Table S22.1: Studies of association between COPD and ischaemic heart disease (IHD)

Study	n	COPD prevalence	IHD prevalence	% with both	OR	SE of log(OR)	Expected % with both
DHS 2016	10278	1.3%	3.4%	0.26%	7.87	0.223	0.04%
Garin 2016	3836	4.3%	8.9%	1.68%	7.95	0.172	0.38%
Medscheme	1274501	0.8%	2.9%	0.17%	9.62	0.025	0.02%

OR = odds ratio (for the association between the two conditions), SE = standard error.

Table S22.2: Meta-regression results: predictors of log odds ratio

	Mean	Standard error
Expected prevalence term	-54.6	48.7
Constant	2.27	0.03

The variance of the random effects in the meta-regression model, on a log scale, is 0 (i.e. there is no significant heterogeneity across studies when including the expected prevalence term).

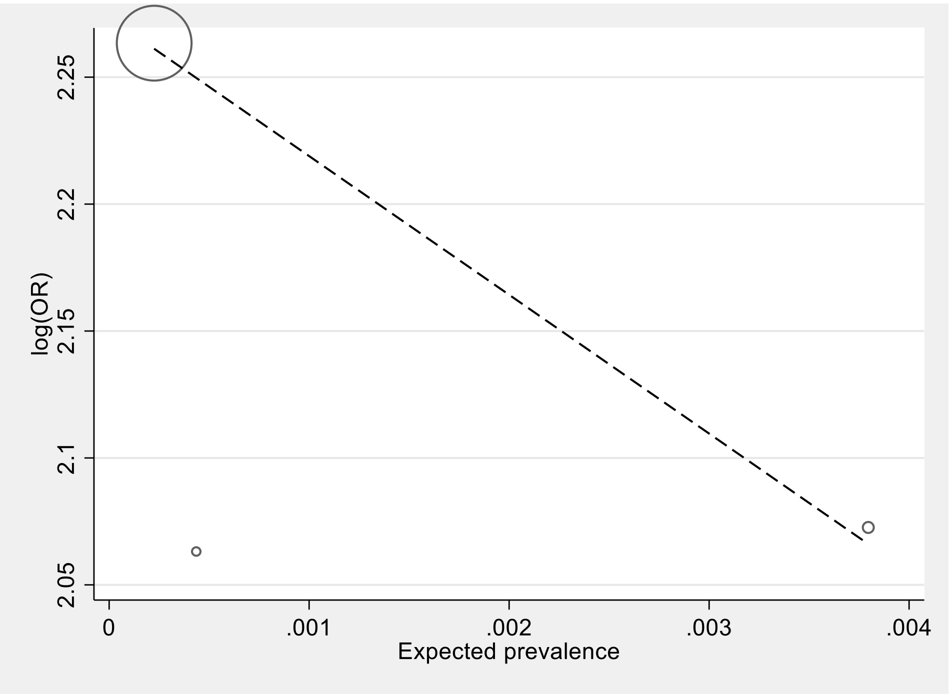

Figure S17: Relationship between expected prevalence (% with both conditions) and log odds ratio

3.23 COPD and stroke

Table S23.1: Studies of association between COPD and stroke

Study	n	COPD prevalence	Stroke prevalence	% with both	OR	SE of log(OR)	Expected % with both
DHS 2016	10284	1.3%	1.4%	0.088%	5.47	0.356	0.018%
Garin 2016	3836	4.3%	4.9%	0.527%	2.92	0.250	0.210%
Medscheme	1274501	0.8%	0.6%	0.030%	6.96	0.054	0.005%

OR = odds ratio (for the association between the two conditions), SE = standard error.

Table S23.2: Meta-regression results: predictors of log odds ratio

	Mean	Standard error
Expected prevalence term	-422.5	124.2
Constant	1.96	0.05

The variance of the random effects in the meta-regression model, on a log scale, is 0 (i.e. there is no significant heterogeneity across studies when including the expected prevalence term).

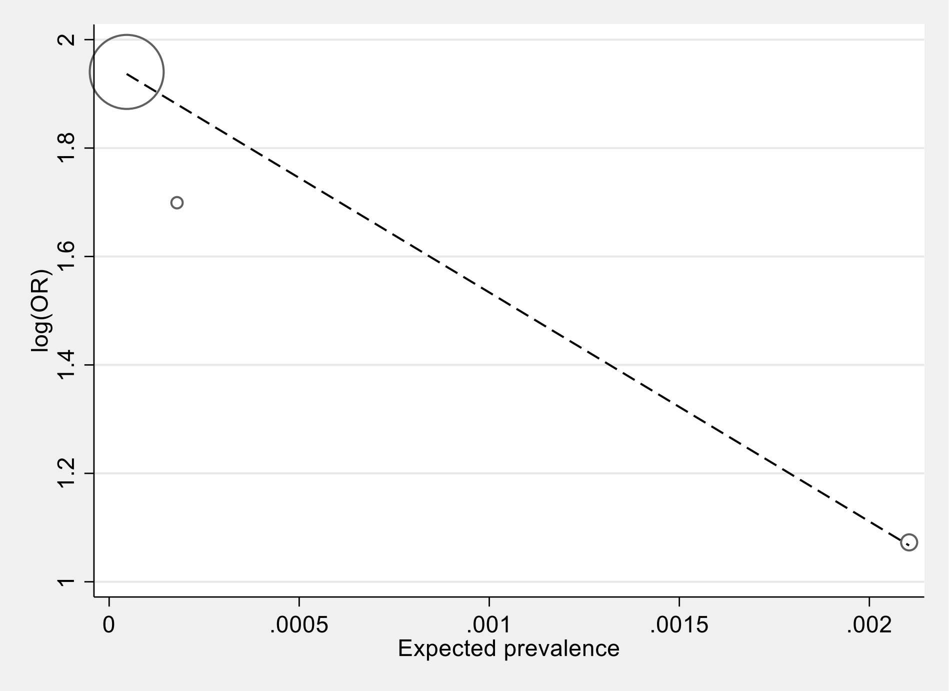

Figure S18: Relationship between expected prevalence (% with both conditions) and log odds ratio

Johnson LF, *et al. BMJ Glob Health* 2024; 9:e013376. doi: 10.1136/bmjgh-2023-013376

3.24 COPD and tuberculosis

Table S24.1: Studies of association between COPD and tuberculosis

Study	n	TB prevalence	COPD prevalence	% with both	OR	SE of log(OR)	Expected % with both
DHS 2016	10290	1.3%	1.3%	0.03%	1.73	0.590	0.02%
Jithoo 2006	847	15.1%	23.1%	7.44%	4.27	0.201	3.50%
Ehrlich 2004	13827	2.4%	2.6%	0.39%	8.59	0.160	0.06%

OR = odds ratio (for the association between the two conditions), SE = standard error.

Table S24.2: Meta-regression results: predictors of log odds ratio

	Mean	Standard error
Expected prevalence term	0.49	38.54
Constant	1.44	0.81

The variance of the random effects in the meta-regression model, on a log scale, is 1.100.

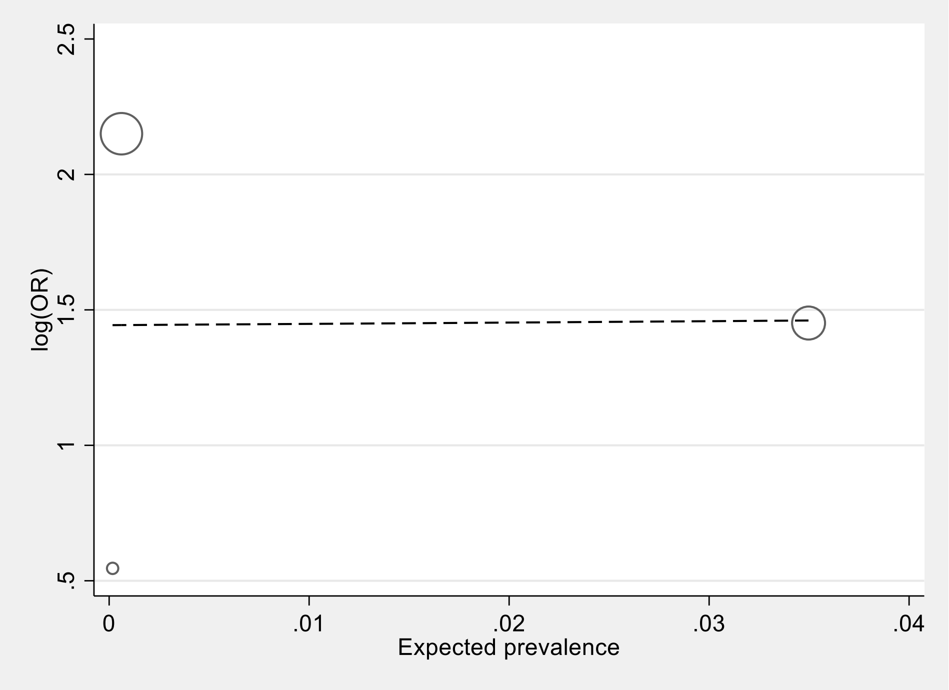

Figure S19: Relationship between expected prevalence (% with both conditions) and log odds ratio

29

3.25 Depression and diabetes

Table S25.1: Studies of association between diabetes and depression

Study	n	Diabetes prevalence	Depression prevalence	% with both	OR	SE of log(OR)	Expected % with both
Folb 2015	4393	41.9%	56.1%	18.98%	0.47	0.062	23.54%
Chang 2019	3889	11.5%	16.7%	2.86%	1.78	0.119	1.92%
Petersen 2019	2549	9.3%	9.1%	0.47%	0.51	0.304	0.85%
Garin 2016	3836	9.2%	4.8%	0.73%	1.83	0.214	0.44%
Medscheme	1274501	10.7%	6.5%	1.19%	1.99	0.009	0.69%
WHS	2233	8.4%	9.2%	1.57%	2.54	0.204	0.77%
Pengpid 2018	22633	2.7%	13.0%	0.51%	1.60	0.106	0.35%

OR = odds ratio (for the association between the two conditions), SE = standard error.

Table S25.2: Meta-regression results: predictors of log odds ratio

	Mean	Standard error
Expected prevalence term	-5.45	2.31
Constant	0.52	0.22

The variance of the random effects in the meta-regression model, on a log scale, is 0.188.

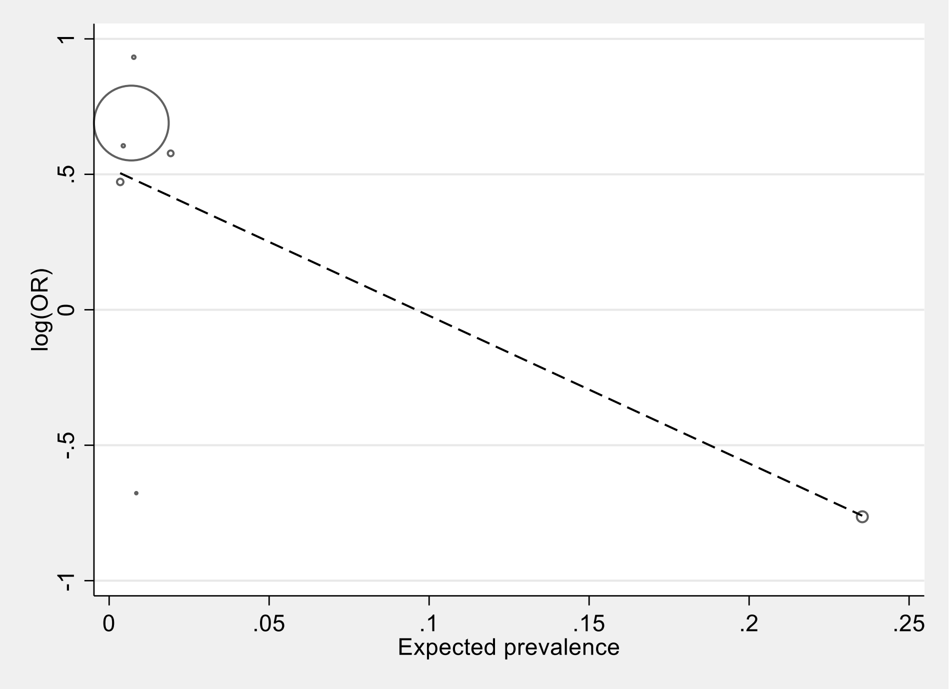

Figure S20: Relationship between expected prevalence (% with both conditions) and log odds ratio

Johnson LF, *et al. BMJ Glob Health* 2024; 9:e013376. doi: 10.1136/bmjgh-2023-013376

3.26 Depression and HIV

Table S26.1: Studies of association between depression and HIV

Study	n	HIV prevalence	Depression prevalence	% with both	OR	SE of log(OR)	Expected % with both
Chang 2019	3889	23.2%	16.7%	3.0%	0.69	0.110	3.9%
Petersen 2019	2549	61.3%	9.1%	6.9%	2.09	0.159	5.6%
Van Heerden 2017	570	33.2%	11.9%	2.5%	0.48	0.314	4.0%
Negin 2012	3048	6.3%	4.6%	0.5%	1.70	0.292	0.3%
Medscheme	1274501	7.0%	6.5%	0.5%	1.05	0.014	0.5%

OR = odds ratio (for the association between the two conditions), SE = standard error.

Table S26.2: Meta-regression results: predictors of log odds ratio

	Mean	Standard error
Expected prevalence term	-1.69	14.55
Constant	0.10	0.52

The variance of the random effects in the meta-regression model, on a log scale, is 0.423.

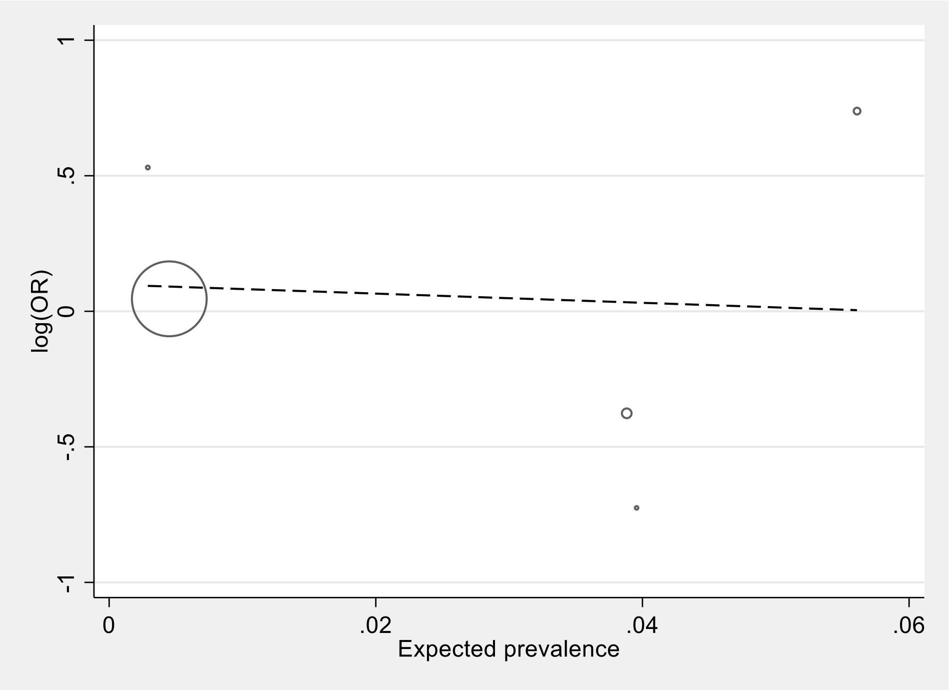

Figure S21: Relationship between expected prevalence (% with both conditions) and log odds ratio

Johnson LF, *et al. BMJ Glob Health* 2024; 9:e013376. doi: 10.1136/bmjgh-2023-013376

3.27 Depression and hypertension

Table S27.1: Studies of association between depression and hypertension

Study	n	Hypertension prevalence	Depression prevalence	% with both	OR	SE of log(OR)	Expected % with both
Folb 2015	4393	73.5%	56.1%	36.5%	0.35	0.075	41.2%
Chang 2019	3889	64.3%	16.7%	11.6%	1.30	0.092	10.8%
Petersen 2019	2549	52.6%	9.1%	3.7%	0.59	0.140	4.8%
Grimsrud 2009	4351	17.6%	4.9%	1.1%	1.42	0.168	0.9%
Garin 2016	3836	78.3%	4.8%	3.7%	0.97	0.182	3.8%
Medscheme	1274501	23.8%	6.5%	2.8%	2.66	0.007	1.5%
Pengpid 2018	22633	9.6%	13.0%	1.7%	1.54	0.060	1.2%

OR = odds ratio (for the association between the two conditions), SE = standard error.

Table S27.2: Meta-regression results: predictors of log odds ratio

	Mean	Standard error
Expected prevalence term	-3.57	1.32
Constant	0.40	0.22

The variance of the random effects in the meta-regression model, on a log scale, is 0.214.

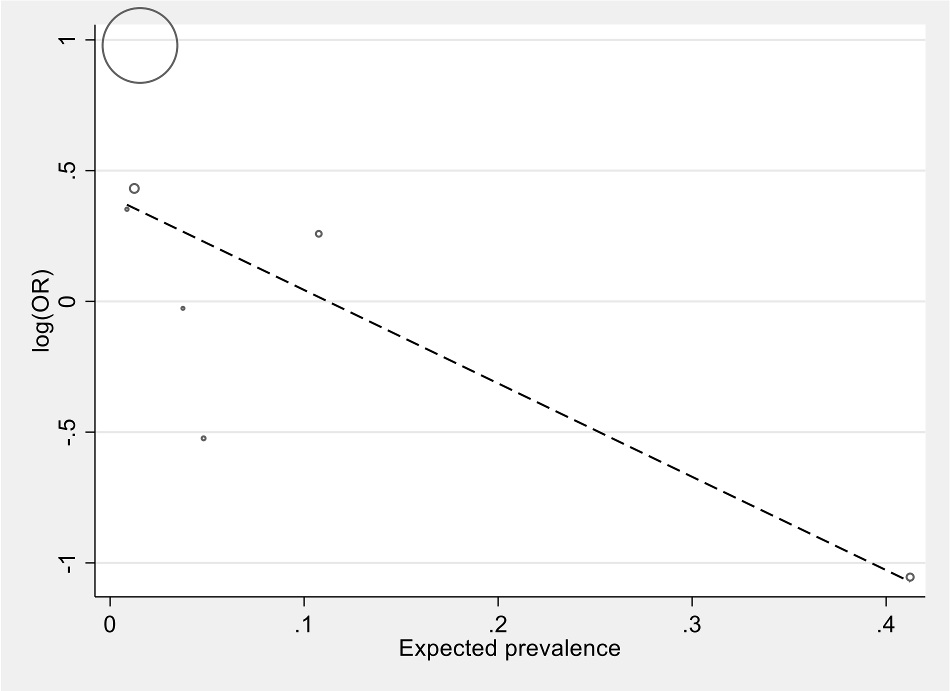

Figure S22: Relationship between expected prevalence (% with both conditions) and log odds ratio

Johnson LF, *et al. BMJ Glob Health* 2024; 9:e013376. doi: 10.1136/bmjgh-2023-013376

3.28 Depression and ischaemic heart disease

Table S28.1: Studies of association between depression and ischaemic heart disease (IHD)

Study	n	Depression prevalence	IHD prevalence	% with both	OR	SE of log(OR)	Expected % with both
Chang 2019	3889	16.7%	9.9%	2.67%	2.01	0.124	1.7%
Garin 2016	3836	4.8%	8.9%	1.34%	4.49	0.175	0.4%
Medscheme	1274501	6.5%	2.9%	0.42%	2.57	0.015	0.2%
WHS	2236	9.3%	5.1%	0.98%	2.50	0.249	0.5%

OR = odds ratio (for the association between the two conditions), SE = standard error.

Table S28.2: Meta-regression results: predictors of log odds ratio

	Mean	Standard error
Expected prevalence term	-27.76	29.90
Constant	1.20	0.27

The variance of the random effects in the meta-regression model, on a log scale, is 0.102.

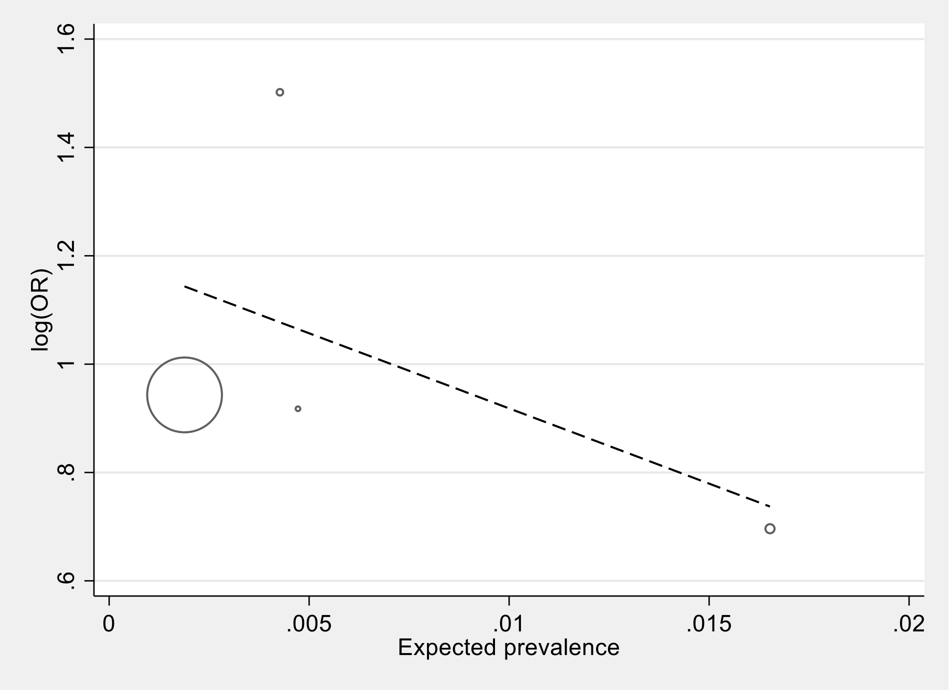

Figure S23: Relationship between expected prevalence (% with both conditions) and log odds ratio

3.29 Depression and stroke

Table S29.1: Studies of association between depression and stroke

Study	n	Depression prevalence	Stroke prevalence	% with both	OR	SE of log(OR)	Expected % with both
Pengpid 2020	5059	16.5%	2.5%	0.79%	2.33	0.194	0.42%
Garin 2016	3836	4.8%	4.9%	0.85%	4.81	0.210	0.24%
Medscheme	1274501	6.5%	0.6%	0.12%	3.55	0.029	0.04%

OR = odds ratio (for the association between the two conditions), SE = standard error.

Table S29.2: Meta-regression results: predictors of log odds ratio

	Mean	Standard error
Expected prevalence term	-98.2	149.1
Constant	1.44	0.4

The variance of the random effects in the meta-regression model, on a log scale, is 0.145.

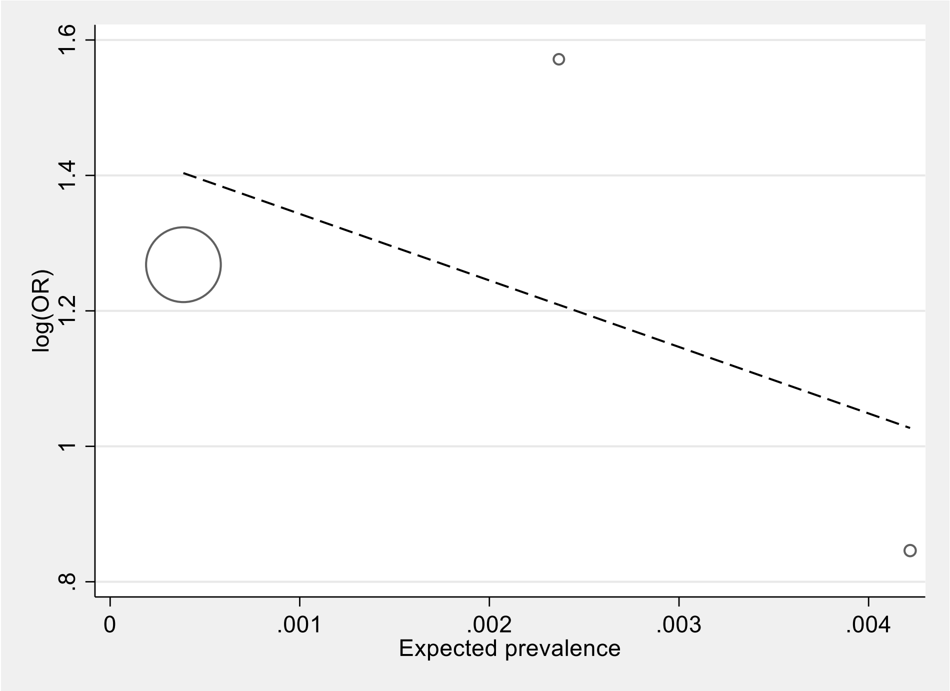

Figure S24: Relationship between expected prevalence (% with both conditions) and log odds ratio

3.30 Depression and tuberculosis

Table S30.1: Studies of association between depression and tuberculosis

Study	n	TB prevalence	Depression prevalence	% with both	OR	SE of log(OR)	Expected % with both
Koyanagi 2017	199764	1.7%	6.9%	0.40%	4.39	0.041	0.1%
de Araujo 2014	1434	50.0%	35.8%	19.39%	1.30	0.110	17.9%
Hernandez Sarmiento 2013	426	7.9%	15.7%	-	2.66	0.376	1.2%

Note that due to an absence of South African data, we have relied only on data from international studies [23]. OR = odds ratio (for the association between the two conditions), SE = standard error.

Table S30.2: Meta-regression results: predictors of log odds ratio

	Mean	Standard error
Expected prevalence term	-6.61	1.28
Constant	1.44	0.14

The variance of the random effects in the meta-regression model, on a log scale, is 0.020.

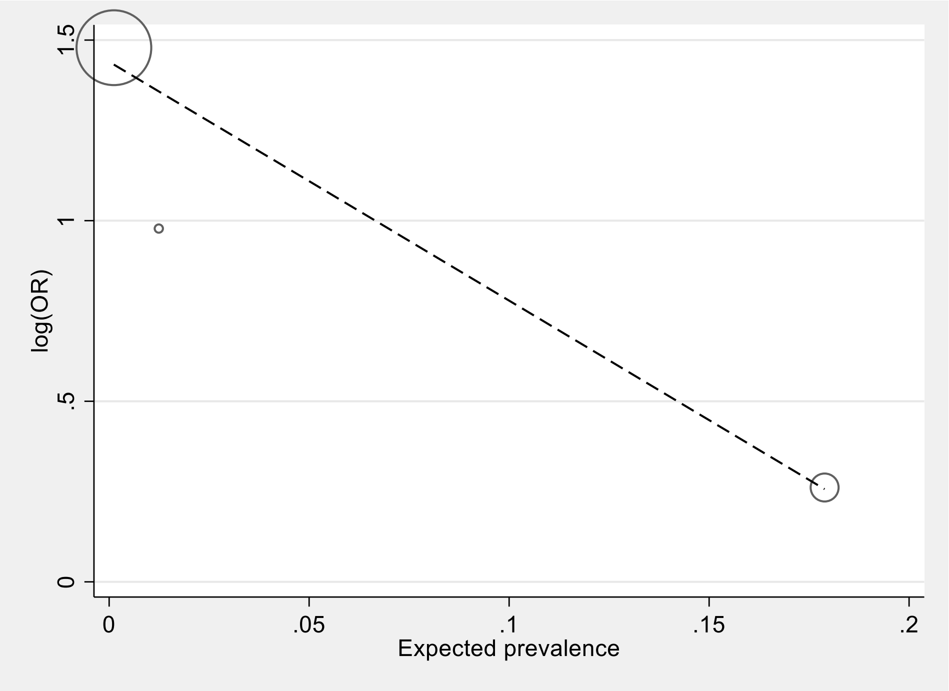

Figure S25: Relationship between expected prevalence (% with both conditions) and log odds ratio

Johnson LF, *et al. BMJ Glob Health* 2024; 9:e013376. doi: 10.1136/bmjgh-2023-013376

3.31 Diabetes and HIV

Table S31.1: Studies of association between diabetes and HIV

Study	n	Diabetes prevalence	HIV prevalence	% with both	OR	SE of log(OR)	Expected % with both
Wong 2021	17052	9.7%	34.0%	2.13%	0.52	0.062	3.3%
Chang 2019	3889	11.5%	23.2%	1.79%	0.58	0.136	2.7%
Weimann 2016: wave 1	18526	2.8%	1.1%	0.01%	0.29	0.772	0.0%
Weimann 2016: wave 3	20015	2.7%	2.1%	0.14%	2.64	0.199	0.1%
Oni 2015	14364	18.3%	38.4%	1.24%	0.09	0.080	7.0%
DHS 2016	6533	11.5%	19.3%	1.52%	0.60	0.113	2.2%
Van Heerden 2017	570	4.0%	33.2%	0.70%	0.41	0.557	1.3%
Sharman 2019	15021	6.4%	25.3%	1.21%	0.67	0.085	1.6%
Negin 2012	3058	9.4%	6.3%	0.26%	0.40	0.367	0.6%
PHDC	2568172	9.2%	13.4%	0.59%	0.41	0.009	1.2%

OR = odds ratio (for the association between the two conditions), SE = standard error.

Table S31.2: Meta-regression results: predictors of log odds ratio

	Mean	Standard error
Expected prevalence term	-32.72	8.52
Constant	0.05	0.25

The variance of the random effects in the meta-regression model, on a log scale, is 0.223.

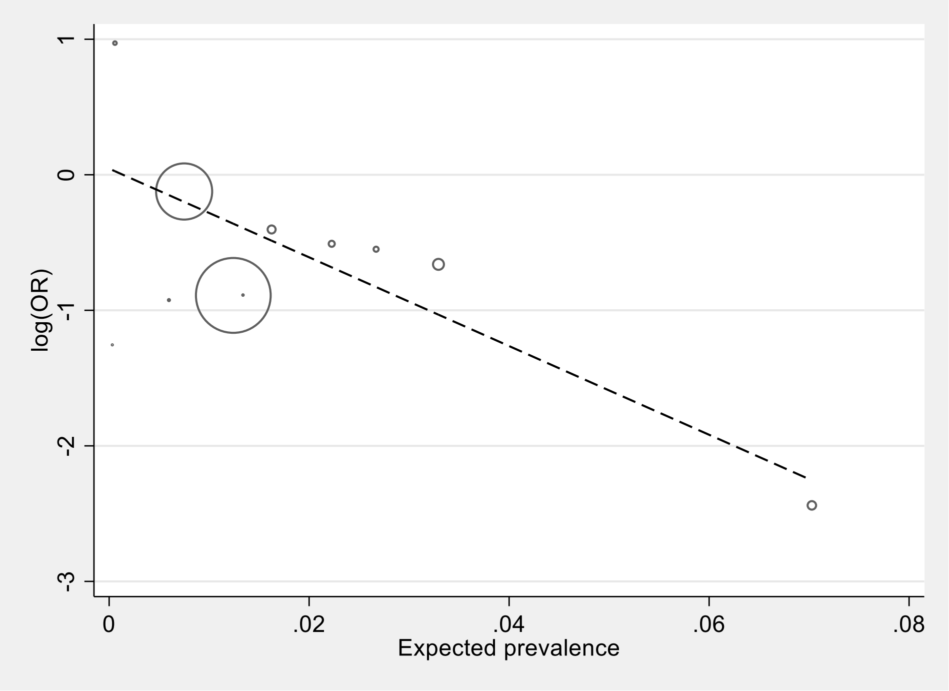

Figure S26: Relationship between expected prevalence (% with both conditions) and log odds ratio

Johnson LF, *et al. BMJ Glob Health* 2024; 9:e013376. doi: 10.1136/bmjgh-2023-013376

3.32 Diabetes and hypertension

Table S32.1: Studies of association between diabetes and hypertension

Study	n	Hypertension prevalence	Diabetes prevalence	% with both	OR	SE of log(OR)	Expected % with both
Wong 2021	17052	25.8%	9.7%	6.39%	7.10	0.056	2.5%
Folb 2015	4393	73.5%	41.9%	35.06%	2.61	0.076	30.8%
Sewpaul 2021	3832	38.4%	12.7%	9.47%	5.87	0.110	4.9%
Chang 2019	3889	64.3%	11.5%	9.10%	2.29	0.122	7.4%
Weimann 2016: wave 1	18526	22.7%	2.8%	1.94%	8.16	0.096	0.6%
Weimann 2016: wave 3	20015	32.1%	2.7%	2.06%	7.10	0.102	0.9%
Oni 2015	14364	64.6%	18.3%	15.66%	3.97	0.059	11.8%
DHS 2016	7050	44.7%	11.7%	8.16%	3.25	0.080	5.2%
Garin 2016	3836	78.3%	9.2%	8.45%	3.36	0.198	7.2%
Sharman 2019	15023	23.8%	6.4%	4.84%	12.00	0.078	1.5%
Lalkhen 2015	18856	17.1%	5.0%	3.11%	9.49	0.070	0.9%
PHDC	2568172	20.9%	9.2%	6.17%	10.31	0.005	1.9%
Medscheme	1274501	23.8%	10.7%	7.93%	13.12	0.007	2.6%

OR = odds ratio (for the association between the two conditions), SE = standard error.

Table S32.2: Meta-regression results: predictors of log odds ratio

	Mean	Standard error
Expected prevalence term	-4.77	1.65
Constant	2.07	0.16

The variance of the random effects in the meta-regression model, on a log scale, is 0.209.

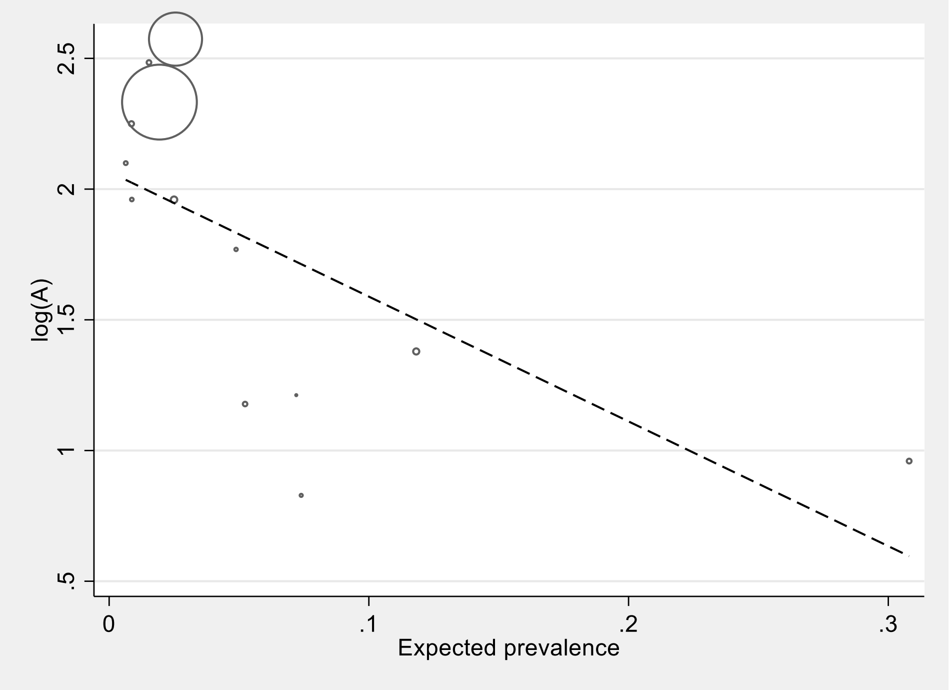

Figure S27: Relationship between expected prevalence (% with both conditions) and log odds ratio

3.33 Diabetes and ischaemic heart disease

Table S33.1: Studies of association between diabetes and ischaemic heart disease (IHD)

Study	n	Diabetes prevalence	IHD prevalence	% with both	OR	SE of log(OR)	Expected % with both
Sewpaul 2021	3832	12.7%	5.2%	1.28%	2.34	0.172	0.7%
Chang 2019	3889	11.5%	9.9%	1.27%	1.16	0.161	1.1%
DHS 2016	6731	11.9%	4.1%	1.00%	2.51	0.146	0.5%
Garin 2016	3836	9.2%	8.9%	2.04%	3.47	0.143	0.8%
Medscheme	1274501	10.7%	2.9%	1.21%	6.47	0.011	0.3%
WHS	2238	8.5%	5.0%	1.43%	4.92	0.225	0.4%

OR = odds ratio (for the association between the two conditions), SE = standard error.

Table S33.2: Meta-regression results: predictors of log odds ratio

	Mean	Standard error
Expected prevalence term	-173.1	53.8
Constant	2.22	0.37

The variance of the random effects in the meta-regression model, on a log scale, is 0.112.

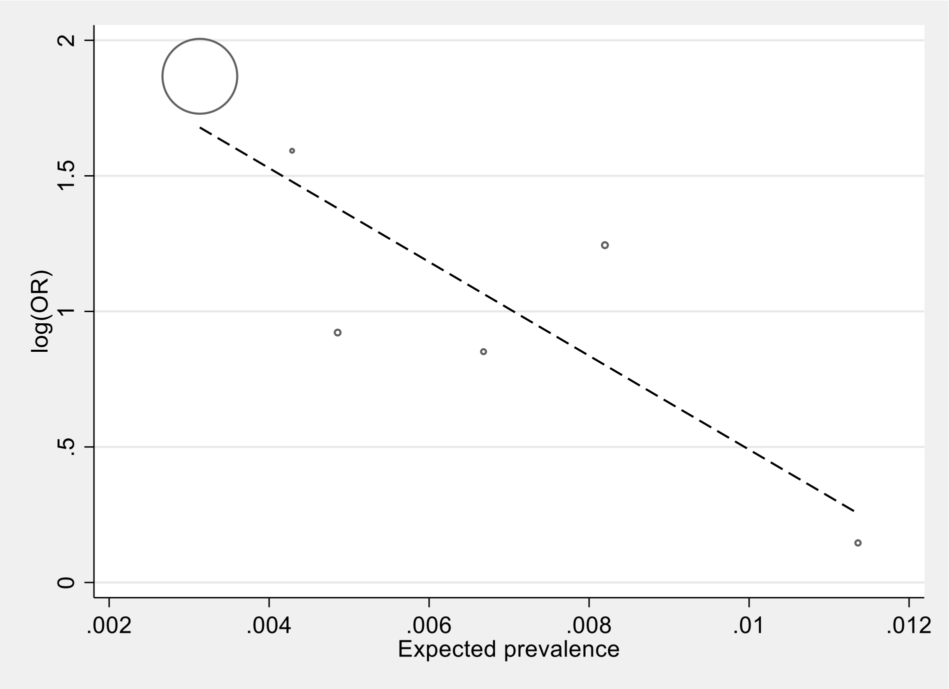

Figure S28: Relationship between expected prevalence (% with both conditions) and log odds ratio

3.34 Diabetes and stroke

Table S34.1: Studies of association between diabetes and stroke

Study	n	Diabetes prevalence	Stroke prevalence	% with both	OR	SE of log(OR)	Expected % with both
Sewpaul 2021	3832	12.7%	2.9%	0.73%	2.39	0.224	0.37%
DHS 2016	6733	11.9%	1.4%	0.28%	1.82	0.259	0.17%
Garin 2016	3836	9.2%	4.9%	0.83%	2.10	0.203	0.45%
Medscheme	1274501	10.7%	0.6%	0.21%	4.51	0.024	0.06%

OR = odds ratio (for the association between the two conditions), SE = standard error.

Table S34.2: Meta-regression results: predictors of log odds ratio

	Mean	Standard error
Expected prevalence term	-156.3	118.8
Constant	1.38	0.35

The variance of the random effects in the meta-regression model, on a log scale, is 0.111.

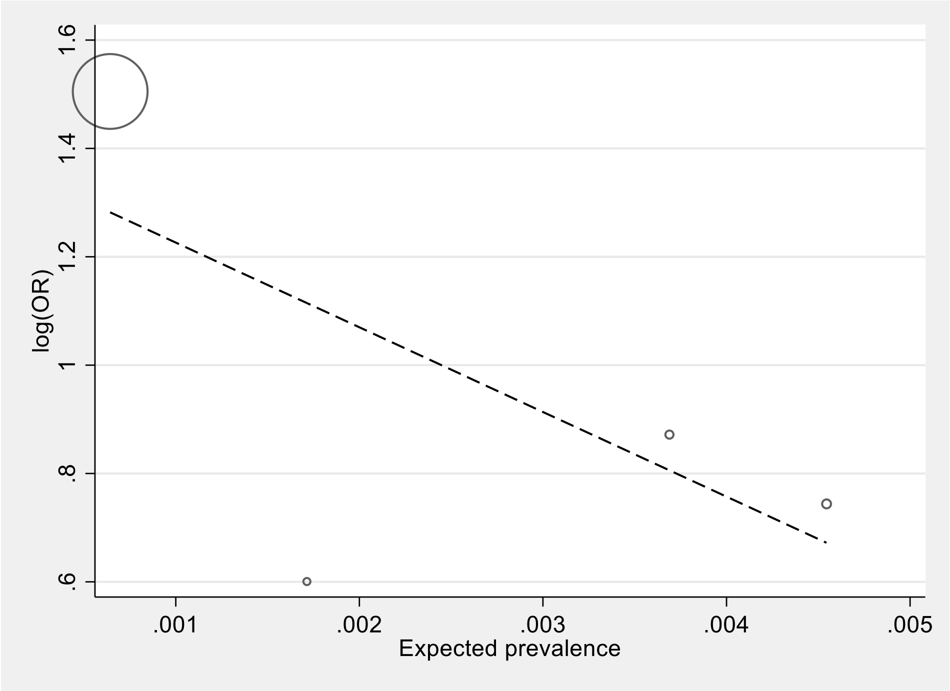

Figure S29: Relationship between expected prevalence (% with both conditions) and log odds ratio

3.35 Diabetes and tuberculosis

Table S35.1: Studies of association between diabetes and tuberculosis

Study	n	Hypertension prevalence	Diabetes prevalence	% with both	OR	SE of log(OR)	Expected % with both
Wong 2021	17052	9.7%	1.3%	0.18%	1.45	0.198	0.13%
Weimann 2016: wave 1	18526	2.8%	1.6%	0.05%	1.19	0.328	0.04%
Weimann 2016: wave 3	20015	2.7%	0.6%	0.05%	3.40	0.332	0.02%
Oni 2015	14364	18.3%	2.7%	0.27%	0.48	0.171	0.49%
DHS 2016	6750	11.9%	1.6%	0.24%	1.34	0.274	0.18%
Sharman 2019	15021	6.4%	2.8%	0.48%	3.24	0.134	0.18%
PHDC	2568172	9.2%	1.1%	0.09%	0.89	0.022	0.10%

OR = odds ratio (for the association between the two conditions), SE = standard error.

Table S35.2: Meta-regression results: predictors of log odds ratio

	Mean	Standard error
Expected prevalence term	-274.3	155.6
Constant	0.79	0.35

The variance of the random effects in the meta-regression model, on a log scale, is 0.321.

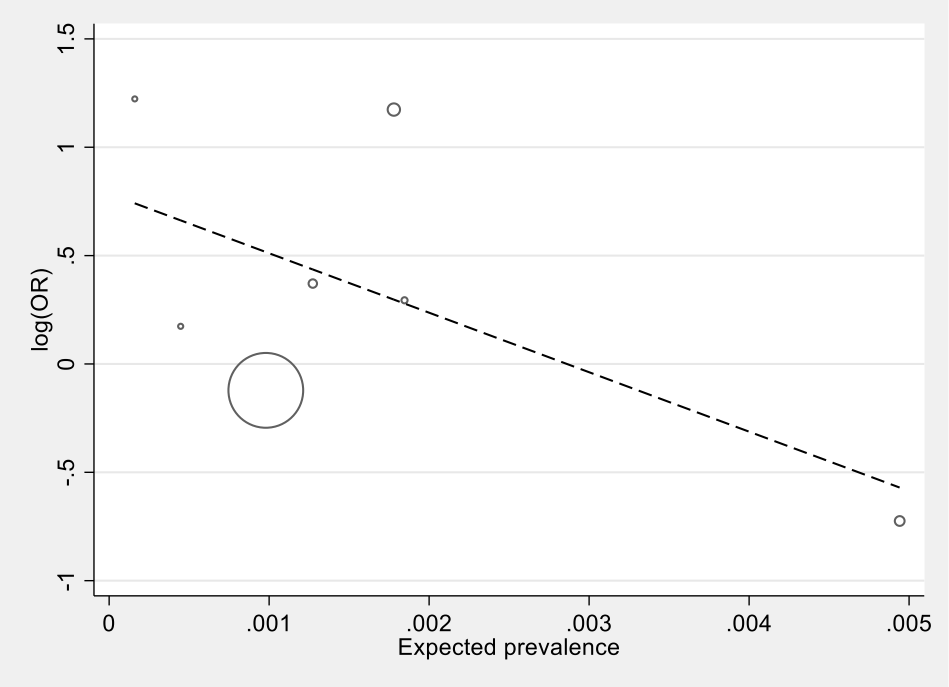

Figure S30: Relationship between expected prevalence (% with both conditions) and log odds ratio

3.36 HIV and hypertension

Table S36.1: Studies of association between HIV and hypertension

Study	n	Hypertension prevalence	HIV prevalence	% with both	OR	SE of log(OR)	Expected % with both
Wong 2021	17052	25.8%	34.0%	6.77%	0.62	0.039	8.8%
Chang 2019	3889	64.3%	23.2%	11.04%	0.40	0.078	14.9%
Weimann 2016: wave 1	18526	22.7%	1.1%	0.31%	1.32	0.157	0.3%
Weimann 2016: wave 3	20015	32.1%	2.1%	0.76%	1.18	0.102	0.7%
Oni 2015	14364	64.6%	38.4%	5.73%	0.01	0.064	24.8%
DHS 2016	6517	44.8%	19.4%	8.41%	0.93	0.063	8.7%
Van Heerden 2017	570	33.3%	33.2%	8.07%	0.53	0.200	11.1%
Sharman 2019	15023	23.8%	25.3%	4.70%	0.66	0.047	6.0%
Negin 2012	3161	68.0%	6.3%	3.99%	0.79	0.152	4.3%
PHDC	2568172	20.9%	13.4%	2.04%	0.64	0.005	2.8%
Medscheme	1274501	23.8%	7.0%	1.56%	0.92	0.008	1.7%

OR = odds ratio (for the association between the two conditions), SE = standard error.

Table S36.2: Meta-regression results: predictors of log odds ratio

	Mean	Standard error
Expected prevalence term	-17.00	3.01
Constant	0.60	0.31

The variance of the random effects in the meta-regression model, on a log scale, is 0.480.

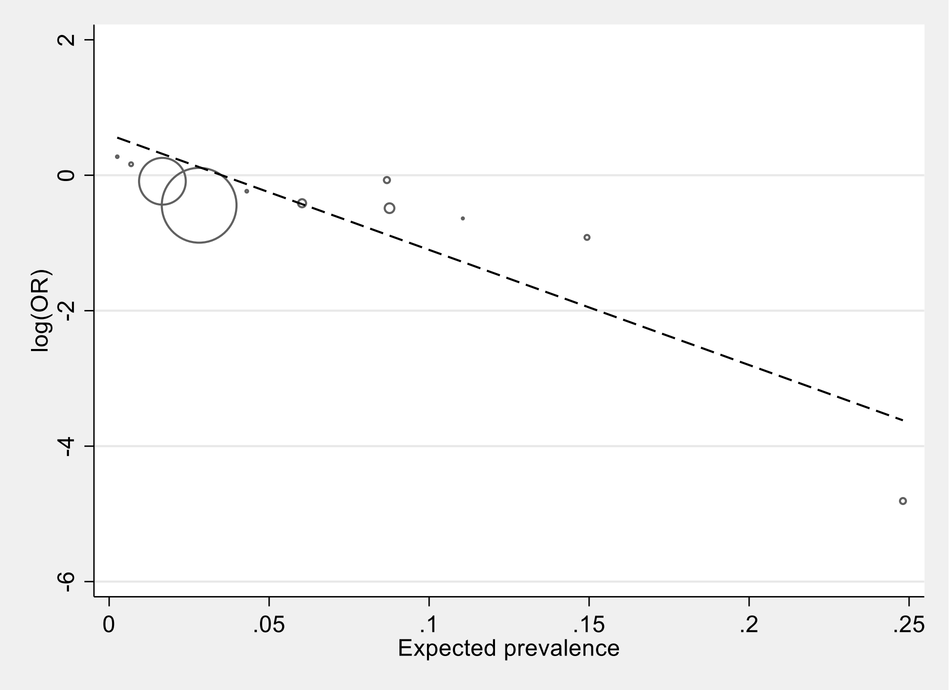

Figure S31: Relationship between expected prevalence (% with both conditions) and log odds ratio

3.37 HIV and ischaemic heart disease

Table S37.1: Studies of association between HIV and ischaemic heart disease (IHD)

Study	n	HIV prevalence	IHD prevalence	% with both	OR	SE of log(OR)	Expected % with both
Chang 2019	3889	23.2%	9.9%	1.82%	0.72	0.138	2.3%
DHS 2016	6532	19.3%	4.1%	0.75%	0.94	0.161	0.8%
Negin 2012	2893	6.5%	6.5%	0.55%	1.37	0.273	0.4%
Medscheme	1274501	7.0%	2.9%	0.06%	0.28	0.036	0.2%

OR = odds ratio (for the association between the two conditions), SE = standard error.

Table S37.2: Meta-regression results: predictors of log odds ratio

	Mean	Standard error
Expected prevalence term	13.69	49.40
Constant	-0.48	0.61

The variance of the random effects in the meta-regression model, on a log scale, is 0.631.

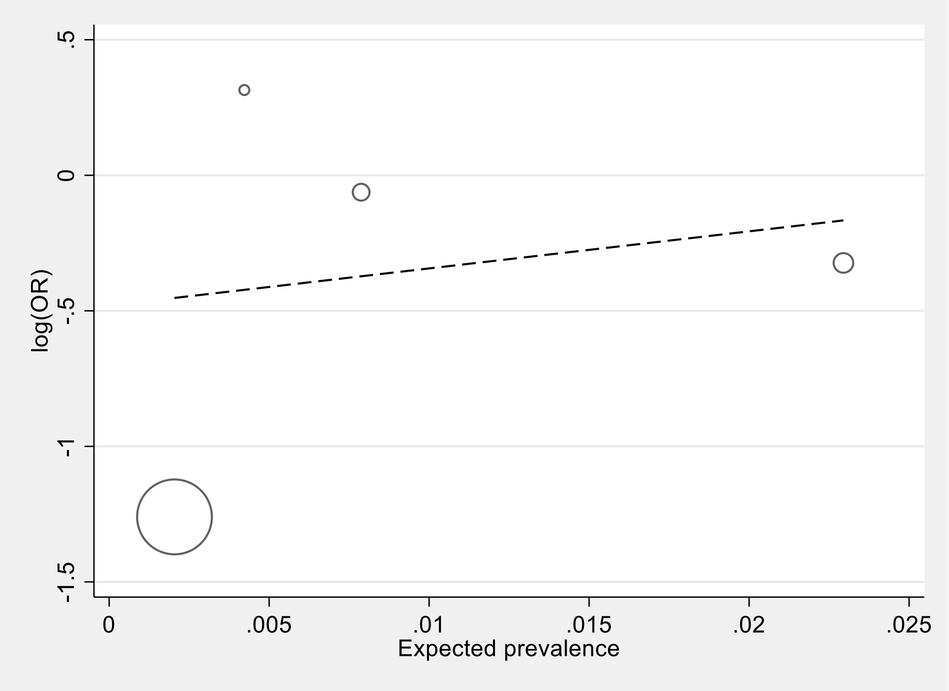

Figure S32: Relationship between expected prevalence (% with both conditions) and log odds ratio

3.38 HIV and stroke

Table S38.1: Studies of association between HIV and stroke

Study	n	HIV prevalence	Stroke prevalence	% with both	OR	SE of log(OR)	Expected % with both
DHS 2016	6534	19.3%	1.4%	0.31%	1.14	0.254	0.28%
Negin 2012	3055	6.4%	3.9%	0.16%	0.64	0.463	0.25%
Medscheme	1274501	7.0%	0.6%	0.03%	0.80	0.050	0.04%

OR = odds ratio (for the association between the two conditions), SE = standard error.

Table S38.2: Meta-regression results: predictors of log odds ratio

	Mean	Standard error
Expected prevalence term	97.8	124.3
Constant	-0.27	0.17

The variance of the random effects in the meta-regression model, on a log scale, is 0.004.

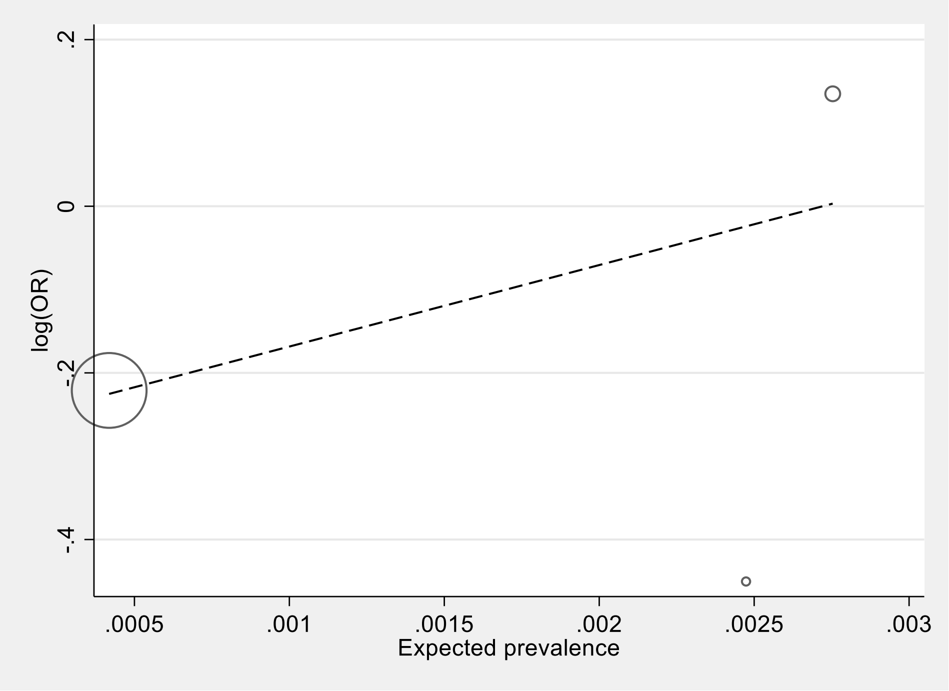

Figure S33: Relationship between expected prevalence (% with both conditions) and log odds ratio

3.39 HIV and tuberculosis

Table S39.1: Studies of association between HIV and TB

Study	n	HIV prevalence	TB prevalence	% with both	OR	SE of log(OR)	Expected % with both
Wong 2021	17052	34.0%	1.3%	0.67%	2.07	0.135	0.45%
Weimann 2016: wave 1	18526	1.1%	1.6%	0.12%	8.38	0.229	0.02%
Weimann 2016: wave 3	20015	2.1%	0.6%	0.03%	2.10	0.454	0.01%
Oni 2015	14364	38.4%	2.7%	1.78%	3.22	0.109	1.04%
DHS 2016	6551	19.4%	1.6%	0.92%	6.05	0.202	0.30%
Sharman 2019	14978	25.4%	2.8%	2.00%	8.13	0.111	0.70%
PHDC	2568172	13.4%	1.1%	0.45%	4.85	0.012	0.14%
Middelkoop 2010a	758	23.0%	3.0%	2.11%	8.35	0.462	0.70%
Middelkoop 2010b	1207	25.4%	1.7%	0.91%	3.70	0.454	0.42%
Corbett 2004	1734	26.1%	2.7%	0.98%	1.63	0.309	0.71%

OR = odds ratio (for the association between the two conditions), SE = standard error.

Table S39.2: Meta-regression results: predictors of log odds ratio

	Mean	Standard error
Expected prevalence term	-26.41	62.65
Constant	1.55	0.35

The variance of the random effects in the meta-regression model, on a log scale, is 0.343.

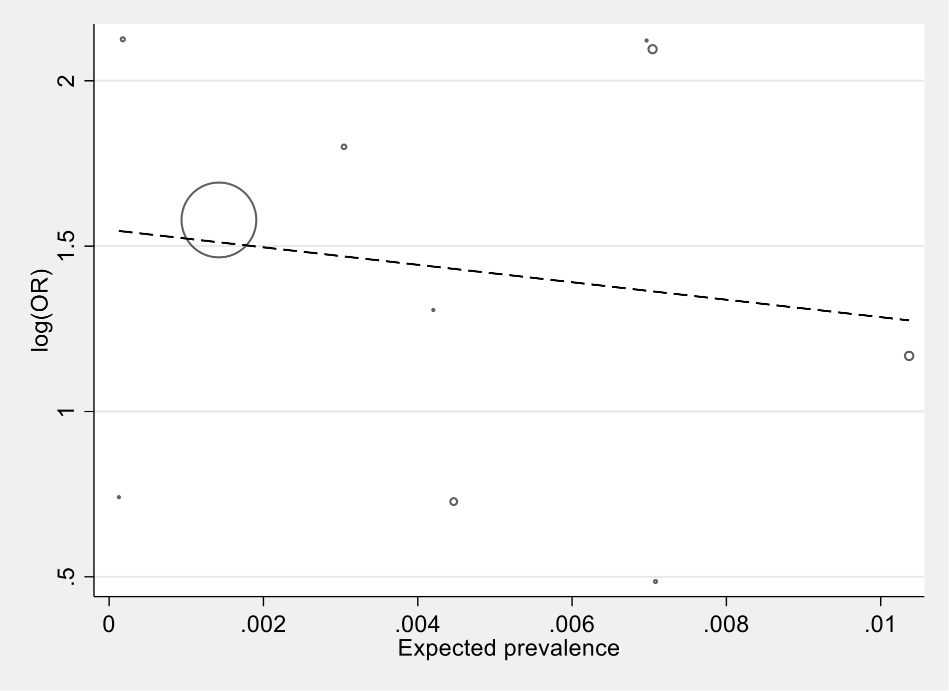

Figure S34: Relationship between expected prevalence (% with both conditions) and log odds ratio

3.40 Hypertension and ischaemic heart disease

Table S40.1: Studies of association between hypertension and ischaemic heart disease

Study	n	Hypertension prevalence	IHD prevalence	n with both	OR	SE of log(OR)	Expected % with both
Sewpaul 2021	3832	38.4%	5.2%	3.08%	2.40	0.147	2.0%
Chang 2019	3889	64.3%	9.9%	7.14%	1.50	0.119	6.4%
DHS 2016	8234	44.7%	3.8%	2.70%	3.21	0.127	1.7%
Garin 2016	3836	78.3%	8.9%	7.18%	1.16	0.143	7.0%
Lalkhen 2015	18856	17.1%	0.7%	0.36%	4.53	0.170	0.1%
Medscheme	1274501	23.8%	2.9%	2.37%	15.29	0.013	0.7%

IHD = ischaemic heart disease, OR = odds ratio (for the association between the two conditions), SE = standard error.

Table S40.2: Meta-regression results: predictors of log odds ratio

	Mean	Standard error
Expected prevalence term	-25.44	9.57
Constant	1.91	0.38

The variance of the random effects in the meta-regression model, on a log scale, is 0.379.

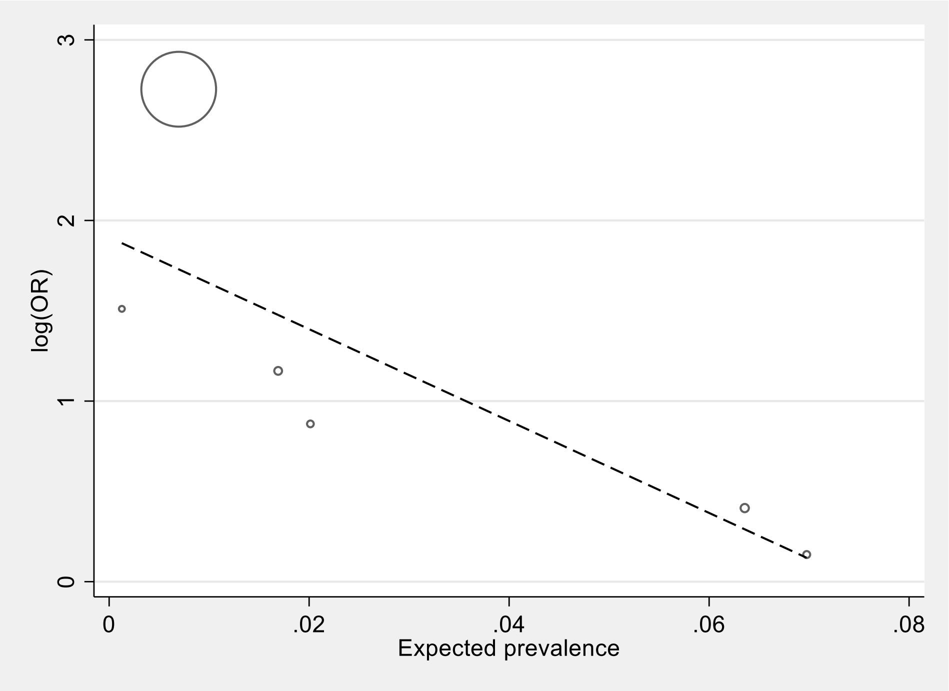

Figure S35: Relationship between expected prevalence (% with both conditions) and log odds ratio

3.41 Hypertension and stroke

Table S41.1: Studies of association between hypertension and ischaemic heart disease

Study	n	Hypertension prevalence	Stroke prevalence	% with both	OR	SE of log(OR)	Expected % with both
Sewpaul 2021	3832	38.4%	2.9%	2.09%	4.33	0.214	1.1%
DHS 2016	8236	44.8%	1.5%	1.07%	3.34	0.205	0.7%
Garin 2016	3836	78.3%	4.9%	4.50%	2.93	0.257	3.9%
Medscheme	1274501	23.8%	0.6%	0.46%	11.25	0.028	0.1%

IHD = ischaemic heart disease, OR = odds ratio (for the association between the two conditions), SE = standard error.

Table S41.2: Meta-regression results: predictors of log odds ratio

	Mean	Standard error
Expected prevalence term	-25.32	20.67
Constant	1.92	0.41

The variance of the random effects in the meta-regression model, on a log scale, is 0.305.

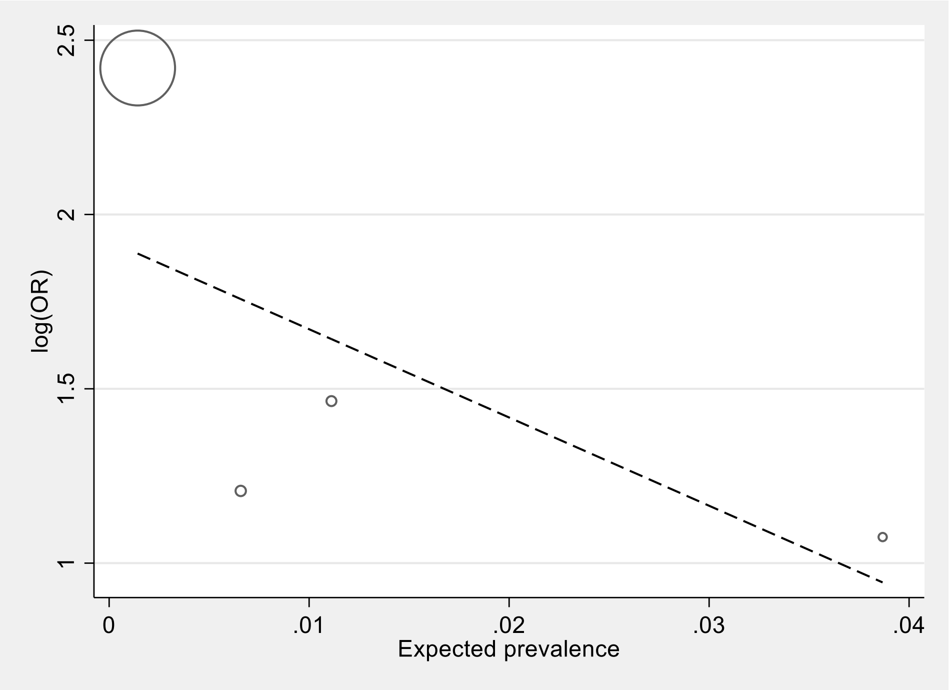

Figure S36: Relationship between expected prevalence (% with both conditions) and log odds ratio

Johnson LF, *et al. BMJ Glob Health* 2024; 9:e013376. doi: 10.1136/bmjgh-2023-013376

3.42 Hypertension and tuberculosis

Table S42.1: Studies of association between hypertension and TB

Study	n	Hypertension prevalence	TB prevalence	% with both	OR	SE of log(OR)	Expected % with both
Wong 2021	17052	25.8%	1.3%	0.33%	0.96	0.155	0.3%
Weimann 2016: wave 1	18526	22.7%	1.6%	0.41%	1.17	0.135	0.4%
Weimann 2016: wave 3	20015	32.1%	0.6%	0.20%	1.08	0.195	0.2%
Oni 2015	14364	64.6%	2.7%	0.82%	0.23	0.112	1.7%
DHS 2016	8256	44.8%	1.5%	0.67%	1.01	0.183	0.7%
Sharman 2019	15023	23.8%	2.8%	1.00%	1.84	0.104	0.7%
PHDC	2568172	20.9%	1.1%	0.16%	0.65	0.017	0.2%

OR = odds ratio (for the association between the two conditions), SE = standard error.

Table S42.2: Meta-regression results: predictors of log odds ratio

	Mean	Standard error
Expected prevalence term	-87.49	38.74
Constant	0.36	0.30

The variance of the random effects in the meta-regression model, on a log scale, is 0.249.

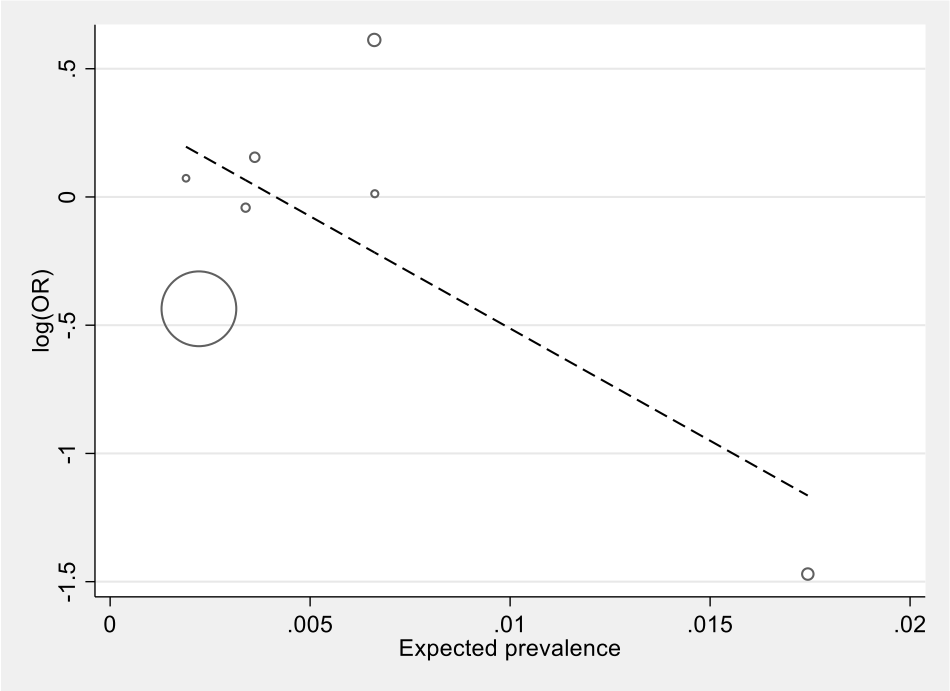

Figure S37: Relationship between expected prevalence (% with both conditions) and log odds ratio

3.43 Ischaemic heart disease and stroke

Table S43.1: Studies of association between ischaemic heart disease (IHD) and stroke

Study	n	IHD prevalence	Stroke prevalence	% with both	OR	SE of log(OR)	Expected % with both
Sewpaul 2021	3832	5.2%	2.9%	0.57%	4.89	0.250	0.15%
DHS 2016	10298	3.4%	1.4%	0.34%	9.84	0.202	0.05%
Garin 2016	3836	8.9%	4.9%	1.22%	3.71	0.179	0.44%
Medscheme	1274501	2.9%	0.6%	0.13%	9.80	0.028	0.02%

IHD = ischaemic heart disease, OR = odds ratio (for the association between the two conditions), SE = standard error.

Table S43.2: Meta-regression results: predictors of log odds ratio

	Mean	Standard error
Expected prevalence term	-242.50	46.10
Constant	2.32	0.03

The variance of the random effects in the meta-regression model, on a log scale, is 0 (i.e. there is no significant heterogeneity across studies when including the expected prevalence term).

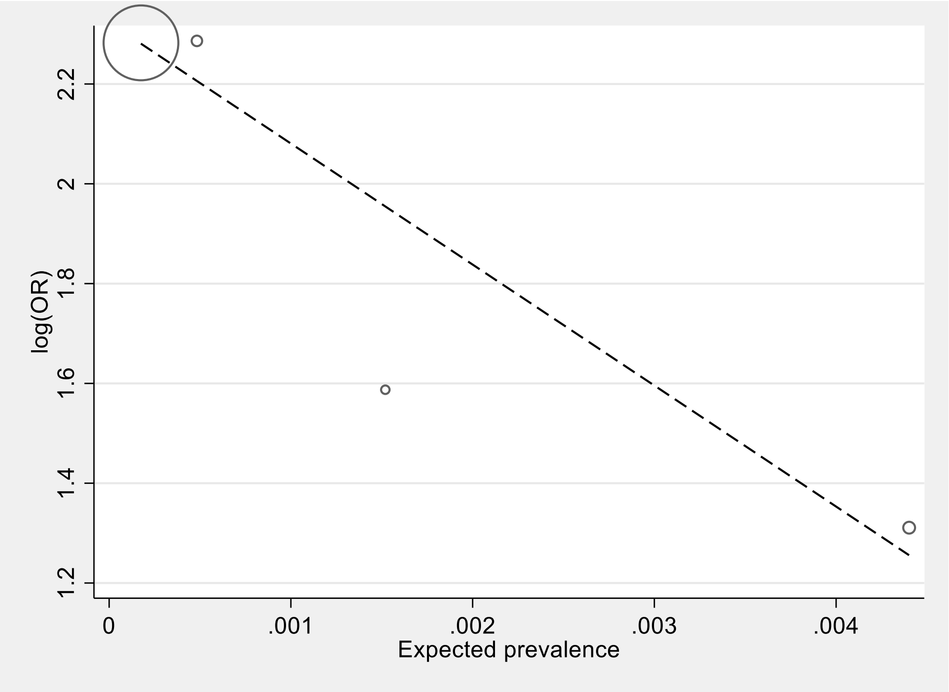

Figure S38: Relationship between expected prevalence (% with both conditions) and log odds ratio

Johnson LF, *et al. BMJ Glob Health* 2024; 9:e013376. doi: 10.1136/bmjgh-2023-013376

3.44 Ischaemic heart disease and tuberculosis

Table S44: Association between ischaemic heart disease (IHD) and TB

Study	n	TB prevalence	IHD prevalence	% with both	OR	SE of log(OR)	Expected % with both
DHS 2016	10305	1.3%	3.4%	0.16%	3.81	0.272	0.05%

OR = odds ratio (for the association between the two conditions), SE = standard error.

3.45 Stroke and tuberculosis

Table S45: Association between stroke and TB

Study	n	TB prevalence	Stroke prevalence	% with both	OR	SE of log(OR)	Expected % with both
DHS 2016	10309	1.3%	1.4%	0.02%	1.02	0.717	0.02%

OR = odds ratio (for the association between the two conditions), SE = standard error.

Johnson LF, *et al. BMJ Glob Health* 2024; 9:e013376. doi: 10.1136/bmjgh-2023-013376

4. Additional analyses

4.1 Results for adults aged 50 and older

	Diabetes	HIV	TB	Depression	COPD	Asthma	IHD	Stroke	Arthritis
Hypertension	3.51 (2.92-4.14)	0.61 (0.56-0.66)	0.69 (0.66-0.73)	1.46 (0.89-2.21)	1.97 (0.78-4)	1.8 (1.02-2.86)	2.2 (0.77-4.78)	3.1 (1.54-5.43)	2.32 (0.68-5.59)
Diabetes		0.58 (0.38-0.82)	0.91 (0.85-0.96)	1.59 (1.27-1.95)	2.05 (0.87-3.97)	1.81 (1.48-2.17)	1.96 (1.26-2.84)	1.72 (1.11-2.48)	2.03 (1.55-2.58)
HIV			4.48 (4.26-4.7)	0.77 (0.56-1.02)	0.51 (0.46-0.57)	0.79 (0.75-0.83)	0.76 (0.27-1.62)	0.68 (0.46-0.96)	0.51 (0.47-0.55)
TB				2.8 (0.92-6.35)	4.13 (2.76-5.84)	2.92 (1.07-6.2)	3.92 (1.74-7.38)	1.21 (0.09-5.06)	
Depression					3.63 (1.43-7.37)	3.6 (1.24-7.92)	2.59 (1.68-3.74)	3 (2.02-4.2)	2.01 (1.92-2.1)
COPD						7.91 (4.04-13.57)	4.74 (2.68-7.58)	3.01 (2.7-3.33)	2.04 (1.85-2.22)
Asthma							3.13 (1.87-4.79)	2.45 (1.51-3.68)	2.36 (2.27-2.46)
IHD								4.14 (3.91-4.36)	2.28 (1.29-3.65)
Stroke									2.06 (1.47-2.76)

Figure S39: Odds ratios representing strength of association between diseases, in adults aged 50 and older

Odds ratios range from less than one (green) to 8 (red). 95% confidence intervals are in brackets. The prevalence levels for individual conditions (in the row and column headings) are the same as in Table 2 of the main text.

Johnson LF, *et al. BMJ Glob Health* 2024; 9:e013376. doi: 10.1136/bmjgh-2023-013376

	Diabetes 24.7%	HIV 15.2%	TB 1.2%	Depression 4.8%	COPD 3.5%	Asthma 5.9%	IHD 9.7%	Stroke 6.7%	Arthritis 28.4%
Hypertension 70.6%	21.4% (18.5-24.2%)	9.3% (8.4-10.1%)	0.8% (0.7-0.8%)	3.6% (1.9-5.4%)	2.8% (2-3.6%)	4.7% (3.7-5.7%)	7.8% (5.3-10.2%)	5.8% (3.7-7.8%)	22.5% (15.2-29.7%)
Diabetes 24.7%		2.5% (1.8-3.4%)	0.3% (0.2-0.3%)	1.6% (0.8-2.4%)	1.3% (0.7-2.1%)	2.1% (1.7-2.7%)	3.6% (2.3-4.9%)	2.3% (1.3-3.4%)	9.8% (7-12.5%)
HIV 15.2%			0.5% (0.5-0.6%)	0.6% (0.3-0.9%)	0.3% (0.2-0.4%)	0.7% (0.6-0.9%)	1.1% (0.5-2.2%)	0.7% (0.4-1.1%)	2.7% (1.9-3.7%)
TB 1.2%				0.1% (0-0.3%)	0.2% (0.1-0.2%)	0.2% (0.1-0.3%)	0.3% (0.2-0.5%)	0.1% (0-0.3%)	
Depression 4.8%					0.5% (0.2-1%)	0.8% (0.3-1.5%)	1.0% (0.5-1.6%)	0.8% (0.4-1.3%)	2.1% (1.1-3.1%)
COPD 3.5%						1.0% (0.6-1.5%)	1.1% (0.7-1.6%)	0.6% (0.4-0.8%)	1.5% (1.1-2.1%)
Asthma 5.9%							1.3% (0.8-2%)	0.8% (0.4-1.3%)	2.7% (2-3.5%)
IHD 9.7%								1.8% (1.1-2.6%)	4.3% (2.6-6.2%)
Stroke 6.7%									2.9% (1.7-4.2%)

Figure S40: Prevalence of common comorbidities in South Africans aged 50 and older
Prevalence levels range from close to zero (green) to 22% (red). 95% confidence intervals are in brackets.

Johnson LF, *et al. BMJ Glob Health* 2024; 9:e013376. doi: 10.1136/bmjgh-2023-013376

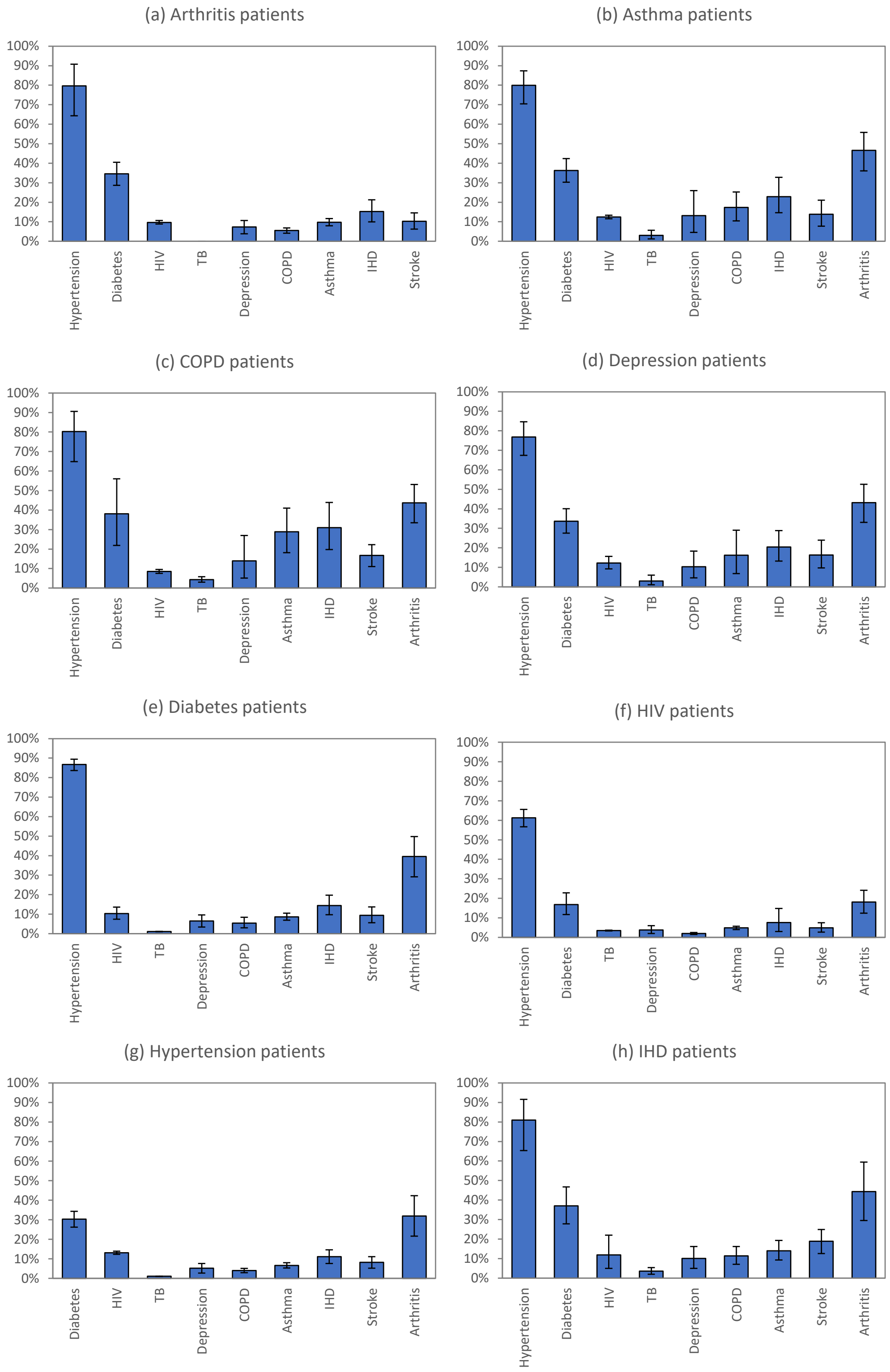

Figure S41: Expected prevalence of different conditions in patients aged 50 and older with each index condition

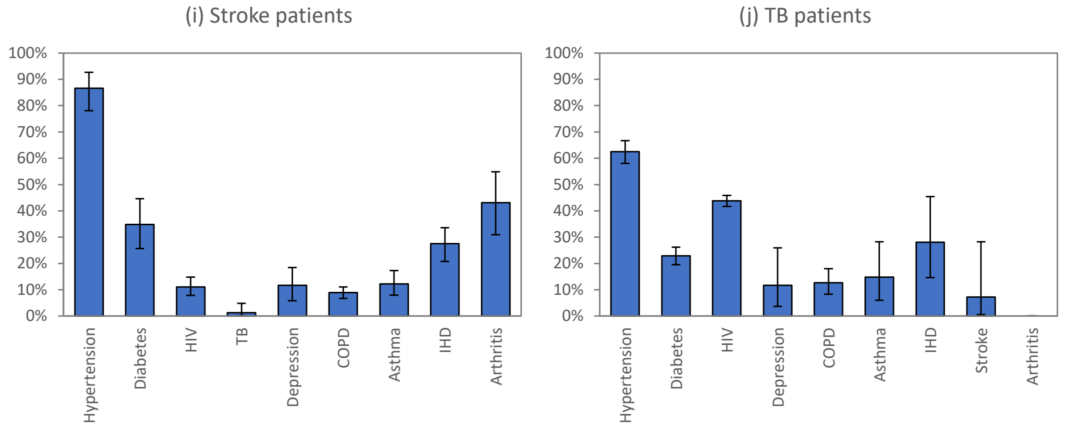

Figure S41 (continued): Expected prevalence of different conditions in patients aged 50 and older with each index condition

4.2 Results for Medscheme

Another approach to assessing how much the observed associations between conditions are due to age confounding is to compare the odds ratios before and after controlling for age. We do this using the Medscheme dataset, (a) because it is the dataset with the most complete coverage of the conditions in which we are interested, and (b) because it is a very large dataset, which allows us to estimate odds ratios with a high degree of precision. Figure S42 compares the unadjusted odds ratios (below-diagonal values) and the adjusted odds ratios (above-diagonal values). Adjusted odds ratios are estimated using multivariable logistic regression models, regressing the condition indicated by the column heading on the condition indicated by the row heading, and controlling for age as well as age-squared. Comparing the below-diagonal and above-diagonal elements, it is clear that in almost all cases, controlling for age leads to attenuated associations (odds ratios closer to 1). The only exception is in the case of HIV and diabetes (where controlling for age leads to a slightly more negative association). However, in almost all cases the associations remain significant when controlling for age. It is only in the case of the association between HIV and COPD, and the association between HIV and stroke, that the association changes direction when controlling for age (in both cases switching from negative to positive).

Figure S43 shows the changes in odds ratios (on the log scale) when going from unadjusted to adjusted models. In almost all cases, adjustment leads to a reduction in the odds ratios. For example, the greatest reduction is in the context of the association between hypertension and COPD, where the odds ratio reduces from 8.38 to 2.00 after adjusting for age (Figure S42), a change of -1.43 in the log of the odds ratio. However, in the case of the associations between HIV and other diseases, controlling for age mostly leads to *increases* in the odds ratios, which is because most of the unadjusted odds ratios are less than one. In the case of asthma and depression, controlling for age made less difference to associations with other conditions, probably because depression and asthma are not as strongly age-related as the other conditions (Table 2 of the main text).

Johnson LF, *et al. BMJ Glob Health* 2024; 9:e013376. doi: 10.1136/bmjgh-2023-013376

	Hypertension	Diabetes	HIV	Depression	COPD	Asthma	IHD	Stroke	Arthritis
Hypertension		6.50 (6.4-6.59)	0.93 (0.91-0.95)	2.11 (2.08-2.15)	2.00 (1.9-2.1)	2.45 (2.4-2.5)	4.11 (4-4.23)	3.75 (3.53-3.99)	2.84 (2.76-2.93)
Diabetes	13.12 (12.95-13.29)		0.84 (0.82-0.86)	1.46 (1.43-1.49)	1.30 (1.24-1.36)	1.93 (1.89-1.97)	2.60 (2.55-2.66)	1.94 (1.85-2.04)	1.78 (1.73-1.83)
HIV	0.92 (0.9-0.93)	0.88 (0.86-0.91)		1.01 (0.98-1.04)	1.14 (1.02-1.26)	0.93 (0.9-0.96)	0.45 (0.42-0.48)	1.21 (1.09-1.33)	0.75 (0.7-0.8)
Depression	2.66 (2.62-2.7)	1.99 (1.96-2.03)	1.05 (1.02-1.08)		1.95 (1.85-2.06)	2.22 (2.17-2.28)	1.66 (1.6-1.71)	2.34 (2.21-2.48)	2.40 (2.32-2.48)
COPD	8.38 (8.02-8.76)	3.43 (3.28-3.58)	0.58 (0.53-0.64)	3.10 (2.94-3.27)		8.82 (8.46-9.21)	2.48 (2.36-2.62)	1.92 (1.72-2.14)	1.47 (1.36-1.58)
Asthma	2.89 (2.84-2.93)	2.54 (2.49-2.59)	0.78 (0.75-0.8)	2.49 (2.43-2.55)	13.62 (13.08-14.19)		1.87 (1.81-1.93)	1.47 (1.37-1.58)	2.40 (2.31-2.49)
IHD	15.29 (14.89-15.7)	6.47 (6.34-6.61)	0.28 (0.26-0.3)	2.57 (2.49-2.65)	9.62 (9.15-10.1)	3.02 (2.93-3.12)		2.89 (2.73-3.07)	1.42 (1.36-1.48)
Stroke	11.25 (10.66-11.87)	4.51 (4.3-4.72)	0.80 (0.73-0.88)	3.55 (3.36-3.76)	6.96 (6.27-7.74)	2.45 (2.29-2.63)	9.80 (9.28-10.36)		1.36 (1.24-1.48)
Arthritis	8.48 (8.24-8.72)	4.15 (4.03-4.26)	0.59 (0.55-0.63)	3.47 (3.36-3.59)	4.68 (4.35-5.04)	3.46 (3.34-3.58)	4.59 (4.41-4.78)	3.96 (3.62-4.32)	

Figure S42: Odds ratios representing strength of association between diseases, comparing unadjusted associations (below diagonal) and age-adjusted associations (above diagonal)

	Diabetes	HIV	Depression	COPD	Asthma	IHD	Stroke	Arthritis
Hypertension	-0.70	0.01	-0.23	-1.43	-0.17	-1.31	-1.10	-1.09
Diabetes		-0.05	-0.31	-0.97	-0.27	-0.91	-0.84	-0.85
HIV			-0.04	0.68	0.18	0.47	0.41	0.24
Depression				-0.46	-0.11	-0.44	-0.42	-0.37
COPD					-0.43	-1.36	-1.29	-1.16
Asthma						-0.48	-0.51	-0.37
IHD							-1.22	-1.17
Stroke								-1.07

Figure S43: Change in odds ratios (on log scale) when controlling for age
The value in each cell represents the difference between the log of the odds ratio when adjusting for age (the above-diagonal entries in Figure S42) and the log of the odds ratio when not adjusting for age (the below-diagonal entries in Figure S42).

For completeness, Table S46 shows the numbers of Medscheme beneficiaries with each disease combination. The most prevalent combination of chronic conditions in this insured population is hypertension and diabetes (101 094 beneficiaries).

Table S46: Numbers of Medscheme adult beneficiaries with each disease combination

	Diabetes	HIV	Depression	COPD	Asthma	IHD	Stroke	Arthritis
Hypertension	101094	19909	35784	7063	31754	30245	5917	17467
Diabetes		8620	15175	2834	15383	15379	2654	7880
HIV			5982	411	3877	794	434	1041
Depression				1709	9573	5411	1487	4569
COPD					4182	2115	379	803
Asthma						5289	940	3926
IHD							1683	2808
Stroke								539

In Table S47 these absolute numbers are expressed as a proportion of the Medscheme population aged 15 and older. In Table S48 the prevalence of each disease combination is expressed as a ratio (relative to the corresponding prevalence estimate at a national level, as shown in Figure 2 of the main text). For most disease combinations, prevalence is estimated to be higher nationally than in the Medscheme database. This is likely to be because (a) the Medscheme estimates relate only to people who are receiving treatment, while the national

estimates include both untreated and treated individuals; (b) sicker and unemployed individuals are less likely to be able to pay for private healthcare; and (c) the prevalence of diseases such as HIV and COPD is believed to be higher in lower socio-economic groups, who are unlikely to be represented in the Medscheme population. However, there are some disease combinations that are more prevalent in the Medscheme population than in the national population; this is particularly the case for disease combinations involving hypertension, diabetes, depression and asthma.

Table S47: Prevalence of disease combinations in Medscheme adult beneficiaries

	Diabetes	HIV	Depression	COPD	Asthma	IHD	Stroke	Arthritis
Hypertension	7.9%	1.6%	2.8%	0.6%	2.5%	2.4%	0.5%	1.4%
Diabetes		0.7%	1.2%	0.2%	1.2%	1.2%	0.2%	0.6%
HIV			0.5%	0.0%	0.3%	0.1%	0.0%	0.1%
Depression				0.1%	0.8%	0.4%	0.1%	0.4%
COPD					0.3%	0.2%	0.0%	0.1%
Asthma						0.4%	0.1%	0.3%
IHD							0.1%	0.2%
Stroke								0.0%

Table S48: Ratio of disease combination prevalence in Medscheme to that in the national population (ages 15 and older)

	Diabetes	HIV	Depression	COPD	Asthma	IHD	Stroke	Arthritis
Hypertension	1.06	0.33	1.40	0.46	1.47	0.64	0.24	0.18
Diabetes		0.56	1.49	0.56	1.72	0.80	0.35	0.25
HIV			0.47	0.16	0.61	0.07	0.09	0.06
Depression				0.45	1.50	0.61	0.29	0.28
COPD					0.55	0.28	0.15	0.11
Asthma						0.69	0.25	0.34
IHD							0.19	0.12
Stroke								0.06

Table S49 shows the average number of comorbidities (out of the 9 conditions considered) for each index condition. These are compared with the averages presented in Figure 4 of the main text. Although the numbers are not exactly comparable, due to TB not being included in the Medscheme database, the prevalence of TB is sufficiently low in the insured population that it is unlikely to have much effect on the results. For all conditions (except arthritis) the modelled number of comorbidities at a national level is greater than that measured in the Medscheme database. This is to be expected, as the Medscheme data reflect individuals who are claiming benefits for their respective conditions, and there are likely to be many members who have undiagnosed or untreated comorbidities.

Table S49: Average number of comorbidities in patients with each index condition, comparing the Medscheme database and national model estimates

	Hyper-tension	Diabetes	HIV	Depression	COPD	Asthma	IHD	Stroke	Arthritis
Medscheme	0.82	1.24	0.46	0.97	1.99	1.08	1.71	1.84	1.60
National*	0.91 (0.81-1.00)	1.50 (1.32-1.68)	0.60 (0.48-0.71)	1.47 (1.24-1.69)	2.33 (2.08-2.59)	1.67 (1.37-1.97)	1.92 (1.65-2.18)	2.04 (1.77-2.30)	1.51 (1.35-1.61)

* From Figure 4 in the main text (95% confidence intervals are in brackets).

4.3 Results when excluding individuals attending health facilities

The five studies that were based on patients attending health facilities were excluded from this analysis. Figure S44 shows the resulting odds ratios, and Figure S45 shows the associated estimates of the prevalence of different disease combinations.

	Diabetes	HIV	TB	Depression	COPD	Asthma	IHD	Stroke	Arthritis
Hypertension	6.4 (4.16-9.29)	0.77 (0.63-0.94)	1.16 (0.59-2.02)	1.67 (1-2.6)	5.24 (2.31-10.03)	2.5 (1.24-4.45)	5.36 (1.81-12.1)	5.98 (2.45-12.14)	5.09 (0.43-20.78)
Diabetes		0.69 (0.33-1.31)	2.26 (0.7-5.57)	1.9 (1.46-2.41)	3.52 (1.3-7.7)	2.34 (0.41-7.36)	3.71 (1.64-7.13)	2.84 (1.24-5.45)	3.5 (3.13-3.83)
HIV			4.85 (1.6-11.19)	1.1 (0.75-1.54)	0.67 (0.42-0.99)	0.73 (0.51-1.02)	0.89 (0.21-2.54)	0.82 (0.67-0.99)	0.57 (0.47-0.68)
TB				4.25 (3.17-5.49)	5.83 (0.83-19.74)	2.55 (1.79-3.46)	3.96 (2.21-6.4)	1.32 (0.24-4.02)	
Depression					3.98 (1.99-6.93)	3.48 (1.05-8.24)	3.2 (1.81-5.17)	4.06 (1.69-8.16)	3.17 (2.9-3.43)
COPD						14.63 (10.28-19.87)	9.18 (8.28-10.16)	5.85 (4.93-6.75)	3.59 (1.71-6.46)
Asthma							4.25 (2.08-7.63)	4 (1.28-9.38)	3.26 (3.07-3.43)
IHD								7.2 (5.85-8.41)	4.38 (4.1-4.66)
Stroke									3.27 (2.07-4.8)

Figure S44: Odds ratios representing strength of association between diseases, based on community-based studies (excluding studies in health facilities)
Odds ratios range from less than one (green) to 15 (red). 95% confidence intervals are in brackets. The prevalence levels for individual conditions (in the row and column headings) are the same as in Table 2 of the main text.

	Diabetes 10.2%	HIV 18.2%	TB 1.0%	Depression 4.9%	COPD 1.8%	Asthma 3.5%	IHD 5.6%	Stroke 2.6%	Arthritis 11.3%
Hypertension 33.9%	7.3% (6.2-8.4%)	5.3% (4.6-6.1%)	0.4% (0.2-0.5%)	2.2% (1.5-2.9%)	1.3% (0.9-1.6%)	1.9% (1.3-2.5%)	3.8% (2.6-5.1%)	1.9% (1.2-2.5%)	6.5% (2.1-10.5%)
Diabetes 10.2%		1.4% (0.8-2.3%)	0.2% (0.1-0.4%)	0.8% (0.6-1.1%)	0.5% (0.2-0.9%)	0.7% (0.2-1.6%)	1.5% (0.9-2.2%)	0.6% (0.3-1%)	2.7% (2.2-3.3%)
HIV 18.2%			0.5% (0.3-0.7%)	1.0% (0.7-1.3%)	0.2% (0.1-0.3%)	0.5% (0.4-0.7%)	0.9% (0.3-2.1%)	0.4% (0.3-0.5%)	1.3% (1-1.7%)
TB 1.0%				0.2% (0.1-0.2%)	0.1% (0-0.3%)	0.1% (0.1-0.1%)	0.2% (0.1-0.3%)	0.0% (0-0.1%)	
Depression 4.9%					0.3% (0.2-0.5%)	0.5% (0.2-1%)	0.7% (0.4-1.1%)	0.4% (0.2-0.7%)	1.3% (1.1-1.6%)
COPD 1.8%						0.6% (0.4-0.7%)	0.6% (0.5-0.7%)	0.2% (0.2-0.3%)	0.5% (0.3-0.8%)
Asthma 3.5%							0.6% (0.4-1.1%)	0.3% (0.1-0.6%)	1.0% (0.8-1.2%)
IHD 5.6%								0.7% (0.5-0.9%)	1.8% (1.4-2.3%)
Stroke 2.6%									0.7% (0.4-1.1%)

Figure S45: Prevalence of common condition combinations in South Africans aged 15 and older, based on community-based studies (excluding studies in health facilities)

Prevalence levels range from close to zero (green) to 8% (red). 95% confidence intervals are in brackets. The prevalence levels for individual conditions (in the row and column headings) are the same as in Table 2.

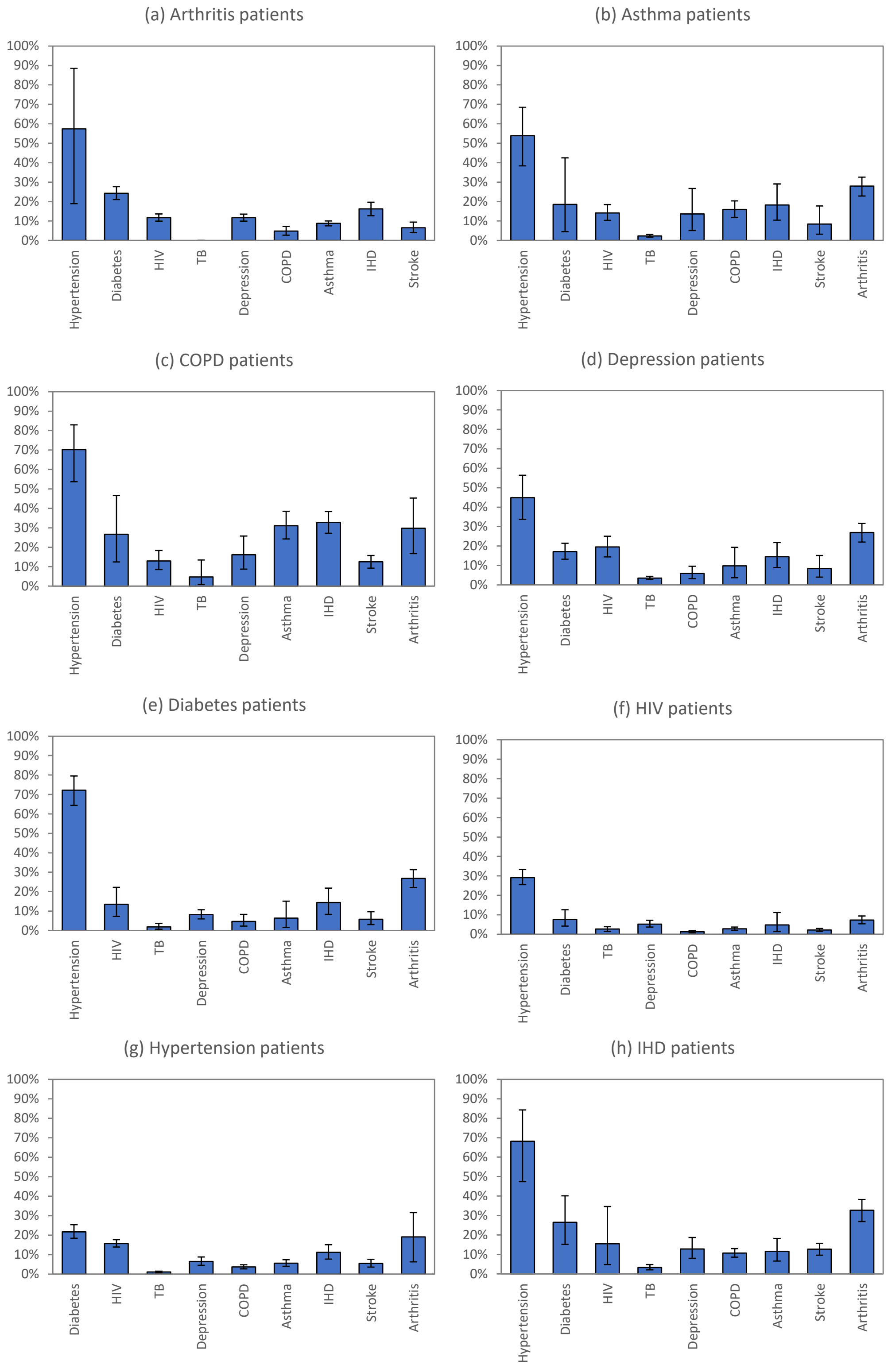

Figure S46: Expected prevalence of different conditions in South Africans aged 15 and older, based on community-based studies (excluding studies in health facilities)

Johnson LF, *et al. BMJ Glob Health* 2024; 9:e013376. doi: 10.1136/bmjgh-2023-013376

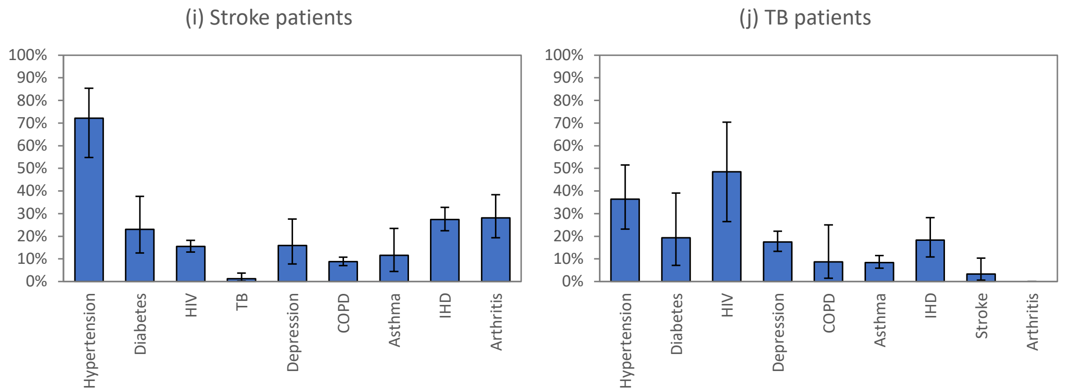

Figure S46 (continued): Expected prevalence of different conditions in South Africans aged 15 and older, based on community-based studies (excluding studies in health facilities)

Figure S47 compares the expected numbers of comorbidities in patients with each index condition, when using only community-based studies, against the results when using all the identified studies (the same results as in Figure 4a of the main text). Results are very similar, although the confidence interval is noticeably wider in the case of arthritis, reflecting the low number of studies in arthritis patients and the consequent loss of precision when excluding studies of patients attending health facilities.

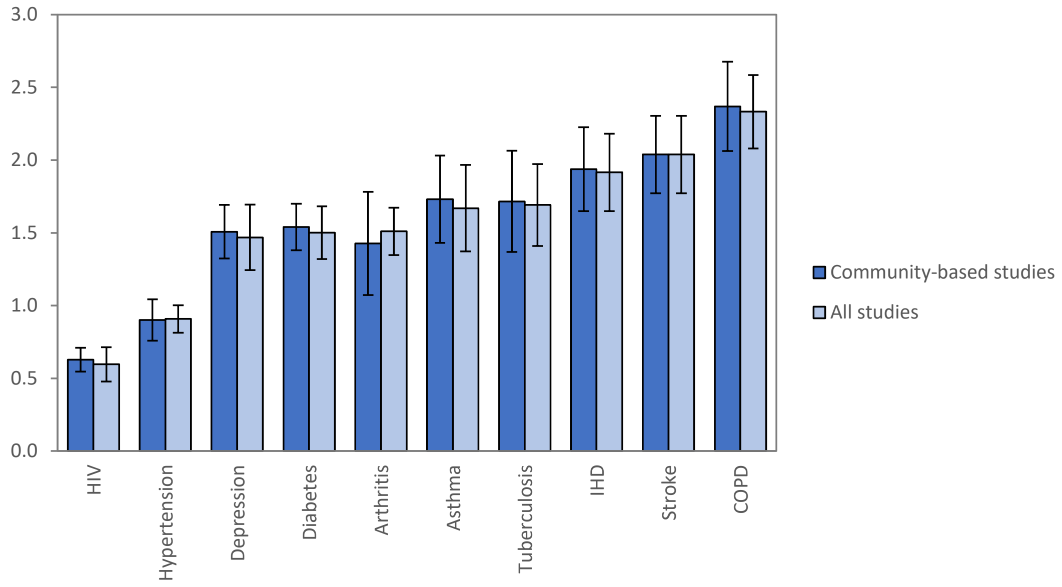

Figure S47: Expected number of comorbidities in patients with each index condition

References

1. Chang AY, Gomez-Olive FX, Payne C, Rohr JK, Manne-Goehler J, Wade AN*, et al.* Chronic multimorbidity among older adults in rural South Africa. *BMJ Glob Health* 2019; **4**:e001386.
2. Pengpid S, Peltzer K. Mental morbidity and its associations with socio-behavioural factors and chronic conditions in rural middle- and older-aged adults in South Africa. *J Psychol Afr* 2020; **30**:257-263.
3. Corbett EL, Charalambous S, Moloi VM, Fielding K, Grant AD, Dye C*, et al.* Human immunodeficiency virus and the prevalence of undiagnosed tuberculosis in African gold miners. *Am J Respir Crit Care Med* 2004; **170**:673-679.
4. Ehrlich RI, White N, Norman R, Laubscher R, Steyn K, Lombard C*, et al.* Wheeze, asthma diagnosis and medication use: a national adult survey in a developing country. *Thorax* 2005; **60**:895-901.
5. Ehrlich RI, White N, Norman R, Laubscher R, Steyn K, Lombard C*, et al.* Predictors of chronic bronchitis in South African adults. *Int J Tuberc Lung Dis* 2004; **8**:369-376.
6. Grimsrud A, Stein DJ, Seedat S, Williams D, Myer L. The association between hypertension and depression and anxiety disorders: results from a nationally-representative sample of South African adults. *PLoS One* 2009; **4**:e5552.
7. Jithoo A. Respiratory symptoms and chronic obstructive pulmonary disease: Prevalence and risk factors in a predominantly low-income urban area of Cape Town, South Africa. Cape Town: University of Cape Town; 2006.
8. Middelkoop K, Bekker LG, Myer L, Whitelaw A, Grant A, Kaplan G*, et al.* Antiretroviral program associated with reduction in untreated prevalent tuberculosis in a South African township. *Am J Respir Crit Care Med* 2010; **182**:1080-1085.
9. Negin J, Martiniuk A, Cumming RG, Naidoo N, Phaswana-Mafuya N, Madurai L*, et al.* Prevalence of HIV and chronic comorbidities among older adults. *AIDS* 2012; **26 (Suppl 1)**:S55-63.
10. Pengpid S, Peltzer K. Depression symptoms: Their association with socio-demographic factors and health among adults in South Africa. *J Psychol Afr* 2018; **28**:62-65.
11. Garin N, Koyanagi A, Chatterji S, Tyrovolas S, Olaya B, Leonardi M*, et al.* Global multimorbidity patterns: a cross-sectional, population-based, multi-country study. *J Gerontol Med Sci* 2016; **71**:205-214.
12. Ho IS, Azcoaga-Lorenzo A, Akbari A, Black C, Davies J, Hodgins P*, et al.* Examining variation in the measurement of multimorbidity in research: a systematic review of 566 studies. *Lancet Public Health* 2021; **6**:e587-e597.
13. Ezzati M, Lopez AD, Rodgers A, Vander Hoorn S, Murray CJ. Selected major risk factors and global and regional burden of disease. *Lancet* 2002; **360**:1347-1360.
14. Lalkhen H, Mash R. Multimorbidity in non-communicable diseases in South African primary healthcare. *S Afr Med J* 2015; **105**:134-138.
15. Folb N, Timmerman V, Levitt NS, Steyn K, Bachmann MO, Lund C*, et al.* Multimorbidity, control and treatment of noncommunicable diseases among primary healthcare attenders in the Western Cape, South Africa. *S Afr Med J* 2015; **105**:642-647.
16. Petersen I, Rathod S, Kathree T, Selohilwe O, Bhana A. Risk correlates for physical-mental multimorbidities in South Africa: a cross-sectional study. *Epidemiol Psychiatr Sci* 2019; **28**:418-426.

17. van Heerden A, Barnabas RV, Norris SA, Micklesfield LK, van Rooyen H, Celum C. High prevalence of HIV and non-communicable disease (NCD) risk factors in rural KwaZulu-Natal, South Africa. *J Int AIDS Soc* 2017; **20**.
18. Oni T, Youngblood E, Boulle A, McGrath N, Wilkinson RJ, Levitt NS. Patterns of HIV, TB, and non-communicable disease multi-morbidity in peri-urban South Africa-a cross sectional study. *BMC Infect Dis* 2015; **15**:20.
19. Sewpaul R, Mbewu AD, Fagbamigbe AF, Kandala N, Reddy SP. Prevalence of multimorbidity of cardiometabolic conditions and associated risk factors in a population-based sample of South Africans: A cross-sectional study. *Public Health Pract* 2021; **2**:100193.
20. Sharman M, Bachmann M. Prevalence and health effects of communicable and non-communicable disease comorbidity in rural KwaZulu-Natal, South Africa. *Trop Med Int Health* 2019; **24**:1198-1207.
21. Weimann A, Dai D, Oni T. A cross-sectional and spatial analysis of the prevalence of multimorbidity and its association with socioeconomic disadvantage in South Africa: A comparison between 2008 and 2012. *Soc Sci Med* 2016; **163**:144-156.
22. Wong EB, Olivier S, Gunda R, Koole O, Surujdeen A, Gareta D*, et al.* Convergence of infectious and non-communicable disease epidemics in rural South Africa: a cross-sectional, population-based multimorbidity study. *Lancet Glob Health* 2021; **9**:e967-e976.
23. Hayward SE, Deal A, Rustage K, Nellums LB, Sweetland AC, Boccia D*, et al.* The relationship between mental health and risk of active tuberculosis: a systematic review. *BMJ Open* 2022; **12**:e048945.
