# Supplementary material for: A model-based approach to estimating the prevalence of disease combinations in South Africa
Source: BMJ Glob Health. 2024 Feb 22;9(2):e013376. doi: 10.1136/bmjgh-2023-013376 (PMC10884267; doi:10.1136/bmjgh-2023-013376)
Supplement: Supplementary data [file bmjgh-2023-013376supp002.pdf]

Supplementary material: Why the strength of association between two conditions depends on the prevalence of both conditions

Consider two hypothetical populations: population 1 and population 2 (figure). In both populations we consider associations between three conditions, A, B and C. In population 1, each condition has a prevalence of 7%, 1% of people have all three conditions, and 3% of people have two of the three conditions. In population 2, all of these proportions are doubled: each condition has a prevalence of 14%, 2% of people have all three conditions, and 6% of people have two of the three conditions. The relative levels of overlap between the different conditions are thus the same in the two populations, i.e. the conditions exhibit the same pattern of clustering. However, the odds ratio (OR) for the association between condition A and condition B is 7.04 ((0.02/0.05)/(0.05/0.88)) in population 1 compared to 3.04 ((0.04/0.10)/(0.10/0.76)) in population 2. If we were interested in measuring the OR in the sub-population of population 2 that has any of the three conditions (as a crude approximation to what might be expected if we were assessing the association in people attending health facilities), the OR for the association between condition A and condition B is 0.32 ((0.04/0.10)/(0.10/0.08)); in this sub-population the prevalence of condition A is 43.8% (0.14/0.32), the same as for condition B. Thus the OR is highly sensitive to the prevalence of the disease in the population (or sub-population) in which it is being measured.

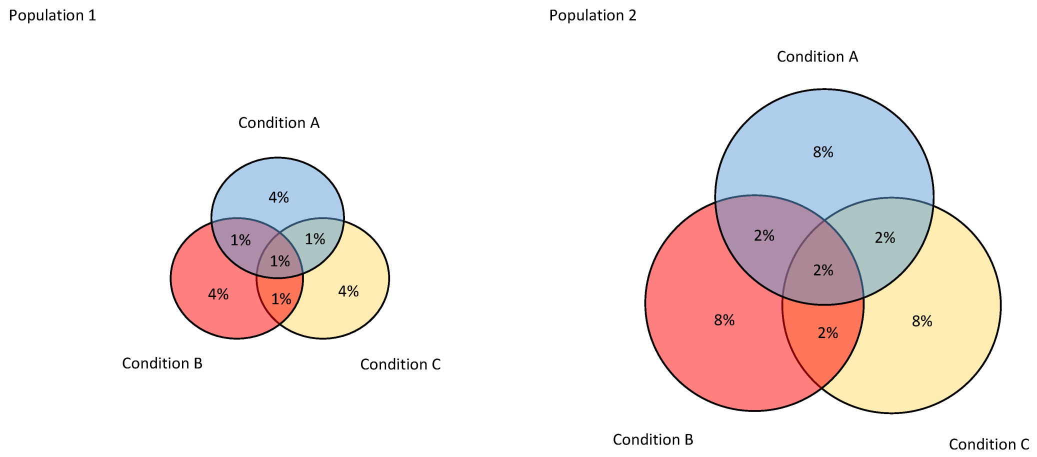

The differences in prevalence between population 1 and population 2 could be due to a number of factors. For example, population 1 might be a sample of all adults, while population 2 might be a sample of adults aged 60 and older. Alternatively, population 1 might represent a study in which less sensitive self-reporting of past diagnosis is used to measure prevalence, while population 2 might represent a study in which more accurate clinical measures are used. Differences in socio-economic status, urban-rural differences and differences in study design (e.g. case-control versus cross-sectional survey) could also all account for differences in prevalence.
